# Meta-analysis of cotton fiber quality QTLs across diverse environments in a *Gossypium hirsutum *x *G. barbadense *RIL population

**DOI:** 10.1186/1471-2229-10-132

**Published:** 2010-06-28

**Authors:** Jean-Marc Lacape, Danny Llewellyn, John Jacobs, Tony Arioli, David Becker, Steve Calhoun, Yves Al-Ghazi, Shiming Liu, Oumarou Palaï, Sophie Georges, Marc Giband, Henrique de Assunção, Paulo Augusto Vianna Barroso, Michel Claverie, Gérard Gawryziak, Janine Jean, Michèle Vialle, Christopher Viot

**Affiliations:** 1 UMR-DAP, CIRAD, Avenue Agropolis, 34398, Montpellier Cedex 5, France; 2 CSIRO Plant Industry, P.O. Box 1600 Canberra, ACT, Australia; 3 Bayer BioScience N.V., Technologiepark 38, Ghent, Belgium; 4 Bayer CropScience, BioScience research, Lubbock, TX, USA; 5 IRAD, Centre Régional de Recherche Agricole de Maroua, BP 33 Maroua, Cameroon; 6 EMBRAPA Algodão, Rua Osvaldo Cruz 1143, Centenario, 58.428-095 Campina Grande, PB, Brazil; 7 UPR-SCA, CIRAD, Avenue Agropolis, 34398, Montpellier Cedex 5, France

## Abstract

**Background:**

Cotton fibers (produced by *Gossypium *species) are the premier natural fibers for textile production. The two tetraploid species, *G. barbadense *(Gb) and *G. hirsutum *(Gh), differ significantly in their fiber properties, the former having much longer, finer and stronger fibers that are highly prized. A better understanding of the genetics and underlying biological causes of these differences will aid further improvement of cotton quality through breeding and biotechnology. We evaluated an inter-specific *Gh *× *Gb *recombinant inbred line (RIL) population for fiber characteristics in 11 independent experiments under field and glasshouse conditions. Sites were located on 4 continents and 5 countries and some locations were analyzed over multiple years.

**Results:**

The RIL population displayed a large variability for all major fiber traits. QTL analyses were performed on a per-site basis by composite interval mapping. Among the 651 putative QTLs (LOD > 2), 167 had a LOD exceeding permutation based thresholds. Coincidence in QTL location across data sets was assessed for the fiber trait categories strength, elongation, length, length uniformity, fineness/maturity, and color. A meta-analysis of more than a thousand putative QTLs was conducted with MetaQTL software to integrate QTL data from the RIL and 3 backcross populations (from the same parents) and to compare them with the literature. Although the global level of congruence across experiments and populations was generally moderate, the QTL clustering was possible for 30 trait x chromosome combinations (5 traits in 19 different chromosomes) where an effective co-localization of unidirectional (similar sign of additivity) QTLs from at least 5 different data sets was observed. Most consistent meta-clusters were identified for fiber color on chromosomes c6, c8 and c25, fineness on c15, and fiber length on c3.

**Conclusions:**

Meta-analysis provided a reliable means of integrating phenotypic and genetic mapping data across multiple populations and environments for complex fiber traits. The consistent chromosomal regions contributing to fiber quality traits constitute good candidates for the further dissection of the genetic and genomic factors underlying important fiber characteristics, and for marker-assisted selection.

## Background

There are two economically important tetraploid cultivated species of cotton, *Gossypium hirsutum *(also referred to as "Upland" cotton) and *G. barbadense *(Caribbean "Sea-Island", Extra Long Staple "ELS" and modern "Pima" and "Egyptian" cultivars). They display many complementary agronomic features. *G. hirsutum *(hereafter *Gh*), the most widely cultivated species, has higher yield potential than *G. barbadense (Gb) *in most environments; however, *Gb *cultivars are superior to *Gh *in most aspects of fiber quality, such as fiber length, strength and fineness. The two species are derived from a recent polyploidization event that occurred ~1-2 MYA [1]. They are inter-fertile, but inter-specific crosses aimed at recombination of of genes underlying their complementary agronomic performance have generally resulted in difficulties such as reduced fertility, cytological abnormalities and distorted segregation in the F _2 _generation. Although inter-specific breeding by conventional approaches has had positive impacts on cultivar development [2], the potential for using molecular markers to facilitate more rapid and effective selection and transfer of *G. barbadense *fiber properties to *G. hirsutum *[3] is clear.

Cotton fibers are highly elongated single cells of the epidermal layer of the seed. Fiber development spans four discrete, yet overlapping stages: initiation (-3 to 5 days post anthesis, dpa), elongation (3 to 21 dpa), secondary cell wall deposition (14 to 45 dpa) and maturation/dehydration (40 to 55 dpa) [4]. The final mature spinnable fibers are dried flattened cylinders ~35-50 mm long and made of ~96% cellulose. Their commercial value is determined by their overall physical dimensions and the extent of thickening of the internal walls. Various physical properties of cotton fibers are measured ranging from fiber length and length uniformity, strength, elongation (degree of extensibility), maturity (extent of cell wall thickening), micronaire (resistance to air flow across a plug of fibers) and fineness (linear density, a function of diameter and thickness), to color indices (reflectance and yellowness). The most commonly used equipment, High Volume Instrument (HVI), is used for commercial and research applications for high-throughput measurements of most of these parameters, while research programs also rely on specialized instruments like Fibronaire, Maturimeter or the Advanced Fiber Information System (AFIS) [5]. An optimal fiber quality results from the composite association of numerous partially correlated quantitative traits all impacting on the final performance of the fiber in spinning and weaving and relatively small differences in fiber parameters can attract premiums or penalties during marketing.

Earlier genetic studies demonstrated fairly high heritabilities of fiber characteristics, in excess of 0.40, except for length uniformity [5-7], and moderate environment and GxE effects (also reviewed in [5]). More than 30 reports have been published on genetic mapping and QTL mapping in inter-specific *Gh *× *Gb *populations, of which 13, originating from 14 different populations, relate to QTL data for fiber traits. These studies represent a total of over 455 QTLs [6-18]. A common feature of most of these QTL studies is that positive effects on all fiber traits are derived from the presence of alleles from both *Gh *and *Gb *parents [6,7,17] commonly resulting in transgressive segregation [7,19].

Comparison between different QTL studies in cotton is generally complicated by insufficient marker synteny between maps and the lack of sufficient bridge markers. However, an overall observation is that the level of congruence of QTL localization is generally low. Only two QTL reports have included a comparison across populations. The study by Lacape *et al. *[7], focusing on 3 backcross generations (BC _1_, BC _2 _and BC _2_S _1_, derived from the *Gh *× *Gb *cross of Guazuncho-2 *(Gh) *× VH8-4602 *(Gb)*), reported 50 significant fiber QTLs (80 putative QTLs at LOD > 2.5). Only 20% of the QTLs were common between at least 2 of the 3 BC data sets and only 30% putatively agreed with at least one QTL report in the literature for both chromosomal location and parental species origin. Rong *et al. *[16] aligned 212 fiber QTLs from 5 different inter-specific *Gh *× *Gb *populations using the palmeri x K101-F2 (*Gh *× *Gb*) map as a reference. These authors concluded that there was a poor level of correspondence of fiber QTLs between experiments and populations. Inter-specific chromosome substitution lines (having a single *Gh *chromosome pair substituted by *Gb *chromosomes), so called CS-B lines, have been employed to assign genetic effects (additivity, dominance) on fiber traits to specific chromosomes or chromosome arms [20,21], although correlations with other QTL studies were not undertaken.

Although the number of reports on QTL mapping of agronomically important traits in plants has increased tremendously in the past 20 years, several authors have also emphasized limitations and biases of QTL mapping that have limited their broad application to marker-assisted breeding in many crops. It appears that numerous "resource-limited" QTL studies may be questionable in terms of their reliability and accuracy, major limitations being the number of genotypes and number of environments under study [22-25]. Such limitations will be inflated for traits of low to moderate heritability or for traits with significant QTL x environment interactions [26]. A way of improving the power and accuracy in detection of true QTLs is by increasing population sizes (often not practical), or by multiplying the number of environments in which the population is evaluated (reviewed in [26]). One option would be to reanalyze raw data in a pooled analysis [27], but this approach is generally prohibitive because of different data structures and requirement for a common set of markers across populations.

Comparative QTL mapping and meta-analysis, however, provides another means to unify, and thereby simplify molecular analysis of complex phenotypes across multiple data sets. Meta-analysis aims to study QTL congruency. Results are pooled across studies in order to combine them in a single result, thus improving estimate in QTL detection [28]. Meta-analysis of QTLs has mainly been reported in medical and animal sciences [29]. Examples in plants include nematode resistance in soybean [30], disease resistance traits in cocoa [31], drought-related traits in rice [32] and ear emergence in wheat [33]. The report on cotton by Rong *et al. *[16] concerned yield and fiber traits gathered from diverse QTL mapping reports that were collectively projected onto a reference map. These studies essentially used BioMercator software [34], that has recently been followed by a new computational and statistical package, MetaQTL [35]. MetaQTL has been used for the meta-analysis of QTLs for flowering time [35] and N-remobilization in maize [36], FHB resistance in wheat [37], and root architecture in rice [38].

In this study, we evaluated the fiber characteristics of a population of inter-specific *Gh *× *Gb *RILs in eleven independent experiments involving both field and glasshouse conditions, different locations and multiple years. The two parents used for the RIL population were the same as those being currently used in marker-assisted backcross selection underway at CIRAD to enhance the fiber quality of *Gh *breeding material [7]. A meta-analysis was undertaken with MetaQTL using the fiber QTLs derived from the eleven experimental sites of the RIL population, fiber QTLs previously reported in backcross populations, and fiber QTLs from the literature, in order to identify highly congruent genomic regions contributing to key fiber quality traits.

## Results

### Trait variation

Summary data of the 11 experiments and the fiber trait values for the parents and RILs are given in Table 1 and Table 2, respectively. Among the 11 fiber data sets, 4 were collected from glasshouse grown plants and 7 from field-grown plants (Table 1). Although field-grown samples as compared to glasshouse samples may be expected to be more representative of real commercial growing conditions, they may also be more prone to environmental variation. The 2 types of growing conditions were therefore not specifically separated in all further analyses. The two parents, Guazuncho-2 (*Gh*) and VH8-4602 (*Gb*) display a broad range of phenotypes in many aspects of plant growth and development (not shown) as well as fiber quality parameters (Table 2), making them suitable for discriminating the underlying genetic contributions to fiber quality in genetic crosses. The fibers of the *Gb *parent were intrinsically very fine (favorable), but their maturity and micronaire measures were usually very low (unfavorable). As expected, the RILs displayed a high degree of variability at all sites and for every descriptor of plant phenology (such as earliness), morphology (eg., leaf shape and hairiness), and production components (eg., boll size or number), but also showed frequent segregation beyond parental variability, or transgression, for those parameters (to be presented elsewhere). Variation among the RILs was also considerable for all fiber quality parameters (Table 2). Although the mean RIL values (intermediate between the 2 parents) were always closer to the *Gh *parent value than to the *Gb *parent, there were always some individual RILs displaying transgression on both sides of the distribution for most traits, as shown by the maximum and minimum values in Table 2. The ranking of individual RILs for fiber characteristics showed good consistency among sites, indicative of moderate GxE interaction effects and high heritabilities for fiber traits, as described below.

**Table 1:** Details of the 11 sites at which the RILs were evaluated

Experiment	Bayer	CSIRO	CIRAD	Brazil	Cameroon	USA	Australia	CIRAD	USA	Brazil	Australia
	Gent	Canberra	Mpellier	Itatuba	Garoua	Lubbock	Narrabri	Mpellier	Idalou	Itatuba	Narrabri
**Acronym**	**Ge6**	**Cs7**	**Mp7**	**Br7**	**Ga7**	**Lu7**	**Cs8**	**Mp8**	**Lu8**	**Br8**	**Cs9**

Planting	Jun-05	Jun-05	-	Aug07	Jul07	May07	Oct07	May07+08	May08	Aug08	Oct08

No RILs sown	139	139	145	128	110	77	93	130	129	128	82

	Glasshouse	Glasshouse	Glasshouse	Field	Field	Field	Field	Glasshouse	Field	Field	Field
Layout	1 plant	1 plant	1 plant	8 m	4 m	9 m	10 m	1 plant	10 m	8 m	12 m
	1 pot	1 pot	1 pot	2 reps	1 rep	1 rep	1-3 reps	2 pots	1 rep	2 reps	1-3 reps

No fib.analyses	38	99	96	123	84	68	65	67	90	128	66

HVI	x		x	x	x	x	x	x	x	x	x
FMT		x		x	x	x	x	x		x	x
color	x		x	x	x	x		x		x	
AFIS		x				x			x		

Planting date and conditions (glasshouse/field), number of RILs planted, and analyzed, fiber testing instruments or modules used to assess traits for QTL mapping.

**Table 2:** Phenotypic variation of fiber traits

Fiber trait category	Fiber trait acronym (unit)	Experimental site	Gua	VH8	No. RILs	RIL mean	RIL sd	RIL max	RIL min
**Fineness**	fineness, H (mtex)	Br7	168.3	109.8	120	148.5	25.0	213.0	81.0
		Br8	161.6	105.8	125	147.9	26.3	216.5	60.0
		Cs7	169.0	140.0	99	155.6	22.0	202.0	110.0
		Cs8	144.5	90.5	65	140.4	20.1	181.1	92.9
		Cs9	155.2	94.3	66	153.7	20.8	192.2	99.6
		Ga7	168.5	62.0	79	132.3	24.6	173.0	61.0
		Lu7	154.0	125.0	68	149.1	16.1	171.0	104.5
		Lu8	168.0	117.5	90	154.7	19.5	183.0	103.0
		Mp8	179.0	123.5	65	157.8	24.2	202.0	87.0

		**Mean all expts**	**163.1**	**107.6**		**148.9**			

	Standard Fineness, Hs	Br7	165.0	175.0	120	165.6	30.7	271.0	102.5
	(mtex)	Br8	162.6	178.6	125	161.7	31.5	280.0	96.5
		Cs7	179.0	146.0	99	164.3	16.8	200.0	133.0
		Cs9	158.4	124.5	66	162.5	18.0	198.1	127.1
		Ga7	231.0	114.0	79	210.2	40.6	308.0	109.0
		Lu7	177.0	146.0	68	170.8	13.4	194.3	136.6
		Mp8	259.3	182.3	65	208.0	42.4	379.0	134.0

		**Mean all expts**	**190.3**	**152.3**		**177.6**			

	Micronaire, IM	Br7	4.43	2.26	120	3.72	0.87	5.45	1.43
		Br8	4.21	2.11	125	3.77	0.87	5.53	1.37
		Cs8	3.69	2.35	65	3.40	0.66	4.59	2.24
		Cs9	4.30	2.80	66	4.21	0.54	5.06	2.89
		Ga7	3.78	1.89	96	2.95	0.62	4.67	1.85
		Ge6	4.53	2.45	24	3.86	0.69	5.00	2.50
		Lu7	4.11	2.68	63	3.71	0.67	4.55	2.10
		Lu8	3.80	2.10	70	3.85	0.60	4.80	2.30
		Mp7	4.05	NA	96	3.67	0.76	5.46	1.90
		Mp8	3.60	2.62	65	3.45	0.72	4.77	1.52

		**Mean all expts**	**4.1**	**2.4**		**3.7**			

	Maturity Ratio, MR	Br7	1.02	0.63	120	0.92	0.18	1.23	0.42
		Br8	1.00	0.59	125	0.95	0.18	1.25	0.39
		Cs7	0.94	0.96	99	0.94	0.06	1.05	0.79
		Cs8	0.91	0.61	65	0.83	0.12	1.03	0.49
		Cs9	0.98	0.76	66	0.95	0.05	1.03	0.78
		Ga7	0.74	0.54	79	0.65	0.15	0.98	0.39
		Lu7	0.87	0.86	68	0.87	0.05	0.94	0.75
		Lu8	0.90	0.80	90	0.88	0.06	0.96	0.71
		Mp7	0.86	NA	96	0.86	0.04	0.96	0.75
		Mp8	0.71	0.68	65	0.78	0.15	1.08	0.39

**Fineness**	**Maturity Ratio, MR**	**Mean all expts**	**0.89**	**0.72**		**0.86**			
**Length**	Mean Length, ML	Br7	24.28	35.86	123	25.89	2.58	31.79	18.70
	(mm)	Br8	25.29	35.35	126	26.15	2.52	31.66	18.43
		Cs8	26.15	29.75	65	25.42	2.13	30.69	19.91
		Ga7	24.06	30.50	83	24.24	2.52	29.39	16.99
		Ge6	26.20	32.80	38	26.07	2.43	30.95	17.62
		Lu7	24.20	32.30	63	24.95	2.41	28.65	18.03
		Mp7	24.00	NA	96	26.53	2.44	32.81	19.69
		Mp8	25.88	36.10	67	25.91	2.37	30.96	20.85

		**Mean all expts**	**25.0**	**33.2**		**25.6**			

	Length, UHML (mm)	Br7	28.89	40.79	123	30.71	2.73	37.55	22.85
		Br8	29.76	40.46	126	30.93	2.65	37.45	23.35
		Cs7	32.60	48.80	99	32.57	2.99	41.15	24.38
		Cs8	30.52	35.84	65	30.33	2.02	35.77	25.58
		Cs9	29.88	37.67	66	31.34	1.78	35.87	28.17
		Ga7	28.65	37.60	83	29.19	2.70	35.50	22.80
		Ge6	30.80	38.20	38	30.99	2.02	36.32	26.92
		Lu7	29.30	39.00	63	30.40	2.32	35.31	24.89
		Lu8	30.00	38.74	70	31.35	1.98	37.08	27.43
		Mp7	28.70	NA	96	31.62	2.49	37.84	25.54
		Mp8	30.43	40.55	67	30.83	2.40	36.30	25.30

		**Mean all expts**	**30.0**	**39.8**		**30.9**			

**Length uniformity**	Uniformity Index, UI	Br7	84.00	87.91	123	84.28	2.34	88.00	76.05
		Br8	84.98	87.36	126	84.50	2.50	88.25	75.30
		Cs8	85.60	83.00	65	83.74	2.46	88.04	76.04
		Cs9	83.40	85.00	66	83.74	1.46	86.42	79.45
		Ga7	83.95	81.10	83	83.03	2.44	87.50	72.90
		Lu8	83.20	85.10	70	85.71	1.60	88.10	81.00
		Mp8	85.00	89.00	67	83.94	2.15	88.40	78.50

		**Mean all expts**	**84.3**	**85.5**		**84.1**			

**Strength**	Strength (g/tex)	Br7	29.64	47.10	123	32.02	4.26	42.45	20.95
		Br8	30.69	49.39	126	32.59	4.62	46.35	21.00
		Cs8	31.16	35.81	65	32.67	2.64	39.23	26.44
		Cs9	31.30	41.60	66	33.26	3.06	41.78	27.14
		Ga7	29.05	39.30	83	30.76	4.19	44.80	20.40
		Ge6	34.10	41.00	38	33.65	3.57	40.20	25.80
		Lu7	30.30	40.50	63	29.71	4.44	37.70	18.40
		Lu8	25.00	31.90	70	28.21	2.76	38.30	23.20
		Mp7	28.80	NA	96	31.85	3.95	43.10	21.40
		Mp8	29.80	46.30	67	31.68	3.01	40.30	22.50

		**Mean all expts**	**30.0**	**41.4**		**31.6**			

**Elongation**	Elongation	Br7	5.1	4.7	123	5.2	0.5	6.8	3.5
		Br8	5.0	4.8	126	5.1	0.5	6.4	3.7
		Cs8	4.6	3.3	65	4.3	0.4	5.2	3.6
		Cs9	3.7	NA	66	3.4	0.9	5.6	1.5
		Ga7	4.9	4.3	83	4.9	0.5	6.1	3.7
		Lu8	5.2	3.6	70	6.3	1.5	10.1	2.8
		Mp8	5.5	4.9	67	5.5	0.6	6.4	4.0

		**Mean all expts**	**4.9**	**4.3**		**5.0**			

**Color**	Reflectance, Rd	Br7	78.8	81.3	123	77.3	4.1	84.6	64.5
		Br8	82.3	80.1	126	78.7	4.1	85.6	67.1
		Ga7	75.6	75.0	84	74.4	3.4	80.2	63.1
		Ge6	82.4	78.6	38	77.4	3.6	84.0	71.1
		Lu7	74.9	82.1	63	76.6	2.9	83.5	67.3
		Mp7	77.7	NA	96	77.0	5.1	84.1	59.2
		Mp8	77.8	81.2	66	78.1	3.9	84.1	63.5

		**Mean all expts**	**78.5**	**79.7**		**77.1**			

	Yellowness, b	Br7	8.6	8.9	123	9.2	1.4	13.2	6.4
		Br8	8.7	8.2	126	8.8	1.3	12.1	6.1
		Ga7	10.6	10.3	84	10.6	1.3	15.2	8.6
		Ge6	8.0	8.7	38	8.4	1.2	10.8	6.3
		Lu7	7.9	8.7	63	8.8	1.2	12.2	7.0
		Mp7	9.6	NA	96	9.3	1.3	13.9	6.9
		Mp8	9.0	8.7	66	9.4	1.2	14.0	7.6

		**Mean all expts**	**8.9**	**8.9**		**9.2**			

Phenotypic variation of fiber traits measured on the 2 parents and the RILs from 11 experiments: mean trait value of parents Guazuncho-2 (GUA) (*Gh*) and VH8-4602 (VH8)(*Gb*), number of RILs analyzed, and mean, standard deviation and maximum and minimum values for the RILs for each trait

Fiber fineness components of the RILs in particular, displayed a wide range of variation. Fiber maturity was considerably improved in the RIL material, relative to the mid-parent value, with an average level close to that of Guazuncho-2 (MR = 0.86 for the RILs, as compared to 0.89 for Guazuncho-2 and 0.72 for VH8-4602 over 10 data sets). In addition to their large variation, fiber length traits (ML and UHML) were the parameters for which the mean RIL values were the most biased toward the *Gh *parent. Fiber of the RILs was on average 1 mm longer (UHML) than that of the *Gh *parent, although the 2 parents differed by more than 8 mm. Fiber strength displayed transgression in some RILs, as exemplified by the extreme high 44.8 g/tex (+ 5.3 g/tex over best parent) for one RIL in experiment Garoua 2007 (Ga7). Regarding the two color parameters, the parents only displayed moderate differences (*Gb *slightly more brilliant) and the variation over the RILs largely exceeded the parental range in both yellowness and reflectance.

### Analysis of variance, trait heritabilities and correlations

A separate analysis of variance of the fiber data collected from the two Brazilian experiments of 2007 and 2008 was conducted. The 2 randomized blocks design used in each year constituted a balanced data set with a sufficiently high number of individuals (109 RILS in common) to allow a statistical estimate of GxE compared with some of the other sites. The environmental (E) component corresponded to compounded effects of blocks and year. In the case of the global data set, there was a variable number of RILs (Table 1) for which 7 to 10 individual fiber data were collected from the 11 experiments depending on the trait measured. The year and location effects were confounded in the analysis of variance and the GxE effect was not testable. The results of the 2 analyses of variance of fiber data are presented in Table 3. In the Brazil-only analysis, all fiber traits displayed strong (P < 0.0001) genotype effects and high heritabilities (all superior or equal to 0.78). Brazilian data also indicated that half of the fiber quality measures were associated with non significant GxE (RIL × year) effects. When significant (MR, IM, ML, UHML, UI, and elongation), this interaction component was always strongly inferior to the genotypic (RIL) effect (Table 3), confirming data from the literature and indicating that the genetic influence on major fiber characteristics was generally greater than non-genetic influences (reviewed in [5]). As compared to Brazil data, it could be assumed that a larger environmental variation had been introduced from the testing over 11 sites × years; global estimation of trait heritabilities based on the complete data set resulted in slightly lower values, between 0.37 for maturity ratio and 0.69 for yellowness index (Table 3). These values were generally superior to the narrow sense heritabilities estimated from the BC _2_/BC _2_S _1 _regression [7] and fell within the range of reported values from the literature [5]. Highest heritabilities associated with a non-significant RIL × year interaction component for the Brazilian data were related to color indices (Rd and + b) and to fiber fineness.

**Table 3:** Analysis of variance of fiber traits

			**ANOVA (Brazil)**			**ANOVA (Global)**		
		
**Fiber trait category (acronym)**	**Trait**	**Symbol**	**F value (RILxYear)**	**F value (RIL)**	**h^2 ^(Brazil)**	**nr sites**	**F value (RIL)**	**h^2 ^(Global)**
		
Fineness (FIN)	Fineness	H	1.07 (NS)	26.6 (***)	0.88	9	9.8 (***)	0.57
	Standard Fineness	Hs	1.17 (NS)	13.8 (***)	0.77	7	4.3 (***)	0.38
	Maturity Ratio	MR	1.38 (*)	24.1 (***)	0.89	10	5.9 (***)	0.37
	Micronaire	IM	1.42 (*)	16.4 (***)	0.85	10	9.8 (***)	0.53
		
Length (LEN)	Mean Length	ML	1.53 (**)	20.5 (***)	0.88	8	11.3 (***)	0.64
	Length	UHML	1.51 (**)	27.9 (***)	0.91	11	13.0 (***)	0.61
Length Uniformity (UNI)	Uniformity Index	UI	1.58 (**)	13.3 (***)	0.83	7	6.8 (***)	0.49
		
Strength (STR)	Strength	STR	1.29 (NS)	20.4 (***)	0.83	10	9.7 (***)	0.54
		
Elongation (ELO)	Elongation	Elo	1.32 (*)	15.9 (***)	0.83	7	4.6 (***)	0.46
		
Color (COL)	Reflectance	Rd	1.09 (NS)	23.6 (***)	0.86	7	11.7 (***)	0.60
	Yellowness Index	b	1.12 (NS)	32.9 (***)	0.90	7	16.8 (***)	0.69

* Significant at P < 0.05, **P < 0.01, ***P < 0.001

Analysis of variance were made (i) in the 2 experiments in Brazil (Br7 and Br8), and (ii) over the complete (Global) set of 11 experimental sites. Individual broad sense heritabilities were calculated from the estimates of variance components.

Within a trait category, the strongly significant correlations between mean length, ML, and upper half mean length, UHML (r = 0.95) or between the 2 color parameters, Rd and + b (r = -0.76) are inherent to the measurement equipment and are well described [5]. Within the fiber fineness/maturity category, 3 parameters were also well correlated: H, MR and IM (r = 0.68 for H/MR, r = 0.85 for H/IM and r = 0.85 for MR/IM), while the 4^th ^parameter, standard fineness, Hs, was the least correlated with the 3 others. This can be explained by the fact that Hs is calculated as the ratio of H to MR, and is used to compare cottons of very different maturities. There was also a fairly high and positive correlation between mean fiber length and strength (mean r = 0.57), but no significant correlation between fineness and length or fineness and strength, although the 3 fiber traits, strength, length and fineness, are phenotypically associated in the 2 parents.

### QTL analyses of RIL data sets

One hundred and sixty seven significant (LOD > permutation-based threshold) QTLs were detected by composite interval mapping over the 11 RIL experiments, involving 93 individual series of data (see Additional file 1, Table S1 and summary in Table 4). The 167 significant QTLs individually explained 8.4% to 48.4% of the phenotypic variation, with LOD as high as LOD = 10.6 for color QTLs on c8. The 167 significant QTLs were a subset of the larger number of 651 putative QTLs (including all QTLs of LOD > 2) which were considered for meta-analysis.

**Table 4:** Distribution of QTLs in the RIL experiments

		Fineness	Length	Uniformity	Strength	Elongation	Color	Total
**Site**	Br7	9 (39)	2 (8)	1 (5)	0 (3)	3 (11)	5 (14)	**20 (80)**
	Br8	6 (21)	0 (4)	1 (7)	0 (4)	3 (7)	4 (12)	**14 (55)**
	Cs7	5 (21)	0 (2)	0 (0)	0 (0)	0 (0)	0 (0)	**5 (23)**
	Cs8	3 (23)	3 (16)	3 (11)	0 (5)	2 (9)	0 (0)	**11 (64)**
	Cs9	9 (20)	1 (4)	3 (7)	0 (3)	0 (4)	0 (0)	**13 (38)**
	Ga7	7 (32)	4 (12)	3 (7)	0 (5)	1 (2)	4 (16)	**19 (74)**
	Ge6	4 (14)	3 (14)	0 (0)	3 (5)	0 (0)	8 (17)	**18 (50)**
	Lu7	9 (40)	6 (26)	0 (0)	3 (14)	0 (0)	4 (9)	**22 (89)**
	Lu8	5 (21)	1 (8)	1 (6)	1 (8)	3 (4)	0 (0)	**11 (47)**
	Mp7	3 (12)	2 (9)	0 (0)	3 (8)	0 (0)	3 (18)	**11 (47)**
	Mp8	6 (27)	5 (13)	2 (6)	2 (5)	4 (12)	4 (21)	**23 (84)**

**Chromosome**	1	1 (1)	1 (2)	0 (0)	0 (2)	0 (1)	2 (5)	**4 (11)**
	2	1 (13)	0 (5)	0 (1)	0 (0)	3 (6)	0 (0)	**4 (25)**
	3	1 (5)	5 (12)	0 (1)	0 (5)	0 (1)	0 (0)	**6 (24)**
	4	3 (7)	5 (9)	0 (0)	1 (5)	0 (0)	0 (1)	**9 (22)**
	5	3 (8)	0 (10)	1 (6)	0 (1)	0 (0)	1 (3)	**5 (28)**
	6	2 (7)	1 (1)	0 (3)	0 (3)	1 (2)	5 (11)	**9 (27)**
	7	0 (5)	0 (0)	0 (0)	1 (4)	0 (0)	2 (6)	**3 (15)**
	8	0 (2)	0 (6)	0 (0)	0 (0)	0 (0)	8 (25)	**8 (33)**
	9	3 (24)	2 (8)	1 (4)	1 (5)	1 (4)	2 (6)	**10 (51)**
	10	1 (7)	0 (2)	0 (1)	0 (1)	0 (0)	0 (3)	**1 (14)**
	11	0 (5)	0 (4)	0 (0)	0 (0)	0 (0)	2 (6)	**2 (15)**
	12	6 (20)	0 (2)	7 (14)	2 (6)	2 (4)	0 (3)	**17 (49)**
	13	0 (5)	1 (3)	0 (0)	0 (1)	1 (2)	0 (0)	**2 (11)**
	14	0 (6)	1 (8)	0 (0)	1 (2)	0 (0)	1 (3)	**3 (19)**
	15	9 (27)	0 (0)	1 (4)	1 (1)	3 (10)	2 (5)	**16 (47)**
	16	4 (10)	0 (0)	1 (4)	0 (1)	0 (0)	0 (1)	**5 (16)**
	17	5 (14)	0 (1)	0 (0)	0 (0)	0 (0)	0 (1)	**5 (16)**
	18	3 (14)	0 (1)	0 (0)	0 (2)	0 (1)	1 (1)	**4 (19)**
	19	3 (10)	2 (7)	1 (3)	2 (7)	2 (7)	0 (3)	**10 (37)**
	20	2 (8)	0 (0)	0 (0)	0 (0)	0 (1)	0 (1)	**2 (10)**
	21	5 (20)	2 (9)	0 (1)	2 (8)	2 (2)	2 (8)	**13 (48)**
	22	1 (8)	0 (0)	0 (0)	0 (0)	0 (3)	1 (1)	**2 (12)**
	23	1 (4)	0 (4)	1 (2)	0 (0)	0 (0)	0 (2)	**2 (12)**
	24	2 (8)	2 (7)	0 (0)	0 (1)	0 (3)	0 (0)	**4 (19)**
	25	7 (22)	0 (5)	0 (2)	0 (3)	0 (0)	3 (12)	**10 (44)**
	26	3 (10)	5 (10)	1 (3)	1 (2)	1 (2)	0 (0)	**11 (27)**

	**Total**	**66 (270)**	**27 (116)**	**14 (49)**	**12 (60)**	**16 (49)**	**32 (107)**	**167 (651)**

Distribution of the 167 significant QTLs (LOD > permutation based threshold) and of the total 651 QTLs (including putative QTLs of LOD > 2 shown in parentheses) from the analysis of the RIL phenotypic data and shared among the 6 fiber trait categories, 11 sites and 26 chromosomes.

The number of RILs sampled and analyzed for their fiber parameters per location/year ranged from as low as 38 for Ge6 to 128 for the Br8 data set, and the number of fiber traits measured at the different sites also varied (Table 1). The lowest number of QTLs (5) was detected for Cs7 (Table 4) as only fineness components and fiber length were measured at this site because the small fiber quantities generated were insufficient for most fiber measuring instruments. For other data sets (Table 4), where more fiber parameters were measured, the number of significant QTLs per data set varied between 11 (Cs8, Lu8 and Mp7) and 23 (Mp8).

Globally, the largest group of QTLs (66) was related to the fiber fineness/maturity category as this category integrates more variables than any of the others (Table 4) and includes H, MR, Hs, and micronaire. Fiber length (UHML, ML and UQLw) and fiber length uniformity (UI, and SFI) detected 27 and 14 QTLs, respectively. Strength and elongation were accounted for by 12 and 16 QTLs, respectively, but were not detected at all sites. Fiber color, represented by 2 parameters, Rd and + b, revealed 32 QTLs in total.

The number of fiber QTLs per chromosome (Table 4) varied. The lowest numbers of QTLs were detected on c10 (1 QTL) and c11, c13, c20, c22 and c23 (2 QTLs each), and the highest numbers were detected on c12 (17 QTLs), c15 (16), c21 (13) and c19 (10). A similar number of significant QTLs were detected on chromosomes of the A _t _sub-genome (c1-c13) and the D _t _sub-genome (c14-c26), with 80 and 87 significant QTLs, respectively (Table 4).

The parental contribution (additivity) for the 167 significant QTLs was also analyzed. In a commercial context, high values of length, strength, elongation, maturity or reflectance are sought by fiber merchants and spinners, while low values of fineness and yellowness are favored. In our inter-specific cross, the *Gh *parent, Guazuncho-2 is expected to be the donor for slightly better fiber color properties as well as for fiber elongation, while the *Gb *parent, VH8-4602, is expected to be the donor for all other parameters, including the most commercially important ones, length, strength and fineness. From our QTL data, we observed that the relative contribution of the 2 parents was sometimes "as expected" with more QTLs having a positive contribution from the predicted donor parent, as was the case for fiber elongation (100%, 16/16, by the *Gh *parent), fiber color (72%, 23/32, by the *Gh *parent) and fineness (73%, 19/26, by the *Gb *parent). However, in the case of fiber strength and length the situation was reversed, as the donor parent VH8-4602 *(Gb) *contributed positively at less QTLs than Guazuncho-2 (33% for fiber strength and length, i.e. 4/12, and 9/27, respectively).

### QTL analyses of BC data sets

In total, 67 significant QTL (LOD > permutation-based threshold) were detected after the re-analysis of the 3 BC data sets, BC _1_, BC _2 _and BC _2_S _1 _(see Additional file 1, Table S1, also summarized in Additional file 2, Table S2). This is higher than the 50 significant QTLs of the original report [7], because the three generations were considered separately here, while the QTLs reported earlier were derived from pooled data. Details of these QTLs (additivity, distribution) were, however, only moderately altered as compared to the initial report [7]. Using a relaxed threshold there were 328 putative QTLs (LOD > 2) detected over the three BC generations and these were used in the meta-analysis.

### Comparative QTL analysis and primary meta-analysis

QTL analyses of fiber data from the 11 RIL experiments and 3 backcross generations altogether generated 234 significant QTLs (exceeding LOD threshold) and 979 QTLs (including putative QTLs of LOD > 2). Visual inspection indicated a moderate level of transferability between RIL and BC data sets. The highest frequency of conserved QTLs from RIL and/or BC data sets mapping at close distance were encountered for fiber color on c25 involving 8 (5 RIL and 3 BC data sets) of the 10 data sets where it was measured, for length on c4 and fineness on c12 and c15 (6 RIL data sets), for fiber color on c8 (5 RIL data sets), for fiber length on c3 (3 RIL and 3 BC data sets). These regions of conserved QTLs were only identified at the lower detection threshold (LOD2) and therefore mainly comprise putative QTLs. Conversely, some strong QTLs were found to be specifically detected in only a limited number of situations. For example, strength QTLs on c3 with strong effects were only detected in the BC populations, but none were detected in the RIL experiments. Two possible factors may account for these differences between BC and RIL: (1) population structure and heterozygosity (50% in the BC _1_, 25% in the BC _2 _and < 5% for the RILs), and (2) environmental interactions (glasshouse/field, year effect) including intrinsic measurement variability (like high CV).

In addition to QTLs from the RIL and BC data, fiber QTLs reported by [16] were also projected onto the RIL-BC consensus map. In all, the *QTLProj *module of MetaQTL was used to project over 1100 QTLs onto the consensus map, including all 328 QTLs of the BC data, all except 10 of the 651 QTLs of the RIL data and 140 of the 212 QTLs from Rong *et al. *[16]. The 10 RIL QTLs that could not be projected (6 on c4, 3 on c6 and 1 on c24) may be due to minor remaining map inconsistencies in some terminal regions between the RIL map and the consensus map.

**Figure 1:**
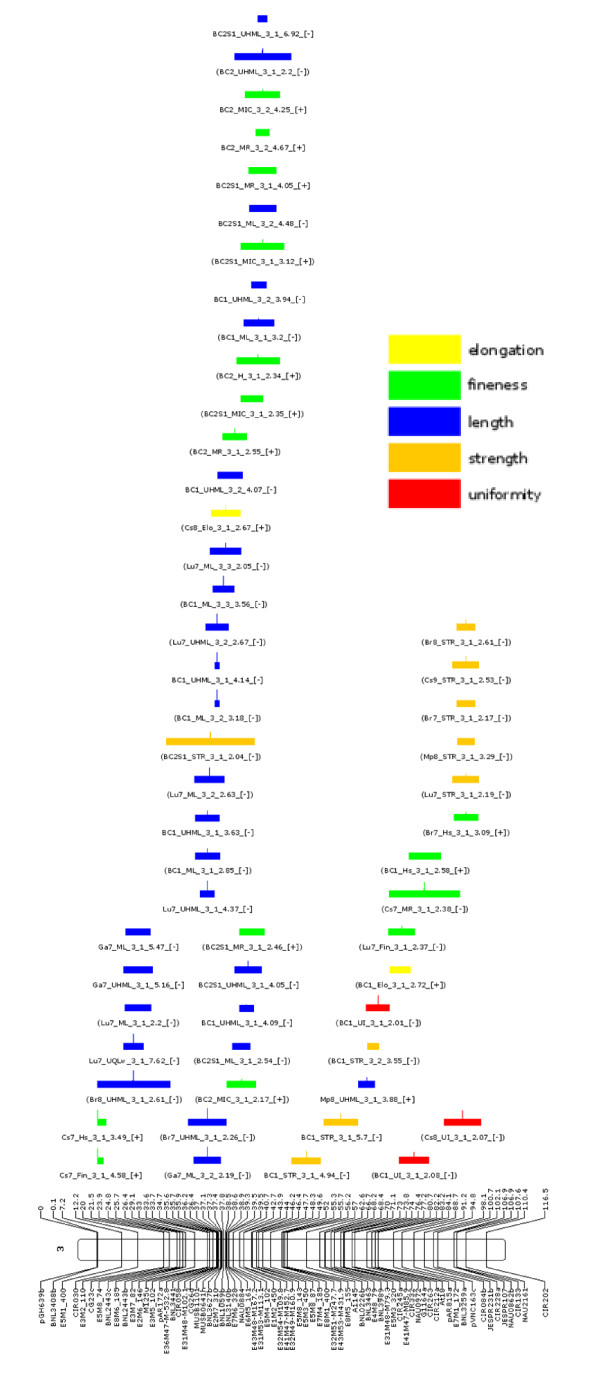
**Projections of QTLs and confidence interval positions on chromosome 3**. Projections of 55 QTLs and confidence interval for various fiber traits on chromosome 3 (Figures for other chromosomes are shown in Additional file 3, Figure S1). Positions of putative QTLs (LOD > 2) and one-LOD drop off CI, as output data from QTL analysis with WinQTL Cartographer software, are projected on the Guazuncho-2 x VH8-4602, RIL-BC-consensus map. The QTL positions are arbitrarily centered (by the software) relative to the CI (input data). QTLs originate (i) from the 11 RIL experiments reported in this paper, (ii) from the 3 backcross generations reported in Lacape *et al. *[7] and re-analyzed using the consensus map and (iii) the fiber QTLs compiled in Rong *et al. *[16] that could be projected on the Guazuncho-2 x VH8-4602 consensus map. QTL nomenclature of RIL and BC data is a concatenation of the RIL experiment location (or BC generation), trait name, chromosome, rank on chromosome, LOD peak value and sign of additivity (shown in square brackets) relative to the *Gh *(Guazuncho-2) parent. The QTL name is bracketed when its LOD value is inferior to the permutation-based (1000) threshold but superior to LOD2. Names for other QTLs were kept as in their original reference [16]. Fiber quality categories comprise fiber length (grouping UHML, ML, UQLw), length uniformity (UI and SFI), fineness (H, Hs, MR and micronaire), color (Rd and + b), strength and elongation. Bars for QTLs of a given category on a given chromosome are filled in the same color, but colors may differ from one chromosome to the next (see color legends in Additional file 3, Figure S1).

The graphical representations of the projections of fiber QTLs for the 26 chromosomes are presented in Additional file 3, Figure S1, with an example, chromosome 3, shown in Figure 1.

**Figure 2:**
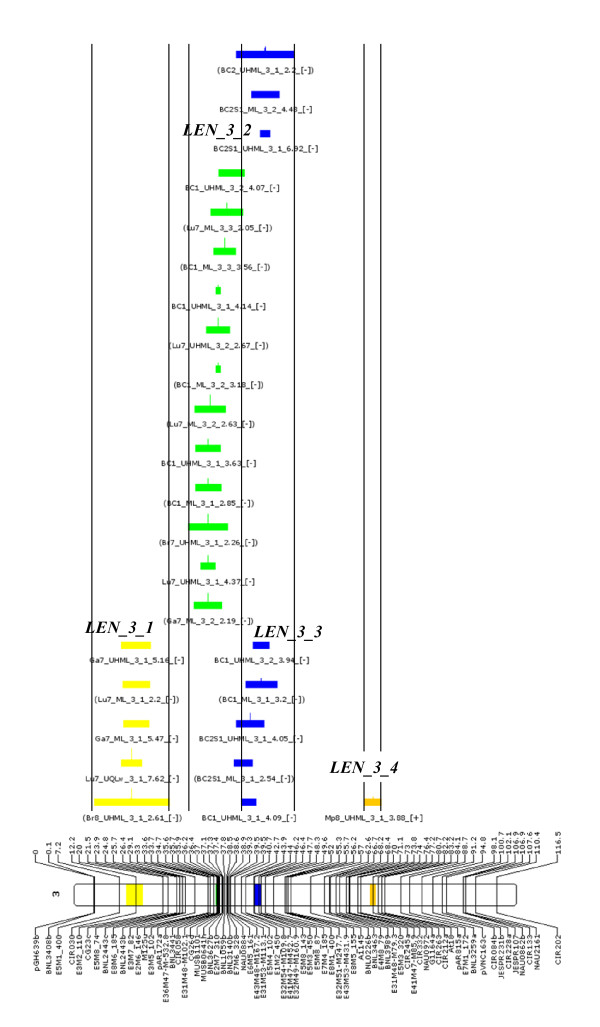
**Results of QTL clustering (meta-analysis) by MetaQTL**. Results of QTL clustering (meta-analysis) by MetaQTL of QTLs for fiber length (LEN) on chromosome 3 with 26 QTLs (those displayed in blue in Figure 1) grouped as 4 clusters. QTLs belonging to the same cluster have the same color. QTLs common to two clusters are represented with the color of each cluster, the length of the color segments being proportional to the probability for the QTL of belonging to the cluster of the same color. The vertical lines provide approximate bounds for the clusters. Figures for all 30 individual trait x chromosome combinations are shown in Additional file 5, Figure S2.

The one-LOD confidence interval (CI) was used as the primary screen to identify a limited number of chromosome regions with consistent localization for meta-analysis [39]. For a given chromosome and for a given fiber trait category, the presence of QTLs from at least 4 (most often 5) independent data sets mapping anywhere on the same chromosome was considered as the cutoff for clustering with the *QTLClust *module of MetaQTL. Thirty chromosome regions were chosen, and in each case the best clustering model based on AIC criterion was then implemented and clusters generated by MetaQTL. Nearly half (471 out of 979) of the QTLs served as input data for the meta-analysis, resulting in 135 clusters on 19 different chromosomes. The seven other chromosomes (c1, c7, c11, c13, c14, c20 and c22) did not show sufficient QTL co-localization for any fiber trait to warrant clustering. An example of the clustering output is shown in Figure 2 for the 26 QTLs related to fiber length detected on chromosome 3. Detailed clustering results are shown in Additional file 4, Table S3 and summarized in Table 5. Output figures generated by MetaQTL for all 30 trait x chromosome combinations are shown in Additional file 5, Figure S2, with corresponding text comments in Additional file 6, Table S4. Despite an acceptable level of representation among the data sets, some of the combinations listed in Table 5 and Additional file 6, Table S4, were qualified as still only indicative because there was sometimes a poor level of co-localization and consequently high numbers of putative clusters were generated by MetaQTL. We decided to be conservative with these few cases because of the possible uncertainty in the mapping data, whereby markers (and the inferred linked QTLs) could be erroneously mapped at distant locations.

**Table 5:** Summary of the combinations used for clustering by MetaQTL

								**RIL experimental site**^**$**^											**BC**^**$**^			
***Chr***	***Trait***	***Total QTLs***	***Parent***	Total clusters identified*	***Meta - cluster designation***	***Clusters coalesced into Meta - clusters***	***cM***	***Ge6***	***Cs7***	***Mp7***	***Br7***	***Ga7***	***Lu7***	***Cs8***	***Mp8***	***Lu8***	***Br8***	***Cs9***	***BC1***	***BC2***	***BC2S1***	***Confirms litera - ture***

2	Fineness	18	Gua	3(+ 1)	**FIN_2**	1 + 2 + 3	23 + 36 - 43				x	x	x		x			x		(x)	x	yes (# loc)
	Elongation	6	Gua	1	**ELO_2**	1	46	NA	NA	NA			NA	x	x		x	x				yes
3	Length	26	VH8	3(+ 1)	**LEN_3**	2 + 3	36 - 47				x	x	x		(x)		(x)		x	x	x	yes
	Strength	9	VH8	1(+ 3)	**STR_3**	4	110		NA		x		x		x		x	x	(x)		(x)	yes
	Fineness	15	VH8	3	**indicative**	3	(101)		x		x		x						x	(x)	(x)	yes (# loc)
4	Length	9	Gua	1	**LEN_4**	1	67		x	x		x	x	x	x							yes
5	Fineness	11	Gua	4	**indicative**	1 + 2 + 3	(25 - 45 - 50)			x	x				x	x		x				yes
	Length	11	Gua	3(+ 1)	**indicative**	1 + 2 + 3	(22 - 32 - 49)	x	x	x						x		x				yes
6	Color	13	Gua	3	**COL_6**	3	35 - 45 - 51		NA	x	x	x	x	NA		NA	x	NA	x		x	yes
8	Color	34	Gua	7	**COL_8A**	2 + 3 + 4	46 - 51 - 54	x	NA	x	x	x		NA	x	NA		NA				
					**COL_8B**	5 + 6 + 7 + 8	84 - 88 - 109 - 121		NA	x				NA		NA	x	NA	x		x	yes
9	Fineness	25	VH8	4(+ 3)	**FIN_9A**	1 + 2 + 3	20 - 41 - 51	x			x				x		x	x			x	
					**FIN_9B**	6 + 7	95 - 102					x	x		x							yes
	Length	8	Gua	3	**LEN_9**	1 + 2 + 3	18 - 36 - 39	x					x	x	x							yes (oppos)
10	Fineness	16	VH8	5(+ 1)	**FIN_10**	3 + 4 + 5	58 - 70 - 81			x			x	x				x	(x)		x	yes
12	Fineness	20	VH8	2(+ 4)	**FIN_12**	1 + 2	4 - 14	(x)	x		(x)	(x)	x	x	x		x	x				yes
15	Fineness	30	VH8	6	**FIN_15**	2 + 3 + 4 + 5	62 - 67 - 73 - 84		x		x	x	x	(x)		x	x	x		(x)	(x)	yes
	Elongation	10	Gua	2(+ 2)	**ELO_15**	2 + 3	68 - 72	NA	NA	NA	x	x	NA	x			x				(x)	yes
16	Fineness	10	VH8	6	**indicative**	1 + 2 + 3	(41 - 50 - 64)		(x)		x	(x)	x	(x)	x							yes
17	Fineness	14	VH8	2	**FIN_17**	1 + 2	18 - 28		x	x	x	x				x	x					yes
18	Fineness	17	VH8	5	**FIN_18**	3	49		x	x		x	(x)		x	(x)		x	x		(x)	yes (oppos)
19	Elongation	14	Gua	5	**indicative**	1 + 2 + 3/4 + 5†	(83 - 148)	NA	NA	NA	x	x	NA				x		x	x	x	yes
	Length	15	Gua	5	**indicative**	2 + 3 + 4	(64 - 101)	x					x		x				x		x	yes (oppos)
	Fineness	18	Gua	7	**indicative**	2 + 3 + 4 + 5/6 + 7†	(51 - 200)	x		x			x	x	x	x			x	x		yes (oppos)
21	Strength	7	VH8	2(+ 2)	**STR_21**	2 + 3	75 - 80	(x)	NA	(x)			(x)	x		x		x				yes
	Fineness	23	VH8	7 + 1	**FIN_21A**	2 + 3 + 4 + 5	65 - 73 - 81 - 93		x		x		x		x			x		x	x	yes
					**FIN_21B**	6 + 7 + 8	129 - 149 - 163				x		x	x		x	x			x	x	yes
23	Length	10	VH8	2 + 1	**LEN_23**	2 + 3	98 - 117					x				(x)	x		x	x	x	yes (# loc)
	Strength	9	VH8	2(+ 2)	**indicative**	3 + 4	(86 - 102)		NA										x	x	x	yes
24	Length	7	VH8	1(+ 1)	**LEN_24**	1 + 2	67 - 75	x			x			x				x				yes (# loc)
25	Color	25	Gua	5(+ 1)	**COL_25A**	1 + 2 + 3	46 - 57 - 66		NA	x	x	x		NA		NA	x	NA	x	x	x	yes
					**COL_25B**	4 + 5	80 - 84		NA	x	x	x	x	NA		NA		NA		x		
	Fineness	25	VH8	5	**FIN_25**	2	48 - 60		x			(x)		x	x		x	x		x		yes
26	Length	16	Gua	5	**indicative**	1 + 2/3/4 + 5†	(8 to 94)	x					x	x	x					x	x	yes (oppos)

* the 1st number represents the number of "reliable" clusters supported by QTLs from multiple experimental sites. Additional clusters represented by isolated QTLs are shown in parentheses

^$ ^x, indicates that meta-cluster was supported by QTL detected at this experimental site, (x) that isolated QTLs was observed, NA that the trait was not measured

† the slash separates groups of clusters suspected to delineate different indicative meta-clusters

Summary of the 30 trait x chromosome combinations used for clustering by MetaQTL software and of 25 highly supported meta-clusters. Total of QTLs (LOD>2) detected, shared direction of additivity (toward Guazuncho-2 (GUA), *G. hirsutum*, or VH8-4602 (VH8), *G. barbadense*, parent), number of clusters in best clustering model as proposed by MetaQTL (following Akaike-information criterion), names and positions of highly supported clusters and meta-clusters, and indication for an occurrence of at least one congruence (same region and same directionality *Gh/Gb*) with QTL reported in the literature. Meta-cluster designation (trait acronym and chromosome) is suffixed with a letter (A, B) when several meta-cluster are mapped on the same chromosome.

### Prioritization among clusters and proposals for meta-clusters

The QTL meta-analysis relied strongly upon the fact that the CI of proposed co-localizing LOD peaks were overlapping. We used the one-LOD drop off method for calculation of CI. Considering the inherent limits related to the notion of CI (peak height, population size and structure) and the possible uncertainties in the locus order and distances in genetic maps, we also tested an alternative, formula-based method for CI calculation [40]. All clustering analyses were re-run with MetaQTL and clustering results with new formula-based CI were found to differ. The much larger individual CI with the formula-based method (see Additional file 1, Table S1) most often resulted in a lower number of clusters that also had larger CI (not shown). Apart from the effect of the method of calculation of the CI, it is also expected that mapping errors and uncertainties in mapping will have a strong effect on LOD peak and CI positioning. For these reasons, we believe that in some cases the identification of separate clusters by MetaQTL software could be questioned. In several instances, we have proposed that multiple clusters mapping at fairly close distances and containing individual QTL for which CI essentially overlapped and with the condition that their individual additivity agreed, be coalesced and collectively referred-to as candidate "meta-clusters" (Table 5 and see comments in Additional file 6, Table S4). Most prominent examples of such meta-clusters were found for fiber color on c8 and c25, length on c24, fineness on c2, c12, c15, and c17 or for strength on c23. Applying a prioritization among clusters and meta-clusters we identified a total of 25 robust cases (6 corresponded to a single cluster, all others to a meta-cluster grouping 2 to 4 clusters), listed in Table 5 and Additional file 6, Table S4, and mapped to 15 different chromosomes, shared between 11 for fiber fineness, 5 for length, 2 for strength, 5 for color and 2 for elongation. The higher representation of fiber fineness as compared to other categories may reflect the inherent complex nature of fiber fineness as it includes two fairly independent interacting components (fiber diameter and fiber cell wall thickness or maturity) that are not separated by the measurement devices used. Regarding fiber strength, only 12 significant QTLs (Table 4) and 2 clusters (Table 5), on c3 and c21, were detected. An issue here may be in the reliability of the HVI-based measurement of fiber strength within the range of values that occurred in the RILs. Considering that micronaire reading is used by the HVI to estimate fiber plug mass and convert breakage force to g/tex (strength unit), it might be important to bear in mind that the parent VH8-4602 and a number of RILs had an extremely low micronaire reading (<2.0) and this could cause a biased estimation of bundle strength.

In the majority of cases (22 out of the 25 robust clusters and meta-clusters) the directionality of the additive effect followed the expectation in relation to the parental phenotypes (Table 5): improved trait values were contributed by the *Gh *parent for the 5 fiber color and 2 fiber elongation meta-clusters and by the *Gb *parent for 2 fiber strength meta-clusters, for 10 of the 11 fiber fineness clusters and for 3 of the 5 length meta-clusters. A positive contribution by the inferior parent was also reported in the case of fiber length in [13] for 61% (17 out of 28) of their non over-lapping fiber length QTLs. This effect may explain the marked occurrence of transgressive segregation.

From these 25 robust cases, 16 may be considered of higher significance based on additional consistency evidence (Table 5) both from our data and data from the literature:

- 2 clusters or meta-clusters for fiber length on c3 and c4

- 9 clusters or meta-clusters for fiber fineness on c9 (2×) and c21 (2×) and on c12, c15, c17, c18 and c25,

- 5 clusters or meta-clusters for fiber color on c8 (2×) and of c25 (2×) and c6.

## Discussion

The two *Gossypium *species used in this study represented highly divergent genotypes and were purposely selected as parents for hybridization to both maximize the chances of detecting significant QTLs for all fiber traits and to produce material that might have value as a source of variation for breeding. Because of their interspecific origin, some of the RILs showed reduced fertility and productivity resulting in the poor representation of data from some lines and sites (Table 1). Another contributing factor for the poor performance of the RILs could be that most of the experimental sites were outside of South-Central America, the area of adaptation of the two parents.

The averaged fiber characteristics of the RILs were always intermediate between the 2 parents, but closer to the *Gh *parent than to the *Gb *parent (Table 2). This is in accordance with the observed distorted allelic constitution of most of the RILs, that contained on average 71% and 29% of alleles of *Gh *and *Gb*, respectively [41]. Some individual RILs displayed fiber characteristics outside of the parental range (transgressive phenotypes) and may thereby provide useful material to include in breeding crosses. Interestingly, the parental allelic composition of the RILs, varying between 95/5 to 32/68 parental (*Gh%*/*Gb%*) allelic content, showed no correlation with fiber quality: the best performing RILs were not those having the highest allelic content in VH8-4602 alleles.

### Complex QTL networks determine cotton fiber quality

The quantitative variation in the fiber characteristics observed over 11 (year × site) RIL experiments did convert into a high number of QTLs. Including the 167 significant fiber QTLs from the RILs, ca 600 fiber QTLs have been collectively reported so far from 9 different inter-specific *Gh *× *Gb *populations. Although we have reported some stability in QTL detection among this high number of fiber QTLs, the overall complex picture behind fiber QTL mapping may be put into perspective with the fact that fiber quality is assessed by a series of fairly independent physical measurements, essentially related to dimensional features like diameter, thickness and length. The complex genetic network is also consistent with biological evidence that cotton fiber development involves many genes (probably over 90% of all cotton genes are expressed in cotton fiber, [42]) and gene interactions.

### Meta-analysis shows (moderate) clustering

Although earlier published comparative QTL mapping studies concluded that there was a poor level of transferability of QTLs between populations [7,16], it was expected that at least the strongest fiber QTLs would be confirmed, thus hopefully reducing the number of relevant QTLs [43] to a more manageable level from a breeder's perspective. The four populations studied here (3 backcross-derived and 1 RIL) only revealed a moderate level of convergence across data sets. Regarding the RIL experiments, the differences in the QTLs detected among the 11 sites probably resulted from the low number of individual RILs examined in some RIL experiments. The most important limiting factor causing reduced accuracy of detection of QTLs probably relates to the range of different population sizes used in the current study [22,44] rather than to the effect of environment, because heritabilities of fiber traits were shown to be medium to high. Another factor negatively affecting QTL detection power was the distorted genomic composition of the RIL population.

**Figure 3:**
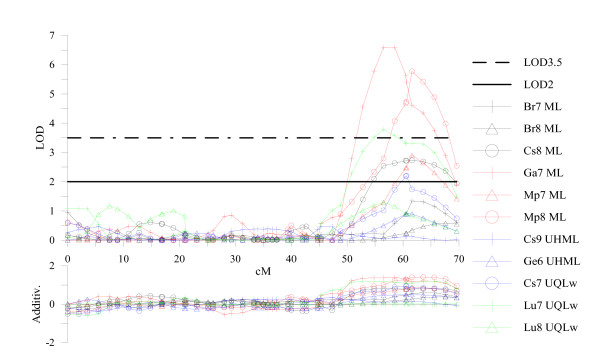
**Variation of the LOD score profiles along chromosome 4 for fiber length measurements**. Graphical representation of the variation among 11 data sets of the LOD score profiles (upper panel) and of the observed additivity effect (in mm) as conferred by the *Gh *parent (lower panel) along chromosome 4 for fiber length measurements (either HVI-based, as Upper Half Mean Length, UHML or Mean Length, ML, or AFIS-based, as Upper Quartile Length per weight, UQLw). Phenotypic data were obtained from 11 experiments of the RIL population and analyzed with WinQTL Cartographer by composite interval mapping (loci positioned on the RIL map). The 2 horizontal lines correspond to 2 different LOD thresholds: the permutation-based (1000 permutations) threshold averaged from the 11 sets, ie LOD = 3.5 (dotted line), and an arbitrary value of LOD = 2 (solid line).

For these reasons, a fairly low detection threshold (LOD2) was used to declare LOD peaks as putative QTLs for use in across-experiment comparisons. Previous experience in QTL interval mapping has indicated that it is common for "background" noise in LOD score profiles to occur in the range of LOD values between 0 and 1. Figure 3 provides an example of the usefulness of lowering the LOD detection threshold for QTL detection in the particular context of such across-experiment comparisons. In the case of the 11 independent series of fiber length measurements and their LOD profiles along chromosome 4, only 3 significant QTLs having a LOD superior to the permutation-based threshold (3.5 in this case) would have been declared in a bottom region of c4. However, when a lower LOD threshold is used (LOD2), 3 additional "putative" QTLs (6 instead of 3) would be integrated in the meta-analysis. In this particular case, the reduction of the LOD detection threshold to LOD1 would even have allowed 2 additional locations to be included (i.e. 9 out of the total 11). Lowering detection thresholds also had some deleterious effects and probably resulted in significant inflation of QTL numbers, particularly by splitting broader peaks into multiple peaks in close proximity, as well as the probable inclusion of some false positives.

### Consistency with previously reported fiber QTLs

The BC and RIL populations included in meta-QTL analysis had the same *Gh *and *Gb *parents (Guazuncho-2 and VH8-4602). When possible, we compared our results with fiber QTLs reported from different crosses in the literature, for which the map locations on our BC-RIL consensus map could be extrapolated. Interestingly, for at least 22 of the 25 prioritized clusters and meta-clusters, additional support was provided by the reported localization on the same chromosome of at least one QTL from the literature, of which 17 also agreed in their directionality (Table 5). The cases of contradiction in directionality (see examples of fiber length on c9 or fineness on c18) may relate to real differential allelic effects as the parents differed. In a few cases, QTLs reported in the literature were not detected in this study. The strong fiber strength QTL reported from cross TM1 x 7235 by Zhang *et al. *[45] localized on c24/D8, near locus BNL2961, and presumably originating from the *G. anomalum *and *G. barbadense *lineages of one of the 2 parents, was only corroborated by one putative QTL, *Lu8_STR *with peak LOD = 2.54 and higher strength by the *Gb *alleles. A recent intraspecific RIL population [46] also reported 13 fiber QTLs, including a region of co-localization for 5 QTLs for different fiber traits on c7, but none was corroborated by any of our clusters. Similarly, the central region of c6 containing the gene *t1 *governing leaf hairiness did not contain any confirmed fiber QTL from our data, although leaf hairiness has been correlated with fiber quality in 2 segregating intra-*G. hirsutum *populations [47]. The only putative case of co-localization (distant by only 10 cM) with *t1 *was for fiber fineness QTLs detected in 2 RIL data sets, Br7 and Cs8 (not considered for clustering) co-localized with a fineness QTL from the literature, *FF06.1 *from Paterson *et al. *[6] (see Additional file 5, Figure S2). It should also be noted that several fiber QTL reports from the literature could not be considered because of lack of bridge markers and uncertain chromosome assignation; this was the case for most QTLs from 2 interspecific *Gh *× *Gb *populations [10,15], and more importantly for the majority of fiber QTLs reported from intraspecific *G. hirsutum *crosses [48,49].

### QTL coincidences for different fiber traits

The coincidence of QTLs for different fiber traits in the same genomic region was reported earlier [7,16,46]. Our data based on a larger set of QTLs confirmed this observation. In several instances a close co-localization of individual QTLs for different types of fiber traits was observed. Moreover, in the majority of cases the respective directionality of the groups of QTLs agreed with the known phenotypic association in the parents. This may be interpreted in terms of pleiotropy because of the known correlations between fiber parameters, their physical definition or their particular measurement method. Although pleiotropic genetic effects may not be overlooked, we would also consider linkage as an explanation for these cases of co-localizations.

### Subgenome distribution and homoeologous relationships of fiber QTLs

The overall distribution of QTLs and QTL clusters was not homogenous between chromosomes (Table 4 for the RILs, additional file 2, Table S2, for the BCs, and Table 5 for QTL clusters). Although, simply counting numbers of QTLs may be misleading, because of correlations between traits under consideration, and possible mis-identification of multiple LOD peaks in close vicinity (5-10 cM) as separate QTLs, it is noteworthy that chromosome 19 consistently harbored a higher number of QTLs than any other chromosome (this study, BC data [7] and [16]). The enrichment in fiber QTLs on c19 was more frequently correlated with a *Gh *additive contribution to fiber traits: higher elongation, length, and strength and lower fineness.

Subgenome distribution was equivalent between chromosomes c1-c13 of the A _t _sub-genome and c14-c26 of the D _t _sub-genome, with 105 and 129 significant QTLs (among 234 from all RIL and BC), and 13 and 12 robust meta-clusters respectively (Table 5). The extant wild D-genome diploids, including the modern species *G. raimondii*, recognized as the closest to the D-genome ancestor of polyploid cotton, only have very short and coarse non-spinnable trichomes on their seed. Thus, the finding that the D _t_-genome of tetraploid A _t_D _t _cotton contributed at least half of the QTLs (eg, alleles and genes) involved in fiber quality raises the possibility that homoeologous fiber-related genes have differentially evolved following polyploid formation [16,50]. Recent reports tend to confirm an unequal homoeologous gene expression pattern in allopolyploid cottons [51,52] leading to a differential expression of duplicated genes during fiber development [53] (1500 A _t_/D _t _couples of genes studied).

Meta-clusters generally mapped at non-homoeologous A _t_/D _t _locations. The only possible homoeologies for QTL-enriched regions with same directionality included two regions, though only indicative, for fineness along the upper half of pair c5/c19 already identified by Rong *et al. *[16], and two new potentially homoeologous regions (close to the duplicated locus BNL1440) along central regions of pair c6/c25 both mapping fiber color QTLs (Table 5, and additional files 3 and 5, Figures S1 and S2 respectively).

### Comparison of fiber QTL data with fiber transcriptomes

The chromosomal regions enriched in fiber QTLs were compared with the chromosome regions statistically enriched in fiber-expressed genes reported by Xu *et al. *[54]. These authors used available mapping data for cotton EST Unigenes from diverse libraries to assess their distribution among chromosomes. They identified a limited number of gene-rich regions: two regions at the top and the center of c5, a central region in each of c10 and c14, and a top region on c15. Cross comparison of these regions with our clusters for fiber QTLs indicated only 2 cases of putative convergence. A region at the top of c5 (0-30 cM interval on our consensus map) was over-represented in fiber "initiation" genes [54] and enriched in fiber length and fiber fineness QTLs (Table 5). Both fiber traits were consistent for a *Gh *positive effect (lower fineness and higher length), but the clustering was unreliable. Secondly, a central region of c10 (50-80 cM interval) was enriched in "elongation" genes [54] and possibly corresponded with a meta-cluster region for fiber fineness, *FIN_10 *(Table 5) consistent for a *Gb *positive effect (lower fineness). It should be borne in mind that genes classified as "expressed in cotton fibers", as in [54], may not necessarily match with the genes that underlie the fiber QTLs reported here. The subset of genes with known differential expression patterns between *Gh *and *Gb *may be more likely candidates for co-localization with fiber QTL. In this context, two recently published studies specifically focused on differences between *Gh *and *Gb *at the transcriptional level using microarrays. Alabady *et al. *[55] showed that on average 14.5% of the 12000 fiber genes profiled were specifically and differentially regulated between Pima (*Gb*) and TM1 (*Gh*) developing fibers. In another study, Al-Ghazi *et al. *[56] showed only few *Gh*/*Gb *differences in gene expression except at early stage of fiber development (20% of 24000 genes differentially expressed at 7 dpa) as compared to later stages (< 4%).

## Conclusions

A challenging issue for bridging the gap between QTL mapping and identification of the underlying causative DNA polymorphisms is the low resolution associated with QTL mapping. Over the past years QTL mapping has resulted in the identification of thousands of chromosomal regions predicted to be involved in many complex traits. However, only in a few examples has it been possible to clone the genes that underlie the traits [57]. Although our results on cotton fiber can hardly support the optimistic assumption that "QTL are accurate" [58], we have shown that the reliability of QTL-calls and the estimated trait impact can be improved by integrating more replicates into the analysis. It should, however, be emphasized that segregating populations of larger sizes than the ones that have so far been reported (majority in the range of 80-150), including our own RILs, will be needed in order to improve the detection power of QTLs [22,23], and this is particularly true in the context of phenotypic traits of complex inheritance like cotton fiber quality. In cotton, particularly with the inter-specific crosses that are needed to access the higher levels of DNA polymorphism that occurs between *Gossypium *species, this is sometimes difficult to achieve. The 140 RILs analyzed here were the end result of SSD from over 600 original F _2 _plants with large numbers dropping out at each generation due to low fertility or extremely late flowering. Meta-analysis of QTLs was shown to be useful for identifying robust QTLs, but it is not a substitute for large population sizes. It will be important in the future, that new fiber QTLs plotted on new maps sharing common markers with our consensus map be verified for their agreement with the regions of convergence identified here.

In term of practical applications, the relatively small number of candidate regions hosting fiber QTL meta-clusters that were identified here will facilitate molecular breeding strategies like marker-assisted backcrossing.

On the other hand, different avenues may also be followed to identify the genes underlying strongest QTLs. Positional cloning of QTLs has been the major route in plant QTL dissection [57], and examples in the literature are increasing, although practical interest for really complex traits remains to be shown. In addition to classical phenotypic QTL information from segregating populations, QTLs may also be resolved through association mapping of targeted candidate genes, based on an assumption that the allelic polymorphism of the gene is associated with the variation of the trait and provided that linkage disequilibrium is sufficient [59]. Alternatively, the use of segregating populations for expression QTL (eQTL) mapping and the correlative observation of the variation of transcript abundance with the variation of the phenotypic traits [60] can also provide a complementary way to resolve complexity of phenotypic QTLs. This approach using a segregating population and combining genetics (QTL mapping) and genomics (gene expression profiling), also referred as genetical genomics [61], is underway using the same RIL population [62]. Finally, the dissection of some of these specific regions, could proceed by determining the physical position of associated markers and by linking it with synteny-based information for example from *Arabidopsis *as was attempted by Rong *et al. *[16], at least until the cotton genome is sequenced.

## Methods

### Material

A recombinant inbred line (RIL) population was created by CIRAD in Montpellier glasshouses from a cross between the 2 parents, Guazuncho-2 and VH8-4602 [41]. Guazuncho-2 and VH8-4602 are typical representatives of *G. hirsutum *and *G. barbadense *(frequently referred to as Upland and Extra Long Staple (ELS) cottons, respectively). The *Gb *parent, VH8-4602, belongs to the group of Sea Island cottons among which it represents the best accession present in the extensive germplasm collection of CIRAD. VH8-4602 combines high values in both fiber length and strength compared to other *Gb *accessions. Conversely, the choice of Guazuncho-2, as *Gh *parent, was based upon its overall good agronomic behavior for the target regions of CIRAD's cotton improvement program and is associated with medium quality fibers. The same *Gh *and *Gb *parents also served to develop a backcross BC _1 _mapping population [63] and later BC _1_, BC _2 _and BC _2_S _1 _generations that were used for QTL mapping of fiber quality parameters [7] and leaf pubescence [64]. The RIL population comprised 140 lines in an F _6 _to F _9 _(6 F _6_, 19 F _7_, 89 F _8 _and 26 F _9_) stage of selfing through single seed descent (SSD), and was used to build an SSR-AFLP genetic map [40]. A BC-RIL, *Gh *× *Gb*, consensus map was constructed after integration of RIL (140 individuals) with BC _1 _(75 individuals) marker data [41]. The consensus map contained 1745 loci and spanned 3637 cM. This consensus map contained a high proportion of markers in common with other published genetic maps and was used as a reference for the overall QTL projections.

Small amounts of seeds of the original RIL population were distributed in 2006 to CSIRO, Bayer CS, and the Brazilian Agricultural Research Corporation (EMBRAPA) for seed increase under glasshouse conditions in Canberra (Australia), Ghent (Belgium), and Campina Grande (Brazil), respectively. Two of these populations of glasshouse-grown plant were used for fiber property measurements and constituted data sets Ge6 (Ghent) and Cs7 (Canberra). Two other glasshouse experiments consisted of pooled fiber samples in Montpellier, one from an environment-controlled glasshouse (set Mp7) and a second from the combination of two consecutive summer harvest in an un-controlled glasshouse (set Mp8). Mp7 fiber samples were pooled from the harvest of the last 3 generations of repeated selfing of RILs during SSD. Mp8 fiber samples originated from a randomized experiment comparing 130 RILs in 2 replications (1 pot as replicate and 2 plants per pot). Field experiments were conducted during growing seasons 2007/2008 and 2008/2009 by CSIRO in Australia (location Narrabri, NSW: data sets Cs8 and Cs9), by Bayer in the USA (2 locations in Texas, Lubbock and Idalou: data sets Lu7 and Lu8), by EMBRAPA in Brazil (location Itatuba, Paraiba: data sets Br7 and Br8) and only in 2007 by the Institute of Agricultural Research for Development (IRAD) in Cameroon (location Garoua: data set Ga7). RIL experimental details by location are given in Table 1. Except for the RIL experiments in the earliest seasons (Ge6, Cs7, and Ga7), the seeds for other RIL experiments originated from the bulked harvest from the previous years experiment, i.e. the generations of selfing may therefore differ between experiments. However the original RIL material, in an F _8 _generation of selfing, was 95% homozygous as a mean over all loci in the 140 RILs [41].

Only subsets of the 140 RILs were tested and evaluated for fiber characteristics in the various experiments (Table 1). This was a consequence, for some of the RILs, of a poor germination ability, a long duration of the life cycle (for example some late flowering RILs matured too late to be harvested in field experiments in some locations) and more importantly from low fertility resulting in insufficient quantities of fiber for testing in some fiber analysis instruments. The most comprehensive data sets were obtained from the testing in Brazil under drip-irrigation conditions during 2 growing seasons which generated data from 123 and 128 RILs respectively. In both years, 2 repetitions of the RILs were grown, and fiber quality data (Br7 and Br8) are the average of the 2 replicates. Some other sites also had replications, for which either a single pooled fiber sample per RIL was analyzed (Mp8, Lu7, Lu8), or replicates were averaged after analysis (Cs8 and Cs9).

### Fiber traits studied

Ginning of the seed-cotton was conducted locally at each site using in most cases a roller gin (or saw gin for Cs8 and Cs9 or hand ginned for Cs7) and fiber measurements were determined by each partner; by CIRAD in Montpellier (data sets Mp7, Mp8, Br7, Br8 and Ga7), by CSIRO in Australia (Cs7, Cs8 and Cs9) and by Bayer in Belgium (Ge6) and in the USA (Lu7 and Lu8). Instruments used were either High Volume Instruments, HVI Classing (Uster Technologies, Charlotte, NC), for measuring length, short fiber index, strength, elongation, micronaire, and color, or AFIS Pro systems (Uster Technologies, Charlotte, NC), for measuring length and fineness components. In addition, some specialized instruments were used to measure micronaire only (Fibronaire, Motion Control, Inc., Dallas, TX) or micronaire, maturity and fineness (Shirley maturimeter FMT, Shirley Developments, Stockport, England) (Table 1). All instruments were calibrated using international calibration cotton standards. Among the more than 20 measurements provided by the different instruments used, 13 truly non-synonymous parameters were finally taken into consideration in the analysis and were grouped into 6 broad categories, fineness/maturity further referred as fineness, length, length uniformity, strength, elongation and color.

Four individual parameters refer to "fineness" (symbolized FIN) as a category: fineness (linear density, noted H, mass per 1000 meters of fibers expressed in mtex, a tex being the weight in grams of 1000 meters of fiber), maturity ratio (noted MR, a measurement of the relative amount of the cellulose in the fiber cross-section, dimensionless; also measured as % mature fibers, PM), standard fineness (noted Hs, ratio of H to MR or mass per 1000 meters of fibers having a MR of 1, expressed in mtex), and micronaire reading (a commercial index, here noted IM, varying from 2 to 5, and based on the measurement of an air flow that passes through a porous plug of cotton fibers). Unlike fiber maturity (thickness of the fiber wall) and fiber fineness (linear density of the fiber), both of which are straightforward in interpretation (high maturity and low fineness being favorable), the interpretation of the micronaire is more complex. A given micronaire value may be reached by very different combinations of fineness (H) and maturity (MR). Two cottons, for example, one with immature (MR = 0.67) and coarse (H = 220 mtex) fibers and the other with mature (MR = 1.04) and fine (H = 150 mtex) fibers can have the same micronaire reading of 4.1. Despite its limitations, micronaire reading is still a widely used measure of a fibers' fitness for spinning applications and remains a key parameter in both the breeding and marketing of cotton.

Five variables relate to the fiber "length" category (LEN). Three parameters are direct estimates of the length, in mm or inches, of the fibers: mean length (noted ML, mean length by number of the fibers), upper half mean length (noted UHML, average length by number of the longer half of the fibers), both measured on HVI, and upper quartile length per weight (noted UQLw, length of the 25% by weight of longest fibers) measured on an AFIS instrument. In addition to these 3 length measures, 2 additional parameters represent the homogeneity of the length, or "length uniformity" distribution (UNI): uniformity index (noted UI, calculated from HVI data as the ratio between ML and UHML) and short fiber content or short fiber index (noted SFI, calculated from AFIS or HVI data, as the percentage of short fibers of less than 0.5 inches). Fiber strength (STR), the force required to break a bundle of fibers (in g/tex), and fiber elongation (ELO) measured from the same bundle of fibers as for strength and representing the degree of elasticity before breakage (dimensionless), are both measured on HVI. Finally, 2 negatively correlated parameters measured on HVI represent, in combination, the color grade of the fiber (COL): reflectance (noted Rd, relative whiteness of reflected light, in %) and yellowness index (noted + b, degree of yellowness of reflected light using a yellow filter, dimensionless). CSIRO breeders do not use color evaluations in their selection program, so this parameter was not measured in data sets Cs7, Cs8 and Cs9.

### Analysis of fiber data

The analyses of variance were conducted using the GLM procedure of the SAS software package (SAS Institute Inc., Cary, NC) for 2 series of fiber phenotypic data: - for the 2 Brazilian data sets (2 trials in 2007 and 2008 both under 2 completely randomized replicates design) using the fiber data from the 109 RIL in common between the 2 years, and - for the 11 (year × site combinations) RIL data sets. The effect of genotypes (RILs) was tested either against global residual or against genotype x set interaction when significant. Variance components were calculated using the VarComp procedure of SAS, declaring the variables Year, and replicates in the case of Brazil data, or Sites (year × site) in the global analysis, as fixed. Broad sense individual heritabilities (h²) were calculated as the ratio between genotypic (RIL) and phenotypic (error) variances.

The frequency distribution for most fiber traits fitted a normal distribution (not shown) and no data transformation was made before QTL analysis.

### QTL analyses of RIL data

A genetic map of the RIL population was published earlier [41]. The map was based on the segregation in over 140 RILs of 800 AFLP and SSR markers, and spanned 2044 cM. A subset of 656 loci separated by more than 1 cM was used for QTL analysis of RIL fiber data. We have used the classical nomenclature system for numbering the chromosomes, c1-c13 and c14-c26 for the chromosomes of the A _t _and D _t _sub-genomes respectively, as this facilitated across-experiments comparisons. The assignation of the 13 homoeologous A/D pairs is as follows: - c1/c15 (A1/D1), c2/c14 (A2/D2), c3/c17 (A3/D3), c4/c22 (A4/D4), c5/c19 (A5/D5), c6/c25 (A6/D6), c7/c16 (A7/D7), c8/c24 (A8/D8), c9/c23 (A9/D9), c10/c20 (A10/D10), c11/c21 (A11/D11), c12/c26 (A12/D12) and c13/c18 (A13/D13).

QTL were analyzed separately for each individual data set (site × year) and 5 to 12 fiber traits per data set, using WinQTL Cartographer 2.5 [65]. For each variable, interval mapping over the whole genome using multiple regression of phenotypic data on marker genotypic data was run with 1000 permutations to identify the minimum significant LOD (global risk of 5%) threshold score to be considered. Composite interval mapping (CIM) was performed with Model 6 using the markers pre-selected by stepwise regression as cofactors. Permutation LOD thresholds varied between 3.2 for UHML in the Lu8 data set and 4.6 for SFI in Cs8 data set (not reported). However, to facilitate comparisons across data sets, we also considered a relaxed LOD value (2.0) to locate additional QTLs and declare the presence of putative QTLs used in the meta-analysis (see Discussion). All QTLs (LOD > 2) were automatically localized using WinQTL Cartographer with the following parameters: - minimal space between peaks = 5 cM, and minimum LOD from top to valley = 1. The position of peaks and their one-LOD CI, corresponding to a 1 LOD drop off from the peak [39], were recovered as outputs from WinQTL Cartographer.

Individual RIL QTLs were named in the form "*Loc_Trait_Chr_X_Peak_[Add]*", for the combination of a given data set location ("*Loc*") and trait ("*Trait*"), on a chromosome ("*Chr*") with a rank (from the top to the bottom) on it ("*X*" from 1 to N if N peaks surpassed LOD2 on the given chromosome), with a LOD peak value ("*Peak*") and an additive effect ("*[Add]*", either [+] or [-]) as conferred by parent *Gh*). For example "*Ga7_UHML_3_2_3.45_*[+]" designated the presence of a QTL detected in the Ga7 data set for trait UHML, on chromosome 3, in 2^nd ^position, with a LOD value of 3.45 and a positive additive effect on trait (higher fiber length) conferred by alleles of the *Gh *parent, Guazuncho-2.

### QTL analyses of BC data

The fiber data of the 3 backcross generations derived from the same cross, Guazuncho-2 x VH8-4602 [7], were reanalyzed with WinQTL Cartographer for QTL mapping using the same procedure as for the RILs. The 3 generations consisted of 75 BC _1_, 200 BC _2 _and 200 of their selfed BC _2 _S _1 _progenies. BC _1 _and BC _2 _were grown in Montpellier glasshouses for fiber production, while the per-row value of BC _2 _S _1 _was assessed from a field experiment in Brazil as previously described [7]. Interval mapping procedures (IM and CIM) were based upon the most recent mapping data using the BC-RIL consensus map [41]. This map was considered as more reliable than the BC _1 _map initially used [7]. QTLs of BC data sets were named in a similar way as the RIL QTLs (see above), except that "*Loc*" designated either of the 3 generations analyzed, BC _1_, BC _2 _or BC _2 _S _1_.

### QTL meta-analysis with MetaQTL

Different numbers of RILs were tested in the 11 experiments (Table 1). Because of the existence of many missing phenotypic data in our RIL-by-environment data matrix, it was not possible to assess GxE interactions, except for the Brazil data, nor apply QTL-by-environment interaction (QEI) analysis models [66]. In contrast to genotypic data, existence of missing phenotypic data, as in our case, would have resulted in discarding many individuals lacking data, sacrificing all other phenotypic and genotypic information available for those individuals. However fiber quality traits being considered as heritable and GxE usually moderate [5], we have chosen to place emphasis on a comparative mapping approach, i.e. on the coincident detection on some chromosomal regions of QTLs in several year-site combinations. Fiber QTL data from the BC and RIL data sets were integrated using the BC-RIL consensus map [41] on which all QTLs were located (directly for the BC QTLs and by interpolated projection for the RIL QTLs).

Rong *et al. *[16] positioned a total of 212 fiber QTLs originating from 5 different inter-specific *Gh *× *Gb *populations, 4 F _2 _and 1 BC [6,8,11-13,16], all projected onto the *Gh *race palmeri × K101-F _2 _reference map [67]. The basic information for these QTLs including their names and CI were downloaded from http://www.plantgenome.uga.edu/cottonmap.htm. The projection of these QTLs onto the Guazuncho-2 x VH8-4602-consensus map was possible thanks to the existence of 203 markers in common between the 26 chromosomes of the 2 maps. However, inconsistencies in marker order led to difficulties in integrating around one third of these QTLs. Apart from QTLs from Rong *et al. *[16] we also considered other literature sources of fiber QTL mapping [9,10,14,15,18], but they were not included in the meta-analysis.

The meta-analysis of QTLs from the RIL and BC data sets were run using the MetaQTL package [35]. The purpose of MetaQTL is to evaluate, for a given trait, the degree of congruence of the CI around LOD peaks detected in different mapping experiments. MetaQTL offers a statistical process to establish a consensus model for marker and QTL positions and to develop a clustering approach. The first 2 modules, *ConsMap *and *QTLProj*, of MetaQTL apply a weighted least square strategy for positioning markers on a single consensus map and for positioning QTLs on this map,, respectively. Then a clustering approach implemented in the *QTLClust *module and based on a Gaussian mixture model, determines the optimal number, K, of clusters by means of 4 different information-based criteria, including the Akaike Information Criterion (AIC) that is the only criteria presented here. Parameter estimates of MetaQTL include the most likely location on the chromosomes of the K clusters, their 95% CI, and the probability of individual QTLs belonging to a particular cluster.

Because clustering results are strongly influenced by the CI that frames the QTL, we ran the clustering with MetaQTL using 2 series of input data as CI of QTLs: - one-LOD drop off method as derived from composite interval mapping by WinQTL Cartographer, and - calculation-based method as described in Darvasi and Soller [40]. In this method, a weighted CI is calculated as the product of a constant value (function of the population type) divided by N*R² (N, population size and R², percentage of variance explained).

For a given chromosome, QTLs within the same broad trait category (thus possibly including different, but correlated traits, like ML, UHML and UQLw in case of fiber length category, or Rd and + b for fiber color category, etc..) and for different data sets were selected for clustering when QTLs with additivity of similar directionality were detected in at least 4 different populations/data sets (RIL or BC). The regions showing some degree of consistency across populations corresponded to "confirmed" QTLs, according to the terminology of Lander and Kruglyak [43]. The following nomenclature system was adopted to designate clusters: *QTLClust_Trait_Chr_Rank *(for example *QTLClust_FIN_3_2*, for the second cluster of fiber fineness QTLs along chromosome 3).

In several instances, it was proposed that clusters mapped at close proximity be coalesced into meta-clusters; in such cases the meta-cluster was referred-to by trait acronym followed by the chromosome number and by a letter suffix when necessary, such as *FIN_21B *for a second fineness meta-cluster on chromosome 21.

## Authors' contributions

JJe, CV, MC, JML participated in the RIL experiment conducted in Montpellier. PO and SG conducted the RIL experiment in Cameroon. MG, PAVB and HdA conducted RIL experiments in Brazil. GG and MV conducted fiber analyses for experiments conducted in France, Brazil and Cameroon, and provided global interpretation of fiber parameters. JJa co-ordinated all Bayer CS contributions and conducted the experiment in Belgium. DB, SC and TA conducted the RIL experiments in the USA. DL co-ordinated all CSIRO contributions and drafted the manuscript. SL and YAG conducted the RIL experiments in Australia. JML conceived and co-ordinated the project, conducted meta-analyses and drafted the manuscript. All authors contributed to the interpretation of the results, read and approved the final manuscript.

## Supplementary Material

Additional file 1 **Table S1: Details of significant QTLs in RIL and BC data sets**. Click here for file

Additional file 2 **Tables S2: Distribution of QTLs in BC experiments**. Distribution of the 67 significant QTLs and of the total 255 (including putative QTLs shown in parentheses) from the analysis of the 3 BC generations and shared among the 6 fiber trait categories, 3 generations and 26 chromosomes. Click here for file

Additional file 3 **Figure S1 (continuation of Figure **1**): Same legend as Figure **1. Click here for file

Additional file 4 **Table S3: Characteristics of the clusters detected by MetaQTL and list of meta-clusters**. Click here for file

Additional file 5 **Figure S2 (continuation of Figure **2**): Same legend as Figure **2. Click here for file

Additional file 6 **Table S4: Detailed chromosome by chromosome descriptions of the QTL clusters identified by MetaQTL**. Click here for file
