# Supplementary material for: Meta-analysis of cotton fiber quality QTLs across diverse environments in a Gossypium hirsutum x G. barbadense RIL population
Source: BMC Plant Biol. 2010 Jun 28;10:132. doi: 10.1186/1471-2229-10-132 (PMC3017793; doi:10.1186/1471-2229-10-132)
Supplement: Additional file 1 — Table S1: Details of significant QTLs in RIL and BC data sets. [file 1471-2229-10-132-S1.DOC]

**Additional file, Table S1:** Details of significant QTLs (LOD> permutation-based) detected in the 11 RIL experiments (167 QTLs) and 3 BC generations (67 QTLs). Support interval of QTLs are either based upon the one-LOD drop-off method (output information from Win QTLCartographer) or used the formula described in Darvasi and Soller [38], CI=163/(N*R²) for RILs and CI=530/(N*R²) for BC, with N population size and R² percentage of variance explained.

| **QTL name** | **Site,  generation** | **Fiber trait** | **Trait Category** | **Chrom.** | **cM** | **LOD** | **R²** | **Addi- vity** | **CI left one-LOD** | **CI right one-LOD** | **CI left formula** | **CI right formula** |
| --- | --- | --- | --- | --- | --- | --- | --- | --- | --- | --- | --- | --- |
| Lu7_Rd_1_1_4.24_[-] | Lu7 | Rd | color | **1** | 3.8 | 4.2 | 0.19 | -1.58 | 1.1 | 4.8 | 0.0 | 10.2 |
| Mp7_ML_1_1_3.44_[-] | Mp7 | ML | length | **1** | 30.8 | 3.4 | 0.14 | -0.97 | 30.8 | 38.5 | 24.6 | 37.0 |
| Mp8_b_1_1_3.55_[+] | Mp8 | b | color | **1** | 55.5 | 3.6 | 0.11 | 0.48 | 53.0 | 57.6 | 44.6 | 66.5 |
| Mp8_H_1_1_3.49_[-] | Mp8 | H | fineness | **1** | 94.1 | 3.5 | 0.16 | -11.31 | 88.7 | 96.1 | 86.7 | 97.9 |
| Ga7_H_2_4_5.64_[-] | Ga7 | H | fineness | **2** | 35.0 | 5.6 | 0.15 | -11.44 | 34.5 | 35.7 | 28.6 | 41.4 |
| Br8_Elo_2_1_3.91_[+] | Br8 | Elo | elongation | **2** | 39.1 | 3.9 | 0.10 | 0.14 | 38.4 | 44.3 | 32.6 | 45.6 |
| Mp8_Elo_2_2_3.98_[+] | Mp8 | Elo | elongation | **2** | 39.8 | 4.0 | 0.11 | 0.24 | 39.1 | 54.3 | 28.8 | 50.9 |
| Cs8_Elo_2_2_4.13_[+] | Cs8 | Elo | elongation | **2** | 39.8 | 4.1 | 0.14 | 0.17 | 39.5 | 46.2 | 30.9 | 48.7 |
| Cs7_Fin_3_1_4.58_[+] | Cs7 | Fin | fineness | **3** | 0.0 | 4.6 | 0.13 | 9.51 | 0.0 | 3.1 | 0.0 | 6.3 |
| Lu7_UQLw_3_1_7.62_[-] | Lu7 | UQLw | length | **3** | 8.0 | 7.6 | 0.33 | -2.02 | 5.9 | 11.0 | 4.4 | 11.6 |
| Ga7_UHML_3_1_5.16_[-] | Ga7 | UHML | length | **3** | 9.0 | 5.2 | 0.18 | -1.55 | 4.9 | 12.2 | 3.6 | 14.4 |
| Ga7_ML_3_1_5.47_[-] | Ga7 | ML | length | **3** | 9.0 | 5.5 | 0.19 | -1.50 | 5.5 | 11.9 | 3.8 | 14.2 |
| Lu7_UHML_3_1_4.37_[-] | Lu7 | UHML | length | **3** | 20.5 | 4.4 | 0.16 | -1.19 | 17.2 | 20.9 | 13.0 | 28.0 |
| Mp8_UHML_3_1_3.88_[+] | Mp8 | UHML | length | **3** | 54.3 | 3.9 | 0.14 | 1.14 | 52.6 | 56.9 | 45.3 | 63.3 |
| Lu8_MIC_4_1_4.08_[-] | Lu8 | MIC | fineness | **4** | 39.4 | 4.1 | 0.14 | -0.32 | 37.4 | 42.0 | 33.0 | 45.8 |
| Ga7_MR_4_1_4.51_[-] | Ga7 | MR | fineness | **4** | 42.7 | 4.5 | 0.15 | -0.07 | 41.1 | 45.3 | 36.2 | 49.2 |
| Lu7_UQLw_4_1_3.78_[+] | Lu7 | UQLw | length | **4** | 56.5 | 3.8 | 0.14 | 1.17 | 52.1 | 66.3 | 47.6 | 65.4 |
| Ga7_UHML_4_1_6.78_[+] | Ga7 | UHML | length | **4** | 56.5 | 6.8 | 0.23 | 1.47 | 54.2 | 60.1 | 52.3 | 60.7 |
| Ga7_ML_4_1_6.59_[+] | Ga7 | ML | length | **4** | 56.5 | 6.6 | 0.23 | 1.37 | 54.5 | 60.5 | 52.4 | 60.7 |
| Lu7_Hs_4_2_5.36_[-] | Lu7 | Hs | fineness | **4** | 60.6 | 5.4 | 0.20 | -8.03 | 57.5 | 66.3 | 54.5 | 66.7 |
| Mp8_ML_4_1_5.77_[+] | Mp8 | ML | length | **4** | 61.6 | 5.8 | 0.21 | 1.41 | 60.6 | 65.9 | 55.8 | 67.4 |
| Mp8_UHML_4_1_8.55_[+] | Mp8 | UHML | length | **4** | 61.6 | 8.5 | 0.35 | 1.81 | 60.6 | 66.6 | 58.2 | 65.0 |
| Mp8_STR_4_1_3.95_[+] | Mp8 | STR | strength | **4** | 63.6 | 3.9 | 0.16 | 1.51 | 59.7 | 67.6 | 56.0 | 69.7 |
| Mp8_UI_5_1_3.53_[+] | Mp8 | UI | uniformity | **5** | 12.6 | 3.5 | 0.14 | 1.09 | 12.5 | 18.5 | 4.2 | 21.1 |
| Mp8_Hs_5_1_6.96_[-] | Mp8 | Hs | fineness | **5** | 17.6 | 7.0 | 0.28 | -31.15 | 15.3 | 19.8 | 13.3 | 21.9 |
| Lu8_MIC_5_1_6.02_[+] | Lu8 | MIC | fineness | **5** | 21.7 | 6.0 | 0.20 | 0.40 | 20.6 | 22.0 | 17.2 | 26.2 |
| Br7_Hs_5_2_3.53_[+] | Br7 | Hs | fineness | **5** | 50.7 | 3.5 | 0.08 | 9.74 | 45.1 | 53.2 | 42.8 | 58.6 |
| Ge6_b_5_1_5.36_[+] | Ge6 | b | color | **5** | 92.0 | 5.4 | 0.13 | 0.78 | 90.9 | 92.5 | 75.3 | 108.7 |
| Mp7_UHML_6_1_4.25_[-] | Mp7 | UHML | length | **6** | 12.4 | 4.2 | 0.15 | -1.06 | 10.4 | 14.9 | 6.7 | 18.1 |
| Br8_b_6_1_4.45_[-] | Br8 | b | color | **6** | 14.2 | 4.5 | 0.12 | -0.54 | 12.4 | 15.3 | 8.9 | 19.5 |
| Ga7_b_6_1_5.00_[-] | Ga7 | b | color | **6** | 14.2 | 5.0 | 0.18 | -0.69 | 12.4 | 14.8 | 8.9 | 19.5 |
| Lu7_b_6_1_7.02_[-] | Lu7 | b | color | **6** | 14.2 | 7.0 | 0.29 | -0.80 | 12.4 | 14.8 | 10.1 | 18.4 |
| Lu7_Rd_6_1_7.32_[+] | Lu7 | Rd | color | **6** | 14.2 | 7.3 | 0.37 | 2.39 | 12.4 | 15.4 | 10.9 | 17.5 |
| Br7_Rd_6_1_5.71_[+] | Br7 | Rd | color | **6** | 14.2 | 5.7 | 0.14 | 1.83 | 13.9 | 17.4 | 9.6 | 18.8 |
| Lu8_Elo_6_1_3.44_[+] | Lu8 | Elo | elongation | **6** | 14.2 | 3.4 | 0.14 | 0.71 | 12.4 | 16.6 | 7.5 | 20.9 |
| Br7_H_6_1_6.19_[+] | Br7 | H | fineness | **6** | 32.3 | 6.2 | 0.16 | 12.77 | 28.2 | 34.0 | 28.1 | 36.5 |
| Br7_MICm_6_1_6.19_[+] | Br7 | MICm | fineness | **6** | 33.0 | 6.2 | 0.15 | 0.43 | 30.4 | 35.9 | 28.5 | 37.5 |
| Mp7_STR_7_4_5.22_[+] | Mp7 | STR | strength | **7** | 24.5 | 5.2 | 0.15 | 1.84 | 24.1 | 27.4 | 18.7 | 30.3 |
| Mp7_b_7_1_3.98_[+] | Mp7 | b | color | **7** | 54.4 | 4.0 | 0.11 | 0.49 | 49.9 | 61.5 | 47.0 | 61.9 |
| Ge6_Rd_7_1_6.05_[-] | Ge6 | Rd | color | **7** | 59.2 | 6.0 | 0.20 | -2.29 | 54.5 | 63.3 | 48.7 | 69.8 |
| Br7_b_8_2_3.95_[-] | Br7 | b | color | **8** | 13.3 | 3.9 | 0.09 | -0.46 | 12.0 | 17.1 | 5.8 | 20.8 |
| Ga7_Rd_8_1_3.72_[+] | Ga7 | Rd | color | **8** | 24.7 | 3.7 | 0.12 | 1.48 | 21.7 | 26.5 | 16.6 | 32.9 |
| Mp8_b_8_2_4.03_[-] | Mp8 | b | color | **8** | 33.1 | 4.0 | 0.13 | -0.54 | 30.7 | 35.2 | 23.8 | 42.4 |
| Mp8_Rd_8_3_4.69_[+] | Mp8 | Rd | color | **8** | 33.1 | 4.7 | 0.19 | 2.09 | 31.1 | 35.3 | 26.7 | 39.6 |
| Mp7_b_8_3_4.37_[-] | Mp7 | b | color | **8** | 33.1 | 4.4 | 0.11 | -0.53 | 31.6 | 37.0 | 25.6 | 40.7 |
| Ge6_Rd_8_3_10.60_[+] | Ge6 | Rd | color | **8** | 33.1 | 10.6 | 0.48 | 3.30 | 32.0 | 37.1 | 28.7 | 37.5 |
| Ge6_b_8_2_9.49_[-] | Ge6 | b | color | **8** | 33.1 | 9.5 | 0.30 | -0.74 | 32.2 | 37.2 | 25.9 | 40.3 |
| Br8_Rd_8_1_3.61_[+] | Br8 | Rd | color | **8** | 38.1 | 3.6 | 0.11 | 1.62 | 36.1 | 42.1 | 32.1 | 44.1 |
| Ga7_Rd_9_1_3.68_[+] | Ga7 | Rd | color | **9** | 3.3 | 3.7 | 0.12 | 1.37 | 0.2 | 8.2 | 0.0 | 11.5 |
| Cs8_SFI_9_2_6.22_[-] | Cs8 | SFI | uniformity | **9** | 7.3 | 6.2 | 0.32 | -1.84 | 5.3 | 11.3 | 3.4 | 11.2 |
| Ge6_ML_9_2_6.48_[+] | Ge6 | ML | length | **9** | 20.3 | 6.5 | 0.26 | 1.51 | 19.9 | 22.2 | 12.1 | 28.5 |
| Lu7_UQLw_9_2_9.46_[+] | Lu7 | UQLw | length | **9** | 20.3 | 9.5 | 0.37 | 1.88 | 19.9 | 21.8 | 17.1 | 23.6 |
| Ge6_STR_9_1_3.72_[+] | Ge6 | STR | strength | **9** | 20.3 | 3.7 | 0.12 | 1.60 | 19.6 | 23.3 | 2.4 | 38.2 |
| Mp8_Elo_9_2_4.00_[+] | Mp8 | Elo | elongation | **9** | 26.0 | 4.0 | 0.11 | 0.25 | 25.1 | 26.3 | 15.1 | 36.9 |
| Mp8_MICm_9_2_3.56_[+] | Mp8 | MICm | fineness | **9** | 26.0 | 3.6 | 0.14 | 0.33 | 25.1 | 26.4 | 17.3 | 34.8 |
| Mp8_b_9_3_3.64_[+] | Mp8 | b | color | **9** | 48.3 | 3.6 | 0.12 | 0.54 | 47.3 | 48.6 | 37.9 | 58.7 |
| Lu7_MIC_9_1_5.22_[+] | Lu7 | MIC | fineness | **9** | 57.3 | 5.2 | 0.16 | 0.46 | 56.6 | 59.3 | 49.7 | 64.9 |
| Lu7_MR_9_3_8.94_[+] | Lu7 | MR | fineness | **9** | 66.0 | 8.9 | 0.30 | 0.04 | 64.6 | 70.3 | 62.0 | 70.1 |
| Cs9_MIC_10_1_4.25_[+] | Cs9 | MIC | fineness | **10** | 15.5 | 4.2 | 0.18 | 0.31 | 12.5 | 18.5 | 8.5 | 22.5 |
| Ge6_b_11_1_6.70_[-] | Ge6 | b | color | **11** | 56.3 | 6.7 | 0.28 | -0.92 | 51.8 | 59.4 | 48.7 | 63.9 |
| Ge6_Rd_11_1_4.82_[+] | Ge6 | Rd | color | **11** | 56.3 | 4.8 | 0.26 | 2.86 | 51.9 | 59.4 | 48.2 | 64.4 |
| Br7_UI_12_1_4.39_[+] | Br7 | UI | uniformity | **12** | 0.0 | 4.4 | 0.11 | 0.89 | 0.0 | 0.9 | 0.0 | 6.2 |
| Cs9_MIC2_12_1_3.56_[+] | Cs9 | MIC2 | fineness | **12** | 1.7 | 3.6 | 0.13 | 0.26 | 0.0 | 6.6 | 0.0 | 11.3 |
| Br8_MICm_12_1_3.65_[+] | Br8 | MICm | fineness | **12** | 3.7 | 3.6 | 0.11 | 0.35 | 0.3 | 7.0 | 0.0 | 9.5 |
| Cs9_UI_12_1_5.08_[+] | Cs9 | UI | uniformity | **12** | 5.7 | 5.1 | 0.23 | 0.88 | 3.2 | 7.7 | 0.4 | 11.0 |
| Cs9_UI_12_2_5.94_[+] | Cs9 | UI | uniformity | **12** | 12.0 | 5.9 | 0.27 | 0.97 | 10.1 | 15.4 | 7.4 | 16.6 |
| Ge6_MIC_12_1_4.40_[+] | Ge6 | MIC | fineness | **12** | 19.8 | 4.4 | 0.12 | 0.37 | 16.5 | 22.6 | 1.5 | 38.1 |
| Cs9_MR_12_1_4.80_[+] | Cs9 | MR | fineness | **12** | 24.4 | 4.8 | 0.19 | 0.04 | 23.1 | 26.3 | 17.9 | 30.9 |
| Cs8_SFI_12_1_7.38_[-] | Cs8 | SFI | uniformity | **12** | 33.6 | 7.4 | 0.23 | -1.84 | 31.7 | 34.4 | 28.1 | 39.1 |
| Lu7_STR_12_1_4.41_[+] | Lu7 | STR | strength | **12** | 34.2 | 4.4 | 0.13 | 2.28 | 32.2 | 37.1 | 24.9 | 43.6 |
| Cs9_UI_12_3_7.82_[+] | Cs9 | UI | uniformity | **12** | 35.6 | 7.8 | 0.31 | 1.26 | 35.4 | 38.4 | 31.6 | 39.6 |
| Mp8_Elo_12_1_3.88_[+] | Mp8 | Elo | elongation | **12** | 35.6 | 3.9 | 0.11 | 0.32 | 34.2 | 36.3 | 24.1 | 47.1 |
| Ga7_H_12_1_4.15_[+] | Ga7 | H | fineness | **12** | 35.6 | 4.1 | 0.11 | 11.05 | 34.2 | 36.9 | 26.4 | 44.9 |
| Br7_MICm_12_1_4.85_[+] | Br7 | MICm | fineness | **12** | 35.6 | 4.9 | 0.12 | 0.40 | 34.2 | 37.0 | 29.9 | 41.3 |
| Ga7_UI_12_1_4.43_[+] | Ga7 | UI | uniformity | **12** | 35.6 | 4.4 | 0.10 | 1.09 | 34.2 | 36.8 | 26.2 | 45.0 |
| Lu8_Elo_12_2_3.35_[+] | Lu8 | Elo | elongation | **12** | 38.5 | 3.4 | 0.13 | 0.96 | 38.3 | 40.0 | 31.5 | 45.5 |
| Ge6_STR_12_1_7.17_[+] | Ge6 | STR | strength | **12** | 38.5 | 7.2 | 0.31 | 3.48 | 36.7 | 39.6 | 31.7 | 45.4 |
| Lu8_UI_12_3_6.32_[+] | Lu8 | UI | uniformity | **12** | 53.6 | 6.3 | 0.25 | 0.96 | 52.1 | 55.7 | 50.0 | 57.2 |
| Lu8_UQLw_13_1_5.55_[+] | Lu8 | UQLw | length | **13** | 9.9 | 5.5 | 0.22 | 0.05 | 6.7 | 17.8 | 5.8 | 14.0 |
| Mp8_Elo_13_1_5.29_[+] | Mp8 | Elo | elongation | **13** | 26.1 | 5.3 | 0.15 | 0.27 | 21.0 | 27.4 | 18.2 | 34.0 |
| Lu7_b_14_1_3.81_[+] | Lu7 | b | color | **14** | 21.8 | 3.8 | 0.13 | 0.51 | 20.1 | 30.0 | 12.6 | 31.0 |
| Mp8_STR_14_1_3.84_[-] | Mp8 | STR | strength | **14** | 56.5 | 3.8 | 0.15 | -1.48 | 55.8 | 57.3 | 48.2 | 64.8 |
| Mp8_ML_14_2_3.84_[-] | Mp8 | ML | length | **14** | 68.2 | 3.8 | 0.13 | -1.07 | 66.7 | 71.6 | 58.7 | 77.7 |
| Lu7_MIC_15_1_5.95_[+] | Lu7 | MIC | fineness | **15** | 32.0 | 6.0 | 0.20 | 0.37 | 28.3 | 34.7 | 26.0 | 38.0 |
| Lu7_Fin_15_1_4.17_[+] | Lu7 | Fin | fineness | **15** | 32.0 | 4.2 | 0.13 | 7.02 | 29.8 | 36.7 | 23.0 | 41.1 |
| Br7_Rd_15_1_4.64_[-] | Br7 | Rd | color | **15** | 34.9 | 4.6 | 0.12 | -1.53 | 33.9 | 35.3 | 29.4 | 40.4 |
| Br7_PM_15_3_3.59_[+] | Br7 | PM | fineness | **15** | 38.7 | 3.6 | 0.11 | 6.14 | 36.7 | 39.4 | 32.8 | 44.6 |
| Br8_Elo_15_2_4.84_[+] | Br8 | Elo | elongation | **15** | 39.4 | 4.8 | 0.12 | 0.21 | 37.9 | 42.0 | 34.2 | 44.6 |
| Br8_UI_15_2_6.22_[+] | Br8 | UI | uniformity | **15** | 41.4 | 6.2 | 0.16 | 1.11 | 38.9 | 43.0 | 37.5 | 45.3 |
| Br8_MR_15_2_3.44_[+] | Br8 | MR | fineness | **15** | 41.7 | 3.4 | 0.08 | 0.06 | 39.4 | 46.8 | 33.6 | 49.8 |
| Cs8_Elo_15_3_3.93_[+] | Cs8 | Elo | elongation | **15** | 41.7 | 3.9 | 0.14 | 0.16 | 40.6 | 47.4 | 32.5 | 51.0 |
| Br7_PM_15_4_4.00_[+] | Br7 | PM | fineness | **15** | 41.7 | 4.0 | 0.10 | 5.68 | 39.4 | 42.8 | 35.1 | 48.3 |
| Ga7_Elo_15_1_4.88_[+] | Ga7 | Elo | elongation | **15** | 46.8 | 4.9 | 0.16 | 0.24 | 40.8 | 50.2 | 40.7 | 52.9 |
| Cs9_MIC2_15_1_3.55_[+] | Cs9 | MIC2 | fineness | **15** | 47.8 | 3.5 | 0.13 | 0.23 | 44.2 | 51.4 | 38.0 | 57.6 |
| Ga7_H_15_1_7.54_[+] | Ga7 | H | fineness | **15** | 47.8 | 7.5 | 0.22 | 13.76 | 44.2 | 51.9 | 43.3 | 52.3 |
| Cs9_Fin_15_1_3.57_[+] | Cs9 | Fin | fineness | **15** | 49.8 | 3.6 | 0.17 | 10.82 | 44.4 | 52.9 | 42.5 | 57.1 |
| Ga7_MR_15_1_6.41_[+] | Ga7 | MR | fineness | **15** | 49.8 | 6.4 | 0.24 | 0.09 | 46.0 | 52.9 | 45.8 | 53.8 |
| Ge6_b_15_1_4.64_[+] | Ge6 | b | color | **15** | 53.9 | 4.6 | 0.11 | 0.50 | 50.7 | 58.9 | 34.2 | 71.7 |
| Ge6_STR_15_1_4.85_[-] | Ge6 | STR | strength | **15** | 68.2 | 4.9 | 0.17 | -1.87 | 67.1 | 69.8 | 55.7 | 71.7 |
| Cs8_MIC_16_1_3.69_[+] | Cs8 | MIC | fineness | **16** | 5.5 | 3.7 | 0.15 | 0.33 | 3.9 | 8.6 | 0.0 | 13.9 |
| Br7_MICm_16_1_3.74_[+] | Br7 | MICm | fineness | **16** | 11.5 | 3.7 | 0.11 | 0.36 | 10.8 | 12.7 | 5.3 | 17.8 |
| Mp8_H_16_1_5.51_[+] | Mp8 | H | fineness | **16** | 12.7 | 5.5 | 0.22 | 15.01 | 9.2 | 15.3 | 7.2 | 18.2 |
| Mp8_UI_16_2_4.11_[+] | Mp8 | UI | uniformity | **16** | 15.5 | 4.1 | 0.13 | 1.04 | 15.5 | 17.9 | 6.2 | 24.9 |
| Ga7_Hs_16_1_4.82_[+] | Ga7 | Hs | fineness | **16** | 41.9 | 4.8 | 0.17 | 21.16 | 40.0 | 44.8 | 36.3 | 47.5 |
| Br8_MICm_17_1_4.03_[+] | Br8 | MICm | fineness | **17** | 20.7 | 4.0 | 0.10 | 0.29 | 17.8 | 23.4 | 14.1 | 27.3 |
| Br8_MR_17_1_4.10_[+] | Br8 | MR | fineness | **17** | 20.7 | 4.1 | 0.09 | 0.06 | 18.8 | 24.6 | 14.0 | 27.4 |
| Lu8_MR_17_1_4.04_[+] | Lu8 | MR | fineness | **17** | 20.7 | 4.0 | 0.13 | 0.02 | 18.4 | 24.1 | 13.8 | 27.6 |
| Cs7_MR_17_1_3.52_[+] | Cs7 | MR | fineness | **17** | 22.7 | 3.5 | 0.09 | 0.02 | 16.8 | 28.9 | 13.4 | 32.0 |
| Cs7_Fin_17_1_3.74_[+] | Cs7 | Fin | fineness | **17** | 32.1 | 3.7 | 0.10 | 7.97 | 30.3 | 33.2 | 23.5 | 40.7 |
| Cs9_MIC_18_1_4.96_[+] | Cs9 | MIC | fineness | **18** | 37.4 | 5.0 | 0.19 | 0.36 | 37.0 | 39.9 | 31.0 | 43.8 |
| Mp7_MIC_18_3_4.69_[+] | Mp7 | MIC | fineness | **18** | 39.4 | 4.7 | 0.14 | 0.34 | 37.4 | 42.6 | 33.5 | 45.3 |
| Lu8_MIC_18_1_6.52_[+] | Lu8 | MIC | fineness | **18** | 60.4 | 6.5 | 0.22 | 0.45 | 59.6 | 60.7 | 56.3 | 64.5 |
| Ge6_Rd_18_1_5.50_[+] | Ge6 | Rd | color | **18** | 96.7 | 5.5 | 0.18 | 1.99 | 93.5 | 98.7 | 84.8 | 99.7 |
| Lu7_STR_19_1_7.36_[+] | Lu7 | STR | strength | **19** | 6.0 | 7.4 | 0.27 | 3.15 | 3.3 | 10.2 | 1.6 | 10.5 |
| Cs8_PM_19_1_4.18_[+] | Cs8 | PM | fineness | **19** | 15.7 | 4.2 | 0.15 | 5.67 | 10.3 | 22.7 | 7.6 | 23.9 |
| Mp7_MR_19_1_4.05_[+] | Mp7 | MR | fineness | **19** | 35.8 | 4.1 | 0.11 | 0.02 | 35.3 | 38.2 | 28.3 | 43.3 |
| Lu7_MR_19_1_3.76_[+] | Lu7 | MR | fineness | **19** | 37.8 | 3.8 | 0.10 | 0.03 | 34.5 | 38.5 | 25.9 | 49.7 |
| Ga7_UI_19_2_4.31_[+] | Ga7 | UI | uniformity | **19** | 37.8 | 4.3 | 0.11 | 1.35 | 37.2 | 39.7 | 28.7 | 46.9 |
| Ge6_ML_19_2_8.61_[+] | Ge6 | ML | length | **19** | 37.9 | 8.6 | 0.40 | 2.70 | 37.7 | 39.6 | 32.6 | 43.3 |
| Lu7_STR_19_4_5.93_[+] | Lu7 | STR | strength | **19** | 37.9 | 5.9 | 0.19 | 3.42 | 37.5 | 39.4 | 31.5 | 44.3 |
| Br7_Elo_19_1_4.72_[+] | Br7 | Elo | elongation | **19** | 39.9 | 4.7 | 0.15 | 0.25 | 37.9 | 40.8 | 35.6 | 44.2 |
| Br8_Elo_19_2_3.91_[+] | Br8 | Elo | elongation | **19** | 43.0 | 3.9 | 0.10 | 0.27 | 42.8 | 43.4 | 36.5 | 49.5 |
| Mp8_ML_19_2_3.79_[+] | Mp8 | ML | length | **19** | 44.3 | 3.8 | 0.12 | 0.97 | 44.0 | 46.1 | 34.3 | 54.3 |
| Ge6_MIC_20_1_6.87_[-] | Ge6 | MIC | fineness | **20** | 49.8 | 6.9 | 0.25 | -0.56 | 48.8 | 54.0 | 41.1 | 58.5 |
| Mp7_MIC_20_2_4.62_[+] | Mp7 | MIC | fineness | **20** | 77.6 | 4.6 | 0.15 | 0.32 | 72.9 | 83.3 | 71.8 | 83.4 |
| Br7_Rd_21_1_3.77_[+] | Br7 | Rd | color | **21** | 6.6 | 3.8 | 0.08 | 1.32 | 2.8 | 10.6 | 0.0 | 14.5 |
| Br8_Rd_21_1_4.26_[+] | Br8 | Rd | color | **21** | 8.6 | 4.3 | 0.12 | 1.59 | 3.4 | 11.4 | 3.2 | 14.0 |
| Mp7_STR_21_1_3.34_[-] | Mp7 | STR | strength | **21** | 32.9 | 3.3 | 0.09 | -1.36 | 28.8 | 39.9 | 23.3 | 42.6 |
| Br7_UHML_21_1_4.66_[+] | Br7 | UHML | length | **21** | 60.3 | 4.7 | 0.13 | 1.09 | 57.5 | 61.3 | 55.4 | 65.2 |
| Br7_ML_21_2_5.01_[+] | Br7 | ML | length | **21** | 60.3 | 5.0 | 0.15 | 1.09 | 57.7 | 61.1 | 55.9 | 64.7 |
| Lu8_Elo_21_1_3.52_[+] | Lu8 | Elo | elongation | **21** | 60.6 | 3.5 | 0.13 | 0.59 | 58.1 | 77.1 | 53.8 | 67.4 |
| Lu7_Hs_21_2_3.59_[+] | Lu7 | Hs | fineness | **21** | 62.3 | 3.6 | 0.12 | 5.27 | 61.4 | 63.5 | 52.3 | 72.3 |
| Lu8_STR_21_3_7.47_[-] | Lu8 | STR | strength | **21** | 62.3 | 7.5 | 0.28 | -1.64 | 61.5 | 65.8 | 59.1 | 65.5 |
| Br7_H_21_1_5.24_[+] | Br7 | H | fineness | **21** | 75.1 | 5.2 | 0.15 | 10.57 | 71.4 | 78.7 | 70.7 | 79.6 |
| Cs9_Hs_21_1_3.48_[+] | Cs9 | Hs | fineness | **21** | 77.1 | 3.5 | 0.16 | 7.60 | 67.4 | 79.8 | 69.4 | 84.8 |
| Lu7_Fin_21_3_3.99_[+] | Lu7 | Fin | fineness | **21** | 134.2 | 4.0 | 0.14 | 8.37 | 132.0 | 137.1 | 125.4 | 143.0 |
| Lu8_Fin_21_1_4.17_[+] | Lu8 | Fin | fineness | **21** | 148.4 | 4.2 | 0.13 | 8.70 | 145.7 | 151.1 | 141.6 | 155.3 |
| Br7_Elo_21_1_4.92_[+] | Br7 | Elo | elongation | **21** | 159.8 | 4.9 | 0.11 | 0.21 | 155.4 | 162.5 | 153.8 | 165.8 |
| Br8_Rd_22_1_3.66_[+] | Br8 | Rd | color | **22** | 0.0 | 3.7 | 0.09 | 1.37 | 0.0 | 11.8 | 0.0 | 7.5 |
| Lu7_MIC_22_1_4.26_[+] | Lu7 | MIC | fineness | **22** | 16.2 | 4.3 | 0.13 | 0.31 | 9.1 | 17.3 | 7.1 | 25.3 |
| Cs8_MR_23_1_4.26_[+] | Cs8 | MR | fineness | **23** | 0.0 | 4.3 | 0.16 | 0.06 | 0.0 | 7.7 | 0.0 | 8.1 |
| Ga7_UI_23_1_4.15_[+] | Ga7 | UI | uniformity | **23** | 28.4 | 4.1 | 0.10 | 0.86 | 27.0 | 30.9 | 18.6 | 38.3 |
| Ge6_MIC_24_2_7.14_[+] | Ge6 | MIC | fineness | **24** | 7.4 | 7.1 | 0.41 | 0.60 | 2.1 | 9.7 | 2.2 | 12.6 |
| Ge6_MIC_24_3_4.85_[-] | Ge6 | MIC | fineness | **24** | 29.8 | 4.8 | 0.14 | -0.32 | 26.0 | 33.8 | 14.2 | 45.5 |
| Cs8_UHML_24_1_3.79_[-] | Cs8 | UHML | length | **24** | 53.2 | 3.8 | 0.14 | -1.01 | 52.9 | 57.3 | 44.5 | 61.9 |
| Cs9_UHML_24_1_4.45_[-] | Cs9 | UHML | length | **24** | 63.6 | 4.5 | 0.18 | -1.06 | 62.5 | 69.1 | 56.9 | 70.3 |
| Mp8_H_25_1_5.74_[+] | Mp8 | H | fineness | **25** | 10.3 | 5.7 | 0.26 | 15.10 | 4.7 | 15.8 | 5.5 | 15.1 |
| Mp8_MICf_25_1_3.74_[+] | Mp8 | MICf | fineness | **25** | 11.6 | 3.7 | 0.16 | 0.32 | 3.5 | 16.3 | 4.1 | 19.1 |
| Br8_MICm_25_1_3.43_[+] | Br8 | MICm | fineness | **25** | 18.2 | 3.4 | 0.08 | 0.31 | 15.0 | 19.2 | 10.5 | 25.9 |
| Cs7_Fin_25_1_4.88_[+] | Cs7 | Fin | fineness | **25** | 18.2 | 4.9 | 0.13 | 9.51 | 16.1 | 18.7 | 12.0 | 24.4 |
| Cs9_MIC2_25_1_4.69_[+] | Cs9 | MIC2 | fineness | **25** | 19.1 | 4.7 | 0.17 | 0.32 | 18.4 | 21.6 | 11.9 | 26.3 |
| Cs9_Fin_25_1_4.72_[+] | Cs9 | Fin | fineness | **25** | 21.1 | 4.7 | 0.22 | 14.13 | 19.5 | 23.5 | 15.5 | 26.7 |
| Cs7_Hs_25_2_3.77_[+] | Cs7 | Hs | fineness | **25** | 24.9 | 3.8 | 0.10 | 6.24 | 21.8 | 30.4 | 17.0 | 32.8 |
| Ga7_b_25_1_4.17_[-] | Ga7 | b | color | **25** | 28.9 | 4.2 | 0.14 | -0.60 | 26.0 | 33.9 | 22.0 | 35.8 |
| Br7_b_25_1_4.69_[-] | Br7 | b | color | **25** | 28.9 | 4.7 | 0.12 | -0.58 | 26.6 | 33.1 | 23.2 | 34.6 |
| Mp7_b_25_2_6.13_[-] | Mp7 | b | color | **25** | 28.9 | 6.1 | 0.18 | -0.63 | 27.4 | 32.7 | 24.1 | 33.8 |
| Ge6_UHML_26_2_3.68_[+] | Ge6 | UHML | length | **26** | 22.7 | 3.7 | 0.15 | 0.98 | 22.1 | 28.2 | 8.4 | 37.0 |
| Br7_Hs_26_1_4.31_[-] | Br7 | Hs | fineness | **26** | 28.7 | 4.3 | 0.12 | -13.19 | 28.0 | 32.2 | 23.4 | 34.0 |
| Ga7_Hs_26_1_6.20_[-] | Ga7 | Hs | fineness | **26** | 37.8 | 6.2 | 0.23 | -25.98 | 36.3 | 39.1 | 33.6 | 42.0 |
| Br8_Hs_26_2_5.62_[-] | Br8 | Hs | fineness | **26** | 41.6 | 5.6 | 0.22 | -18.04 | 39.6 | 44.3 | 38.7 | 44.5 |
| Br7_Elo_26_1_3.72_[+] | Br7 | Elo | elongation | **26** | 48.3 | 3.7 | 0.10 | 0.22 | 45.5 | 50.3 | 41.7 | 54.9 |
| Mp7_STR_26_2_8.47_[+] | Mp7 | STR | strength | **26** | 50.3 | 8.5 | 0.28 | 2.52 | 49.1 | 52.0 | 47.2 | 53.4 |
| Lu7_UHML_26_1_5.80_[+] | Lu7 | UHML | length | **26** | 54.0 | 5.8 | 0.22 | 1.48 | 51.4 | 54.6 | 48.6 | 59.4 |
| Cs8_SFI_26_2_6.87_[-] | Cs8 | SFI | uniformity | **26** | 54.0 | 6.9 | 0.21 | -1.49 | 53.4 | 66.8 | 48.1 | 60.0 |
| Lu7_ML_26_1_3.80_[+] | Lu7 | ML | length | **26** | 56.6 | 3.8 | 0.14 | 1.28 | 53.6 | 58.3 | 48.0 | 65.2 |
| Cs8_ML_26_2_6.53_[+] | Cs8 | ML | length | **26** | 56.6 | 6.5 | 0.27 | 1.52 | 55.5 | 58.0 | 52.0 | 61.3 |
| Cs8_UHML_26_2_4.21_[+] | Cs8 | UHML | length | **26** | 58.7 | 4.2 | 0.22 | 1.31 | 56.9 | 60.6 | 53.0 | 64.4 |
| **QTL name** | **Site,  generation** | **Fiber trait** | **Trait Category** | **Chrom.** | **cM** | **LOD** | **R²** | **addi- vity** | **CI left one-LOD** | **CI right one-LOD** | **CI left formula** | **CI right formula** |
| BC2S1_MIC_2_1_3.33_[-] | BC2S1 | MIC | fineness | **2** | 19.0 | 3.3 | 0.06 | -0.19 | 14.3 | 22.6 | 0.0 | 41.5 |
| BC1_UHML_3_2_4.14_[-] | BC1 | UHML | length | **3** | 35.9 | 4.1 | 0.13 | -1.39 | 35.2 | 36.4 | 8.3 | 63.5 |
| BC2_MR_3_2_4.67_[+] | BC2 | MR | fineness | **3** | 49.5 | 4.7 | 0.09 | 0.07 | 48.2 | 51.8 | 35.4 | 63.6 |
| BC2_MIC_3_2_4.25_[+] | BC2 | MIC | fineness | **3** | 49.5 | 4.2 | 0.08 | 0.39 | 48.2 | 57.0 | 32.8 | 66.2 |
| BC2S1_MR_3_2_4.05_[+] | BC2S1 | MR | fineness | **3** | 49.5 | 4.0 | 0.07 | 0.03 | 49.0 | 56.1 | 31.2 | 67.9 |
| BC2S1_UHML_3_2_6.92_[-] | BC2S1 | UHML | length | **3** | 49.5 | 6.9 | 0.12 | -0.98 | 49.2 | 51.8 | 38.6 | 60.4 |
| BC2S1_ML_3_2_4.48_[-] | BC2S1 | ML | length | **3** | 49.5 | 4.5 | 0.08 | -0.77 | 48.2 | 55.1 | 33.1 | 65.9 |
| BC1_STR_3_1_4.94_[-] | BC1 | STR | strength | **3** | 62.6 | 4.9 | 0.18 | -2.30 | 61.0 | 68.4 | 42.6 | 82.6 |
| BC1_STR_3_2_5.70_[-] | BC1 | STR | strength | **3** | 73.0 | 5.7 | 0.20 | -2.48 | 69.2 | 77.9 | 55.3 | 90.8 |
| BC2_Hs_4_2_3.44_[+] | BC2 | Hs | fineness | **4** | 45.3 | 3.4 | 0.07 | 18.37 | 43.7 | 54.4 | 26.1 | 64.5 |
| BC1_Hs_5_1_3.82_[-] | BC1 | Hs | fineness | **5** | 27.7 | 3.8 | 0.12 | -21.40 | 21.5 | 30.5 | 0.0 | 56.0 |
| BC1_STR_5_3_3.98_[-] | BC1 | STR | strength | **5** | 60.7 | 4.0 | 0.13 | -1.96 | 59.3 | 64.1 | 34.3 | 87.1 |
| BC1_MIC_6_1_5.43_[+] | BC1 | MIC | fineness | **6** | 0.0 | 5.4 | 0.19 | 0.47 | 0.0 | 2.8 | 0.0 | 18.3 |
| BC1_H_6_1_3.72_[+] | BC1 | H | fineness | **6** | 0.0 | 3.7 | 0.13 | 11.54 | 0.0 | 5.1 | 0.0 | 27.4 |
| BC1_MR_6_1_3.73_[+] | BC1 | MR | fineness | **6** | 0.0 | 3.7 | 0.13 | 0.08 | 0.0 | 3.9 | 0.0 | 27.8 |
| BC2_UHML_6_4_3.28_[-] | BC2 | UHML | length | **6** | 56.7 | 3.3 | 0.06 | -1.19 | 56.5 | 59.7 | 33.0 | 80.4 |
| BC2S1_Rd_8_3_5.18_[+] | BC2S1 | Rd | color | **8** | 88.0 | 5.2 | 0.08 | 1.03 | 87.4 | 90.3 | 71.9 | 104.2 |
| BC1_MIC_8_2_4.12_[-] | BC1 | MIC | fineness | **8** | 121.5 | 4.1 | 0.15 | -0.41 | 119.8 | 124.0 | 98.7 | 144.3 |
| BC1_MR_8_2_3.84_[-] | BC1 | MR | fineness | **8** | 121.5 | 3.8 | 0.14 | -0.08 | 116.8 | 124.1 | 96.3 | 146.7 |
| BC1_H_8_2_3.83_[-] | BC1 | H | fineness | **8** | 137.3 | 3.8 | 0.13 | -11.39 | 133.9 | 139.8 | 110.9 | 163.7 |
| BC1_Elo_9_1_5.39_[+] | BC1 | Elo | elongation | **9** | 64.5 | 5.4 | 0.18 | 0.33 | 61.1 | 67.0 | 44.8 | 84.2 |
| BC1_MIC_10_2_5.62_[+] | BC1 | MIC | fineness | **10** | 16.6 | 5.6 | 0.21 | 0.57 | 14.8 | 19.1 | 0.0 | 33.7 |
| BC2_Elo_10_3_4.72_[-] | BC2 | Elo | elongation | **10** | 98.6 | 4.7 | 0.08 | -0.48 | 93.7 | 104.1 | 83.0 | 114.3 |
| BC2_ML_13_1_4.05_[-] | BC2 | ML | length | **13** | 0.0 | 4.1 | 0.07 | -1.23 | 0.0 | 41.1 | 0.0 | 18.9 |
| BC1_UI_16_1_3.97_[-] | BC1 | UI | uniformity | **16** | 0.0 | 4.0 | 0.14 | -0.98 | 0.0 | 10.0 | 0.0 | 25.8 |
| BC2S1_STR_16_1_3.38_[-] | BC2S1 | STR | strength | **16** | 44.8 | 3.4 | 0.08 | -1.31 | 44.0 | 60.0 | 27.6 | 62.0 |
| BC2_Rd_17_2_5.27_[+] | BC2 | Rd | color | **17** | 29.4 | 5.3 | 0.12 | 2.94 | 28.9 | 32.3 | 18.8 | 40.1 |
| BC2S1_UI_18_1_6.11_[+] | BC2S1 | UI | uniformity | **18** | 23.8 | 6.1 | 0.11 | 0.84 | 20.5 | 26.7 | 11.5 | 36.1 |
| BC1_MR_18_1_4.91_[-] | BC1 | MR | fineness | **18** | 43.2 | 4.9 | 0.17 | -0.09 | 35.4 | 43.8 | 23.0 | 63.5 |
| BC1_Hs_18_1_3.61_[+] | BC1 | Hs | fineness | **18** | 44.1 | 3.6 | 0.12 | 19.56 | 37.0 | 52.6 | 13.9 | 74.4 |
| BC2_UI_18_1_6.49_[+] | BC2 | UI | uniformity | **18** | 66.6 | 6.5 | 0.12 | 2.17 | 61.1 | 71.3 | 55.9 | 77.3 |
| BC2_STR_18_1_4.03_[+] | BC2 | STR | strength | **18** | 90.7 | 4.0 | 0.09 | 3.03 | 80.6 | 98.4 | 76.5 | 105.0 |
| BC2_ML_18_1_6.40_[+] | BC2 | ML | length | **18** | 94.7 | 6.4 | 0.15 | 2.26 | 87.8 | 104.1 | 85.8 | 103.6 |
| BC2_Rd_18_1_4.57_[+] | BC2 | Rd | color | **18** | 102.4 | 4.6 | 0.09 | 3.09 | 91.2 | 109.2 | 87.5 | 117.3 |
| BC2_H_19_1_4.00_[-] | BC2 | H | fineness | **19** | 8.0 | 4.0 | 0.09 | -15.35 | 0.0 | 29.2 | 0.0 | 22.9 |
| BC1_ML_19_1_7.09_[+] | BC1 | ML | length | **19** | 61.4 | 7.1 | 0.25 | 1.97 | 57.1 | 65.0 | 47.5 | 75.3 |
| BC1_UHML_19_1_6.99_[+] | BC1 | UHML | length | **19** | 61.4 | 7.0 | 0.24 | 1.99 | 58.3 | 63.4 | 46.7 | 76.1 |
| BC2_Rd_19_1_4.67_[+] | BC2 | Rd | color | **19** | 62.5 | 4.7 | 0.08 | 2.86 | 60.9 | 67.1 | 46.2 | 78.9 |
| BC1_MIC_19_1_4.74_[-] | BC1 | MIC | fineness | **19** | 97.0 | 4.7 | 0.16 | -0.42 | 91.5 | 99.3 | 74.9 | 119.1 |
| BC1_H_19_1_4.01_[-] | BC1 | H | fineness | **19** | 97.7 | 4.0 | 0.14 | -11.54 | 88.8 | 101.5 | 72.6 | 122.8 |
| BC2S1_Elo_19_2_6.69_[+] | BC2S1 | Elo | elongation | **19** | 134.7 | 6.7 | 0.12 | 0.37 | 134.0 | 137.4 | 123.8 | 145.6 |
| BC2S1_Rd_19_1_3.77_[-] | BC2S1 | Rd | color | **19** | 135.3 | 3.8 | 0.06 | -0.98 | 134.6 | 155.5 | 112.9 | 157.8 |
| BC2_Elo_19_2_3.56_[+] | BC2 | Elo | elongation | **19** | 135.3 | 3.6 | 0.06 | 0.41 | 134.6 | 141.0 | 112.3 | 158.3 |
| BC2S1_UHML_19_1_3.39_[-] | BC2S1 | UHML | length | **19** | 135.3 | 3.4 | 0.06 | -0.80 | 133.5 | 157.6 | 112.3 | 158.3 |
| BC1_Elo_19_2_5.74_[+] | BC1 | Elo | elongation | **19** | 144.0 | 5.7 | 0.20 | 0.34 | 140.8 | 147.1 | 126.4 | 161.6 |
| BC1_Rd_19_4_3.92_[-] | BC1 | Rd | color | **19** | 153.4 | 3.9 | 0.12 | -2.39 | 150.3 | 156.0 | 124.1 | 182.7 |
| BC2_UI_20_2_3.97_[-] | BC2 | UI | uniformity | **20** | 29.8 | 4.0 | 0.09 | -1.47 | 29.0 | 35.9 | 14.8 | 44.9 |
| BC2_Elo_20_1_6.05_[-] | BC2 | Elo | elongation | **20** | 33.8 | 6.0 | 0.12 | -0.50 | 29.1 | 41.1 | 23.2 | 44.5 |
| BC2_UHML_20_2_3.29_[-] | BC2 | UHML | length | **20** | 112.3 | 3.3 | 0.06 | -1.19 | 103.8 | 114.3 | 88.6 | 136.0 |
| BC2S1_MR_21_2_6.12_[+] | BC2S1 | MR | fineness | **21** | 65.1 | 6.1 | 0.11 | 0.06 | 64.4 | 67.6 | 53.5 | 76.7 |
| BC1_Elo_21_2_5.97_[-] | BC1 | Elo | elongation | **21** | 89.1 | 6.0 | 0.21 | -0.36 | 86.3 | 96.0 | 72.5 | 105.7 |
| BC2_Elo_23_1_4.73_[-] | BC2 | Elo | elongation | **23** | 21.3 | 4.7 | 0.08 | -0.54 | 16.4 | 24.6 | 4.1 | 38.5 |
| BC1_STR_23_2_4.34_[-] | BC1 | STR | strength | **23** | 98.4 | 4.3 | 0.15 | -2.06 | 93.8 | 101.5 | 74.5 | 122.3 |
| BC2_STR_23_4_4.05_[-] | BC2 | STR | strength | **23** | 100.3 | 4.1 | 0.07 | -2.32 | 95.3 | 103.6 | 81.1 | 119.5 |
| BC1_UHML_23_1_3.60_[-] | BC1 | UHML | length | **23** | 116.3 | 3.6 | 0.13 | -1.45 | 109.8 | 118.3 | 89.9 | 128.5 |
| BC2S1_MIC_24_1_4.71_[-] | BC2S1 | MIC | fineness | **24** | 8.0 | 4.7 | 0.12 | -0.25 | 0.0 | 17.6 | 0.0 | 19.5 |
| BC2S1_H_24_1_3.68_[-] | BC2S1 | H | fineness | **24** | 16.0 | 3.7 | 0.08 | -9.39 | 3.5 | 20.2 | 0.0 | 32.6 |
| BC2_Hs_25_1_3.62_[+] | BC2 | Hs | fineness | **25** | 14.2 | 3.6 | 0.08 | 20.93 | 0.3 | 24.5 | 0.0 | 31.2 |
| BC1_Rd_25_2_5.52_[+] | BC1 | Rd | color | **25** | 49.5 | 5.5 | 0.16 | 2.88 | 49.1 | 54.2 | 27.8 | 71.2 |
| BC1_b_25_1_5.09_[-] | BC1 | b | color | **25** | 57.7 | 5.1 | 0.24 | -1.07 | 55.7 | 58.4 | 43.0 | 72.4 |
| BC2S1_Rd_25_1_4.12_[+] | BC2S1 | Rd | color | **25** | 57.7 | 4.1 | 0.07 | 0.99 | 50.2 | 64.4 | 39.3 | 76.1 |
| BC2S1_b_25_2_6.35_[-] | BC2S1 | b | color | **25** | 69.2 | 6.4 | 0.12 | -0.49 | 65.6 | 69.3 | 58.1 | 80.3 |
| BC2_b_25_2_5.09_[-] | BC2 | b | color | **25** | 79.8 | 5.1 | 0.10 | -0.92 | 79.4 | 81.1 | 66.2 | 93.4 |
| BC2_b_25_4_3.32_[-] | BC2 | b | color | **25** | 88.9 | 3.3 | 0.07 | -0.73 | 87.2 | 90.2 | 68.6 | 109.2 |
| BC2_UHML_26_1_4.22_[-] | BC2 | UHML | length | **26** | 10.0 | 4.2 | 0.09 | -1.59 | 3.0 | 18.7 | 0.0 | 24.4 |
| BC2S1_UHML_26_3_3.87_[-] | BC2S1 | UHML | length | **26** | 53.1 | 3.9 | 0.07 | -0.78 | 50.6 | 58.8 | 34.4 | 71.8 |
