# Supplementary material for: Meta-analysis of cotton fiber quality QTLs across diverse environments in a Gossypium hirsutum x G. barbadense RIL population
Source: BMC Plant Biol. 2010 Jun 28;10:132. doi: 10.1186/1471-2229-10-132 (PMC3017793; doi:10.1186/1471-2229-10-132)
Supplement: Additional file 2 — Tables S2: Distribution of QTLs in BC experiments. Distribution of the 67 significant QTLs and of the total 255 (including putative QTLs shown in parentheses) from the analysis of the 3 BC generations and shared among the 6 fiber trait categories, 3 generations and 26 chromosomes. [file 1471-2229-10-132-S2.DOC]

**Additional file - Table S2: Distribution of the 67 significant QTLs (LOD > permutation based threshold) and of the total 255 QTLs (including putative QTLs of LOD>2, shown in parentheses) from the analysis of the 3 BC generations.**

QTLs are shared among the 6 fiber trait categories, 3 generations and 26 chromosomes.

| **Category** | **Fineness** | **Length** | **Uniformity** | **Strength** | **Elongation** | **Color** |  | **Total** |
| --- | --- | --- | --- | --- | --- | --- | --- | --- |
| **Generation** |  |  |  |  |  |  |  |  |
| BC1 | 12 (26) | 4 (19) | 2 (6) | 4 (15) | 3 (10) | 3 (19) |  | **28 (95)** |
| BC2 | 5 (23) | 5 (20) | 2 (7) | 2 (8) | 4 (8) | 5 (17) |  | **23 (83)** |
| BC2S1 | 5 (22) | 4 (18) | 1 (6) | 1 (7) | 1 (8) | 4 (16) |  | **16 (77)** |
| **Chromosome** | |  |  |  |  |  |  |  |
| 1 | 0 (0) | 0 (0) | 1 (2) | 0 (0) | 0 (1) | 0 (2) |  | **1 (5)** |
| 2 | 1 (5) | 0 (1) | 0 (0) | 0 (0) | 0 (0) | 0 (1) |  | **1 (7)** |
| 3 | 3 (10) | 3 (14) | 0 (2) | 2 (4) | 0 (1) | 0 (0) |  | **8 (31)** |
| 4 | 1 (2) | 0 (0) | 0 (0) | 0 (0) | 0 (0) | 0 (0) |  | **1 (2)** |
| 5 | 1 (3) | 0 (1) | 0 (2) | 1 (3) | 0 (1) | 0 (0) |  | **2 (10)** |
| 6 | 3 (5) | 1 (4) | 0 (0) | 0 (0) | 0 (0) | 0 (2) |  | **4 (11)** |
| 7 | 0 (1) | 0 (0) | 0 (1) | 0 (1) | 0 (0) | 0 (0) |  | **0 (3)** |
| 8 | 3 (6) | 0 (0) | 0 (0) | 0 (0) | 0 (0) | 1 (9) |  | **4 (15)** |
| 9 | 0 (1) | 0 (0) | 0 (1) | 0 (0) | 1 (4) | 0 (2) |  | **1 (8)** |
| 10 | 1 (9) | 0 (1) | 0 (1) | 0 (0) | 1 (4) | 0 (3) |  | **2 (18)** |
| 11 | 0 (0) | 0 (0) | 0 (1) | 0 (7) | 0 (1) | 0 (0) |  | **0 (9)** |
| 12 | 0 (0) | 0 (1) | 0 (0) | 0 (0) | 0 (1) | 0 (2) |  | **0 (4)** |
| 13 | 0 (0) | 1 (5) | 0 (0) | 0 (1) | 0 (0) | 0 (2) |  | **1 (8)** |
| 14 | 0 (0) | 0 (1) | 0 (0) | 0 (1) | 0 (0) | 0 (0) |  | **0 (2)** |
| 15 | 0 (3) | 0 (0) | 0 (0) | 0 (0) | 0 (1) | 0 (0) |  | **0 (4)** |
| 16 | 0 (0) | 0 (0) | 1 (1) | 1 (1) | 0 (0) | 0 (2) |  | **2 (4)** |
| 17 | 0 (0) | 0 (0) | 0 (1) | 0 (0) | 0 (0) | 1 (3) |  | **1 (4)** |
| 18 | 2 (3) | 1 (3) | 2 (3) | 1 (2) | 0 (0) | 1 (2) |  | **7 (13)** |
| 19 | 3 (8) | 3 (8) | 0 (1) | 0 (0) | 3 (7) | 3 (6) |  | **12 (30)** |
| 20 | 0 (0) | 1 (3) | 1 (2) | 0 (0) | 1 (2) | 0 (0) |  | **3 (7)** |
| 21 | 0 (3) | 0 (0) | 0 (0) | 0 (0) | 0 (2) | 0 (0) |  | **0 (5)** |
| 22 | 1 (1) | 0 (1) | 0 (0) | 0 (0) | 1 (0) | 0 (0) |  | **2 (2)** |
| 23 | 0 (3) | 1 (6) | 0 (1) | 2 (9) | 1 (1) | 0 (0) |  | **4 (20)** |
| 24 | 2 (5) | 0 (0) | 0 (0) | 0 (0) | 0 (0) | 0 (2) |  | **2 (7)** |
| 25 | 1 (3) | 0 (2) | 0 (0) | 0 (1) | 0 (0) | 6 (13) |  | **7 (19)** |
| 26 | 0 (0) | 2 (6) | 0 (0) | 0 (0) | 0 (0) | 0 (1) |  | **2 (7)** |
| **Total** | **22 (71)** | **13 (57)** | **5 (19)** | **7 (30)** | **8 (26)** | **12 (52)** |  | **67 (255)** |
