# Supplementary material for: Meta-analysis of cotton fiber quality QTLs across diverse environments in a Gossypium hirsutum x G. barbadense RIL population
Source: BMC Plant Biol. 2010 Jun 28;10:132. doi: 10.1186/1471-2229-10-132 (PMC3017793; doi:10.1186/1471-2229-10-132)

Chromosome 1

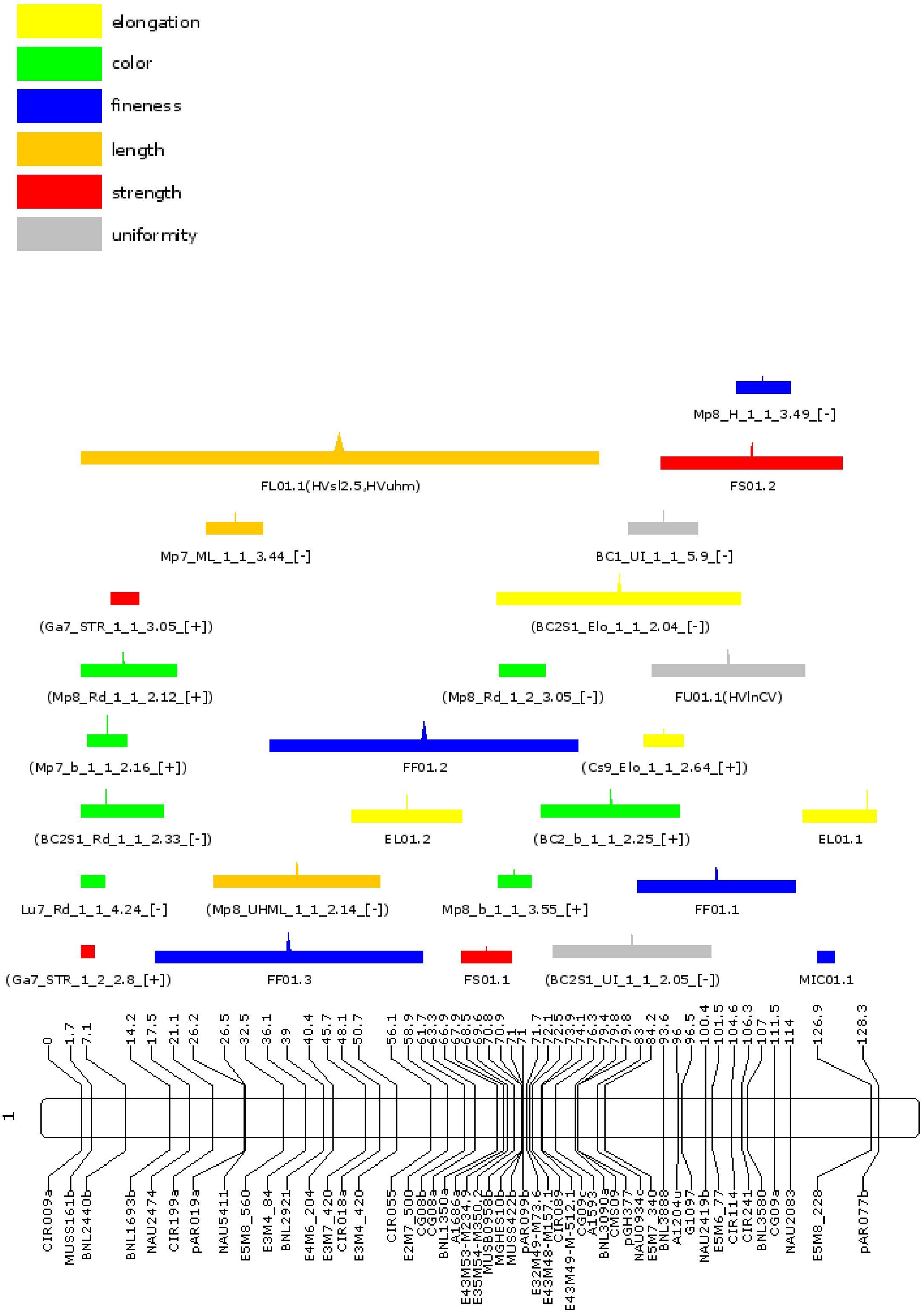

Chromosome 2

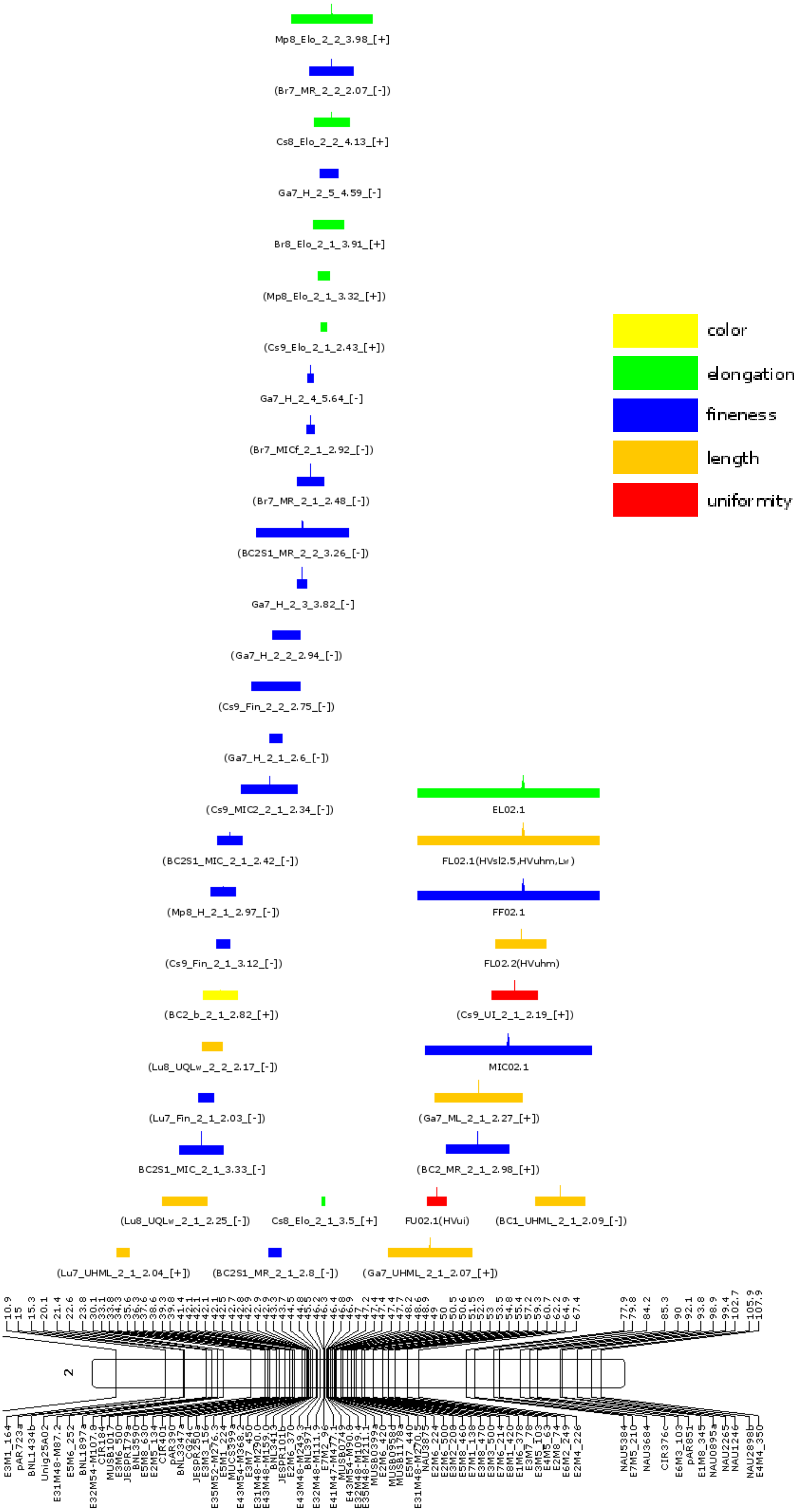

Chromosome 3

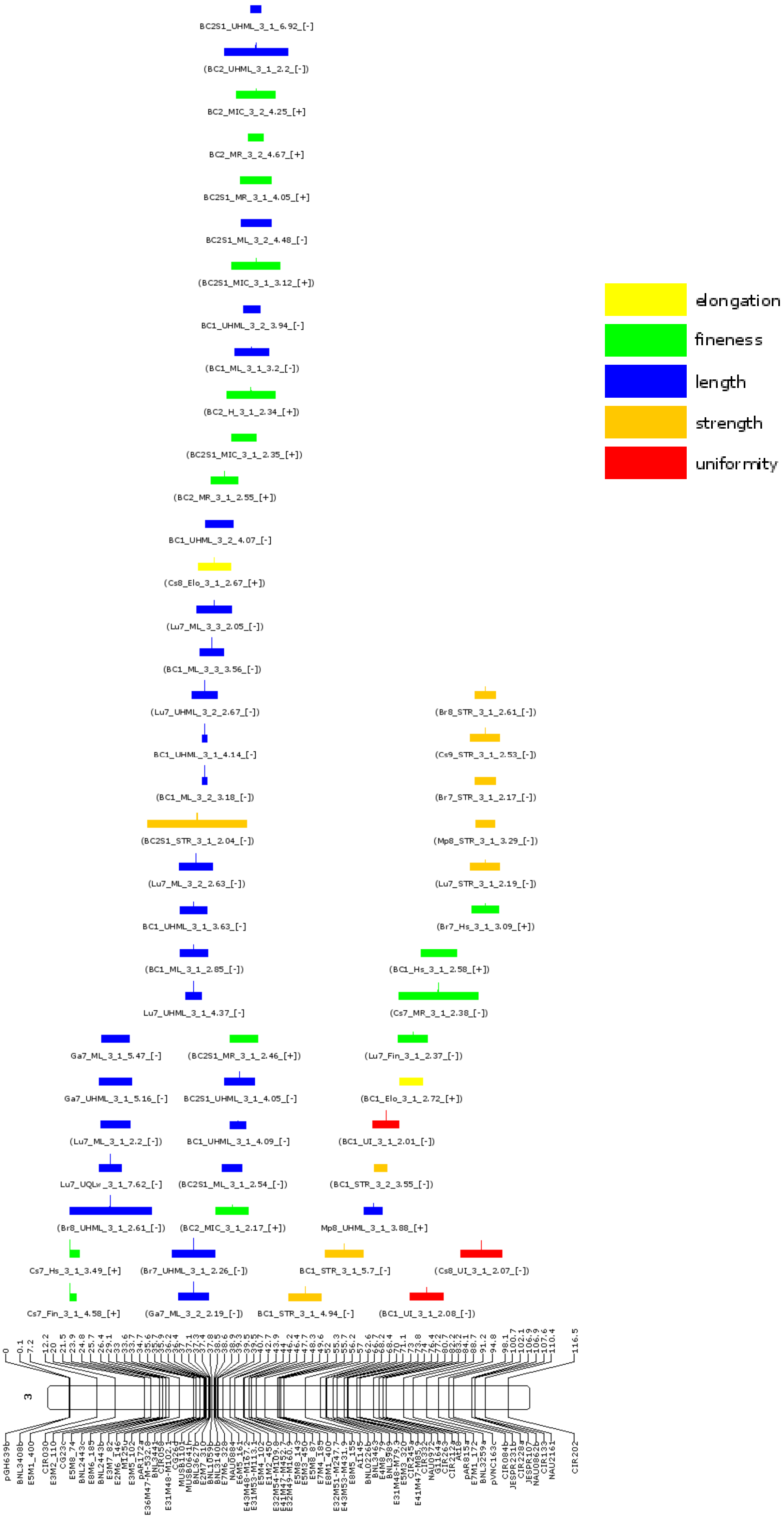

Chromosome 4

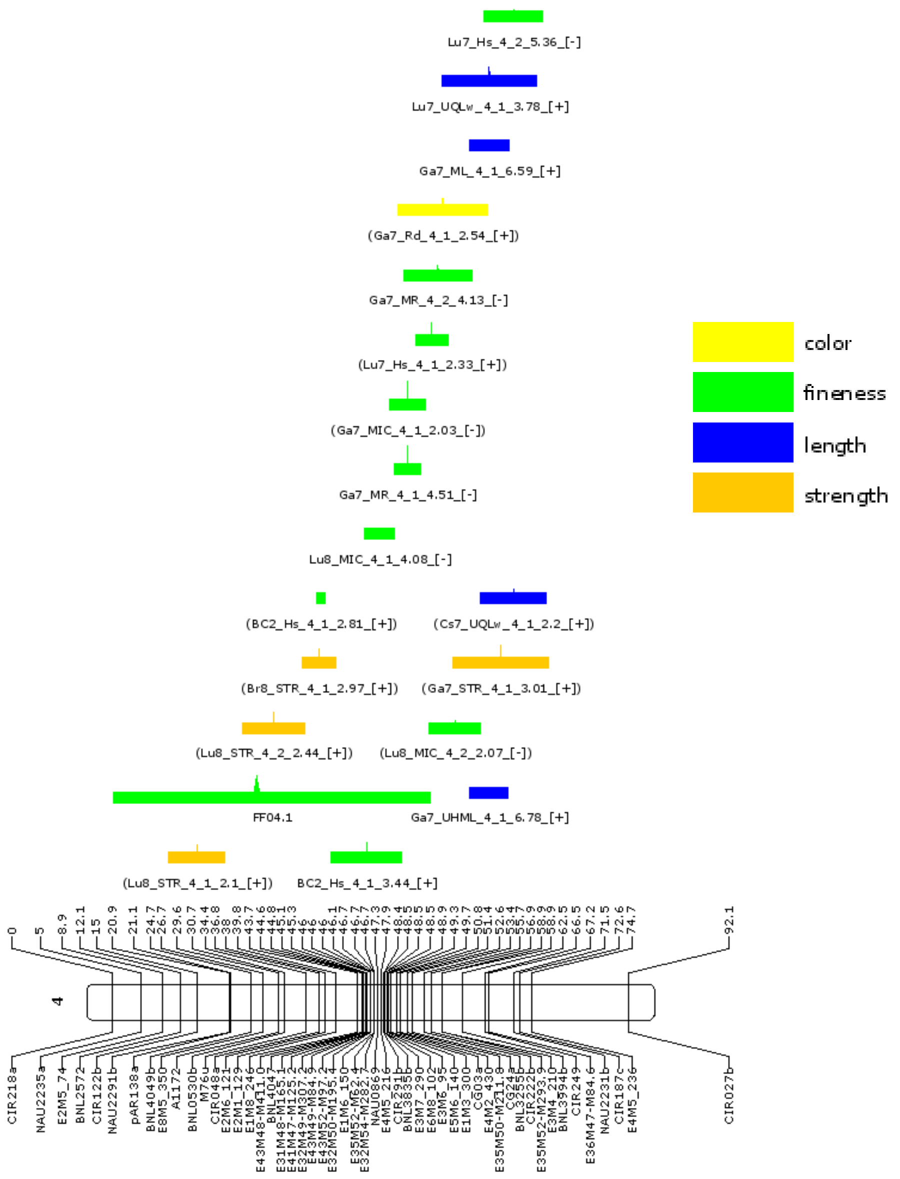

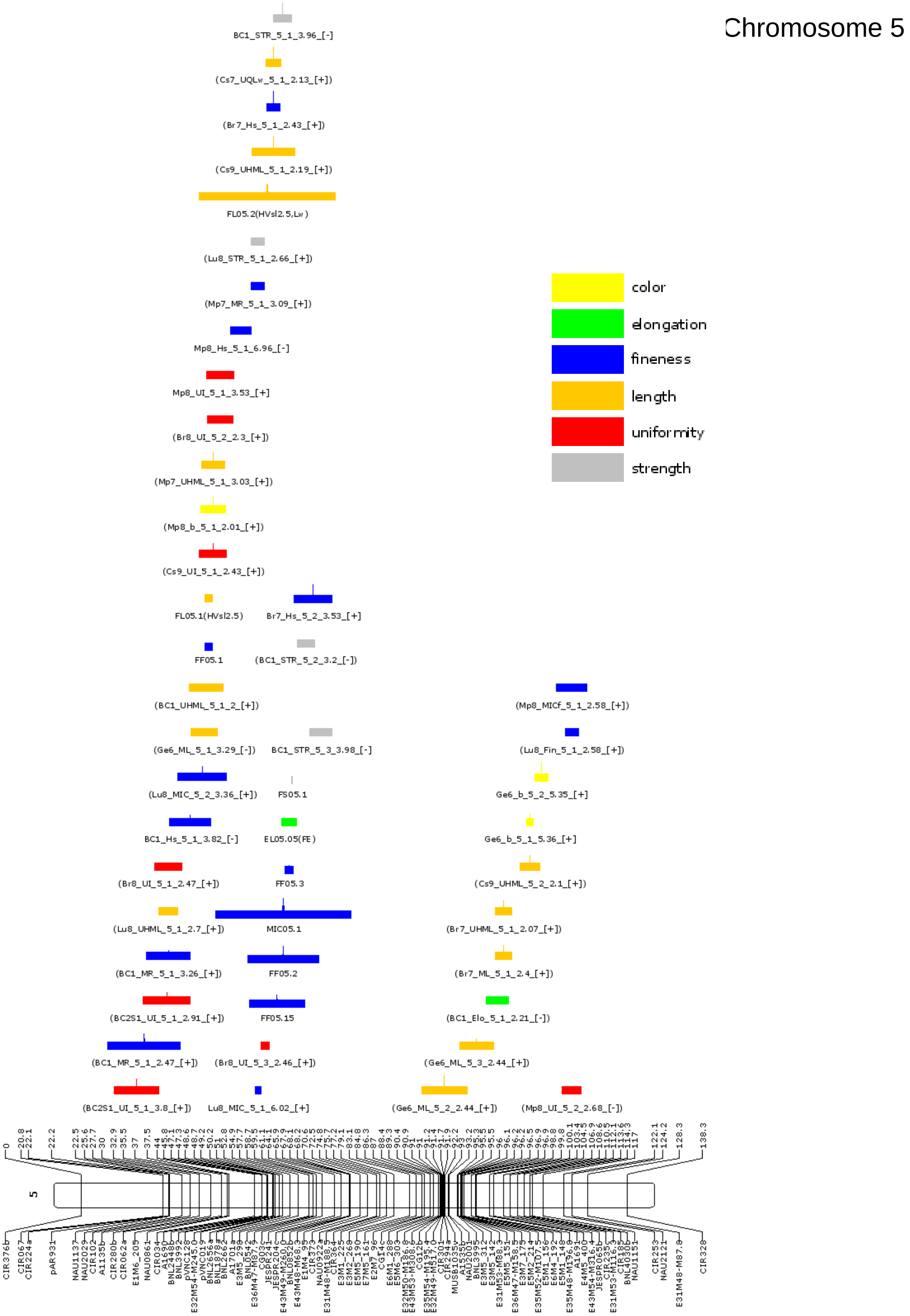
Chromosome 5
color
elongation
fineness
length
uniformity
strength
BC1_STR_5_1_3.96_[-]
(Cs7_UQLw_5_1_2.13_[+])
(Br7_Hs_5_1_2.43_[+])
(Cs9_UHML_5_1_2.19_[+])
FL05.2(HVsl2.5,Lw)
(Lu8_STR_5_1_2.66_[+])
(Mp7_MR_5_1_3.09_[+])
Mp8_Hs_5_1_6.96_[-]
Mp8_UI_5_1_3.53_[+]
(Br8_UI_5_2_2.3_[+])
(Mp7_UHML_5_1_3.03_[+])
(Mp8_b_5_1_2.01_[+])
(Cs9_UI_5_1_2.43_[+])
FL05.1(HVsl2.5)
Br7_Hs_5_2_3.53_[+]
FF05.1
(BC1_STR_5_2_3.2_[-])
(BC1_UHML_5_1_2_[+])
(Mp8_MICf_5_1_2.58_[+])
(Ge6_ML_5_1_3.29_[-])
BC1_STR_5_3_3.98_[-]
(Lu8_Fin_5_1_2.58_[+])
(Lu8_MIC_5_2_3.36_[+])
FS05.1
Ge6_b_5_2_5.35_[+]
BC1_Hs_5_1_3.82_[-]
EL05.05(FE)
Ge6_b_5_1_5.36_[+]
(Br8_UI_5_1_2.47_[+])
FF05.3
(Cs9_UHML_5_2_2.1_[+])
(Lu8_UHML_5_1_2.7_[+])
MIC05.1
(Br7_UHML_5_1_2.07_[+])
(BC1_MR_5_1_3.26_[+])
FF05.2
(Br7_ML_5_1_2.4_[+])
(BC2S1_UI_5_1_2.91_[+])
FF05.15
(BC1_Elo_5_1_2.21_[-])
(BC1_MR_5_1_2.47_[+])
(Br8_UI_5_3_2.46_[+])
(Ge6_ML_5_3_2.44_[+])
(BC2S1_UI_5_1_3.8_[+])
Lu8_MIC_5_1_6.02_[+]
(Ge6_ML_5_2_2.44_[+])
(Mp8_UI_5_2_2.68_[-])
5

Chromosome 6

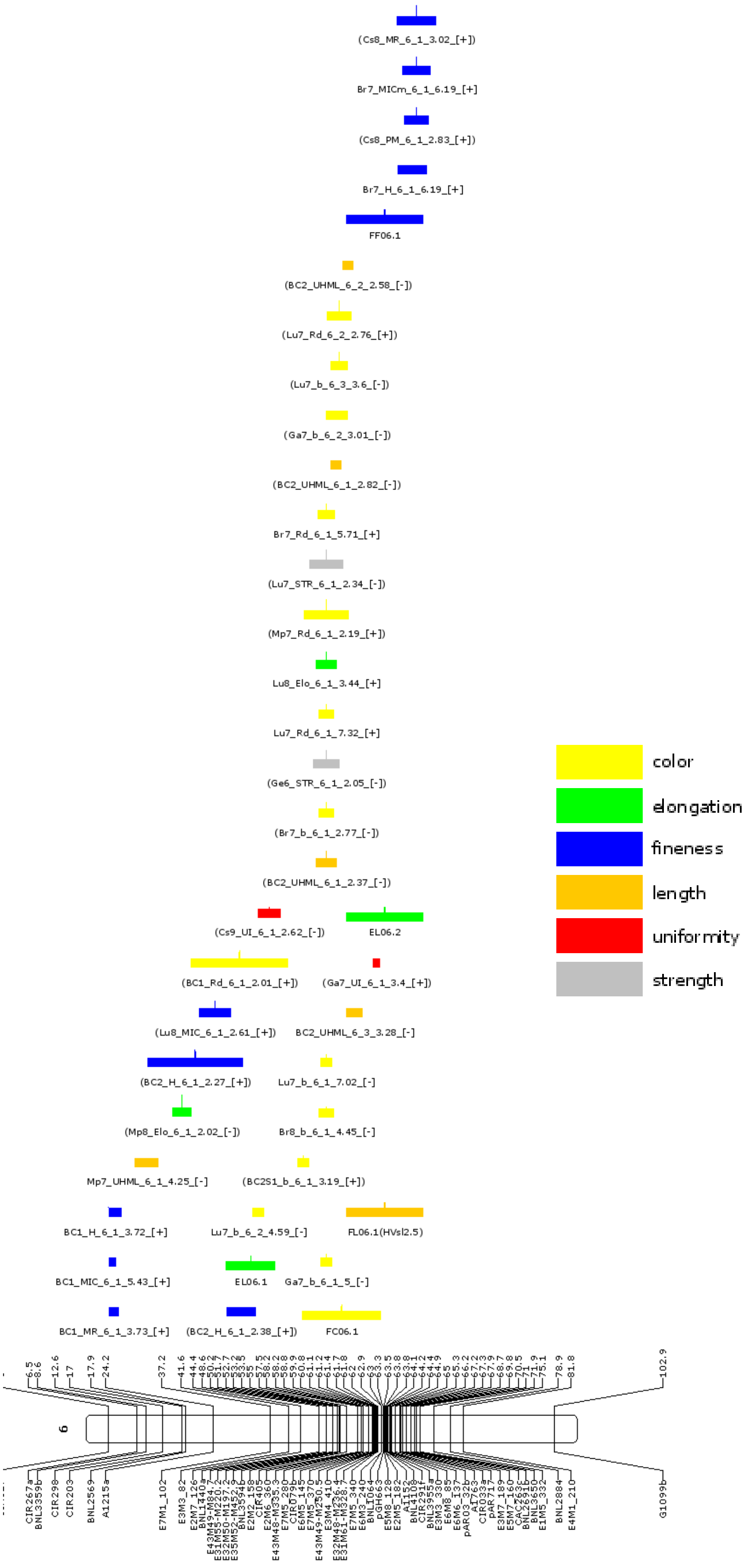

Chromosome 7

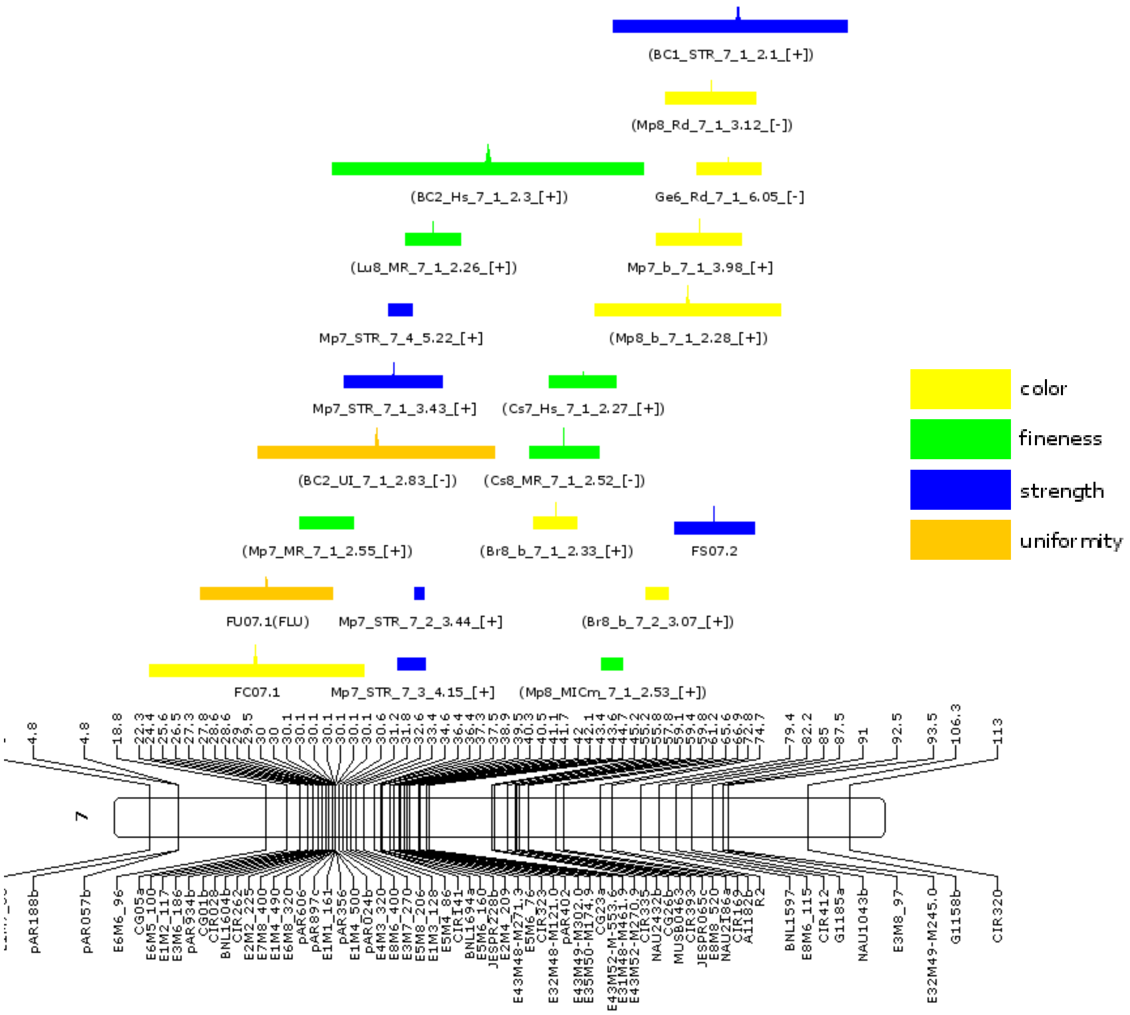

Chromosome 8

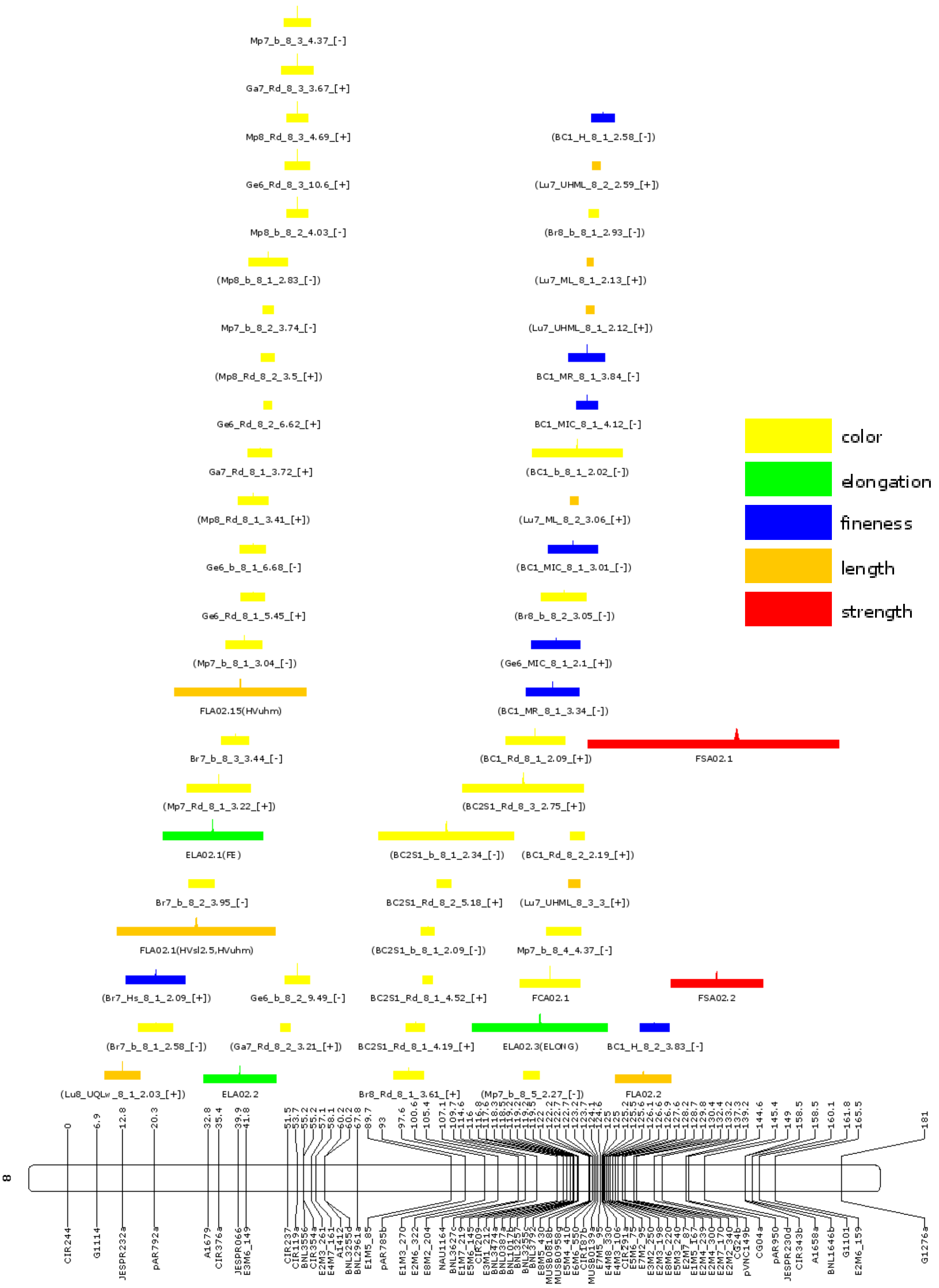

Chromosome 9

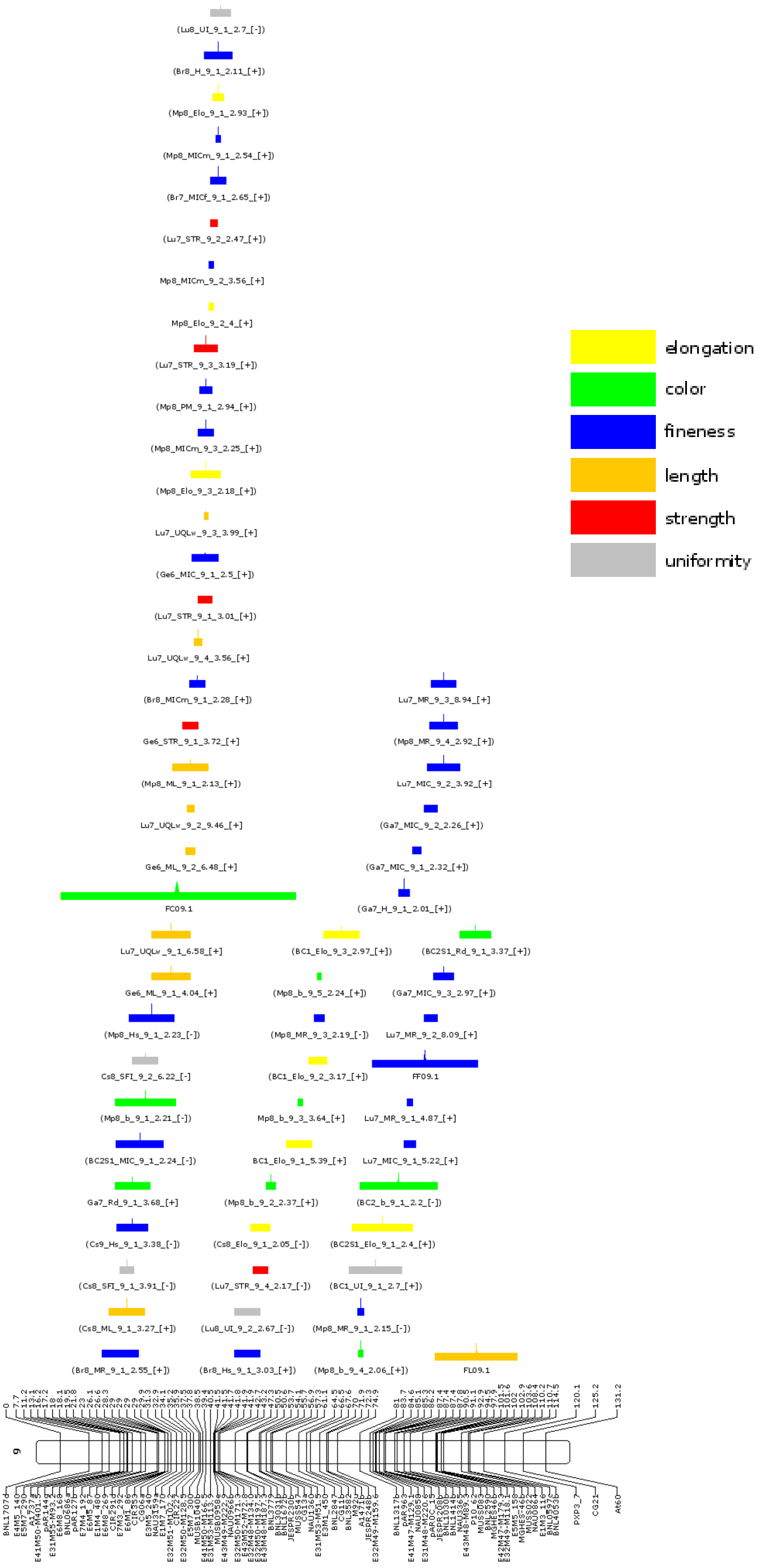

Chromosome 10

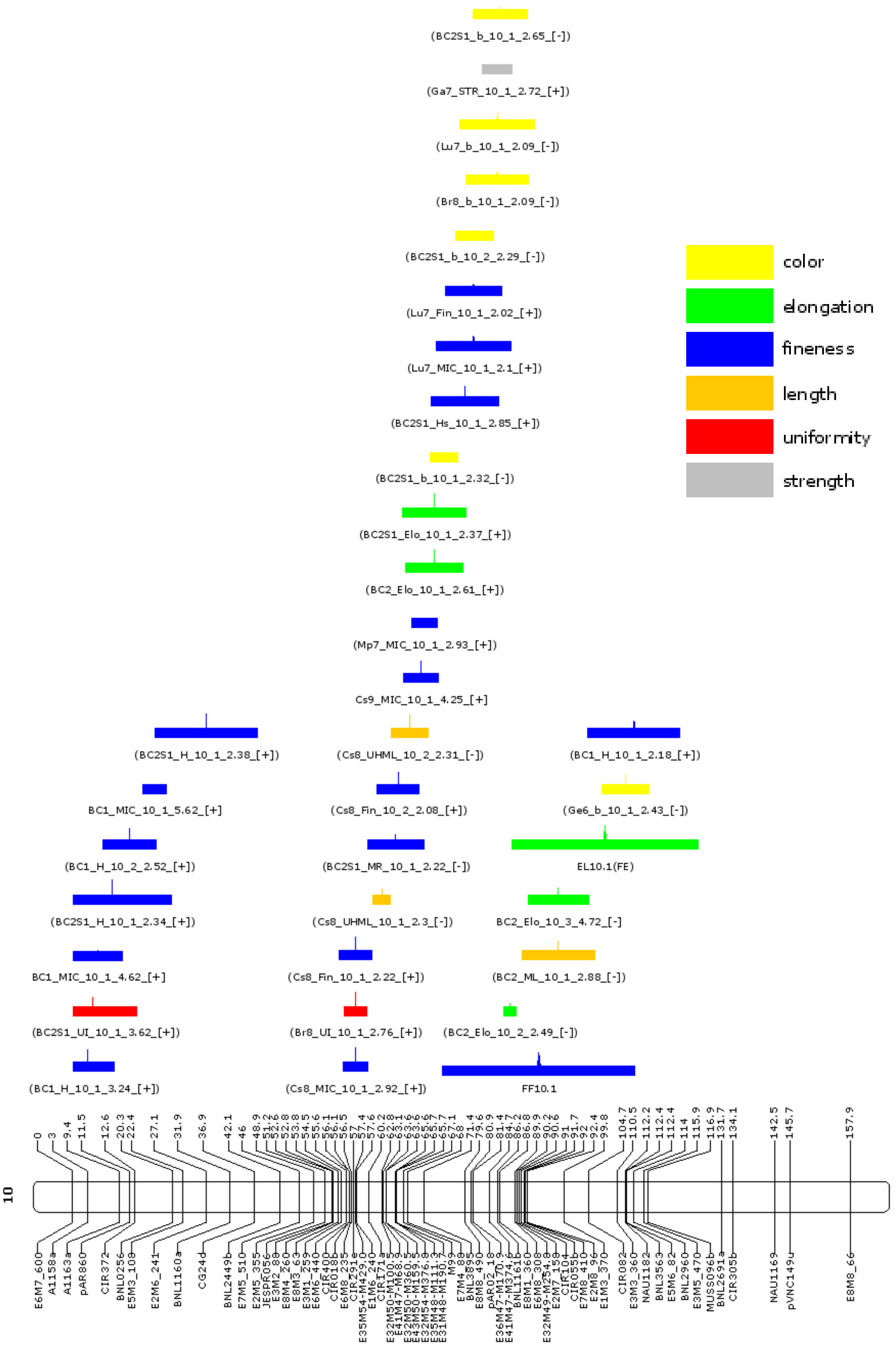

Chromosome 11

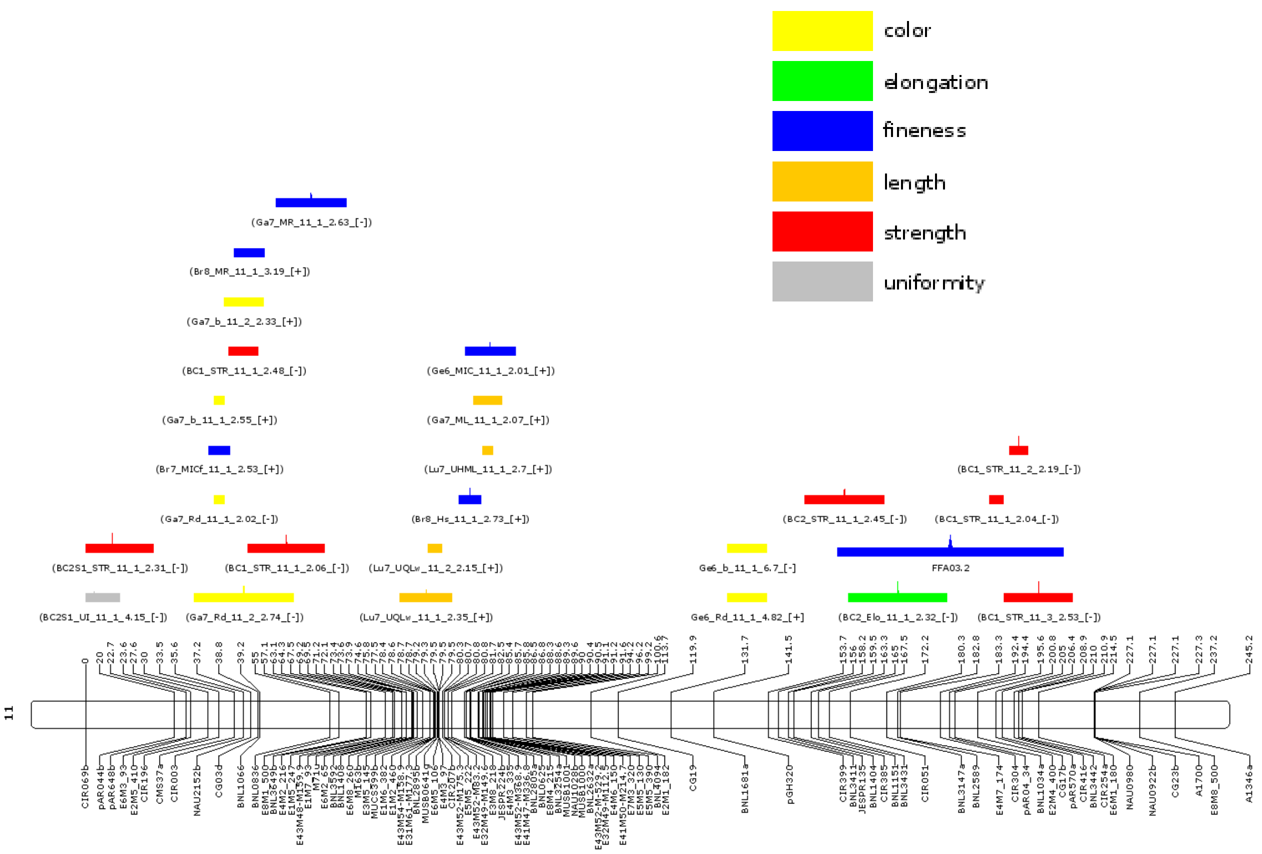

Chromosome 12

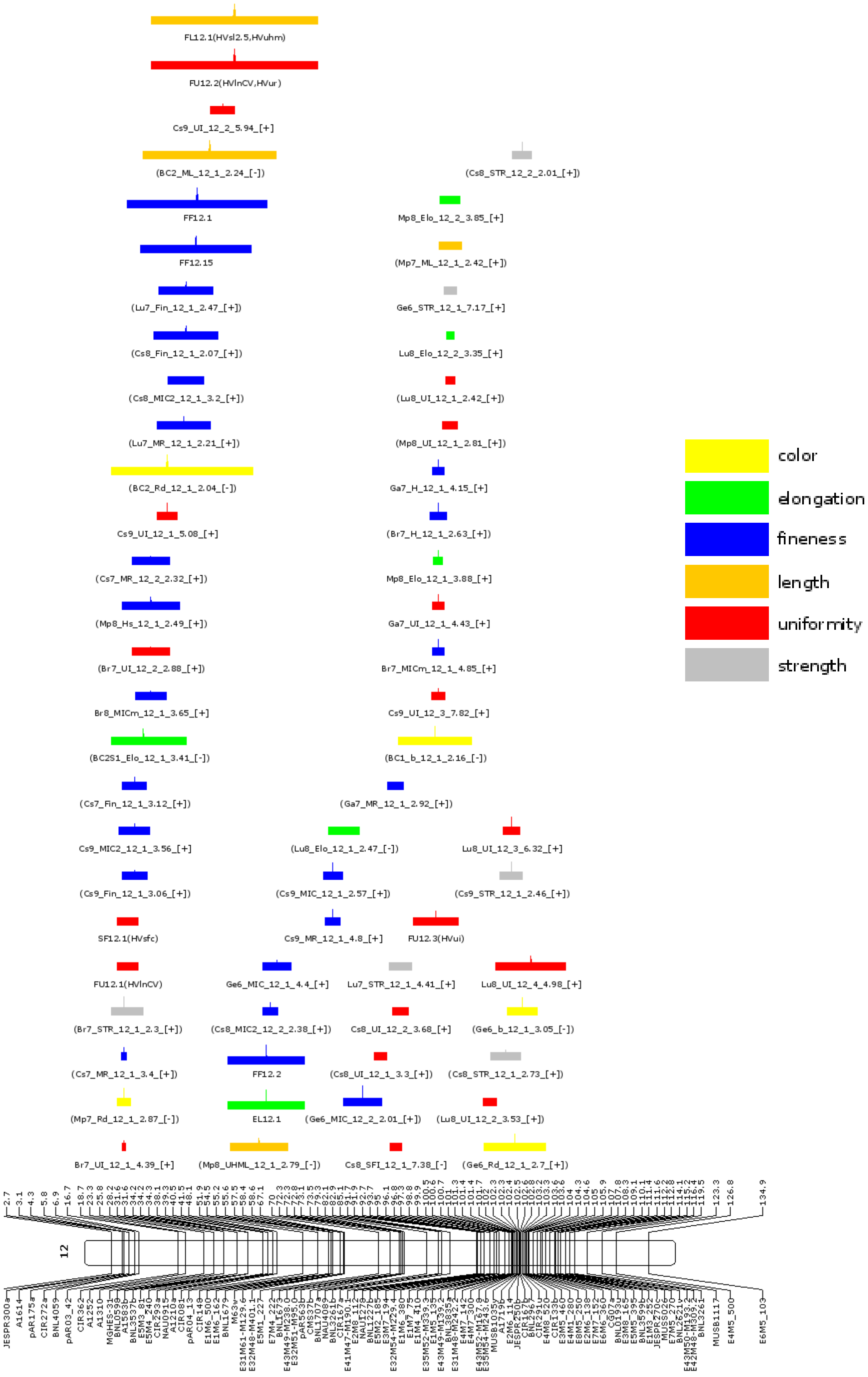

Chromosome 13

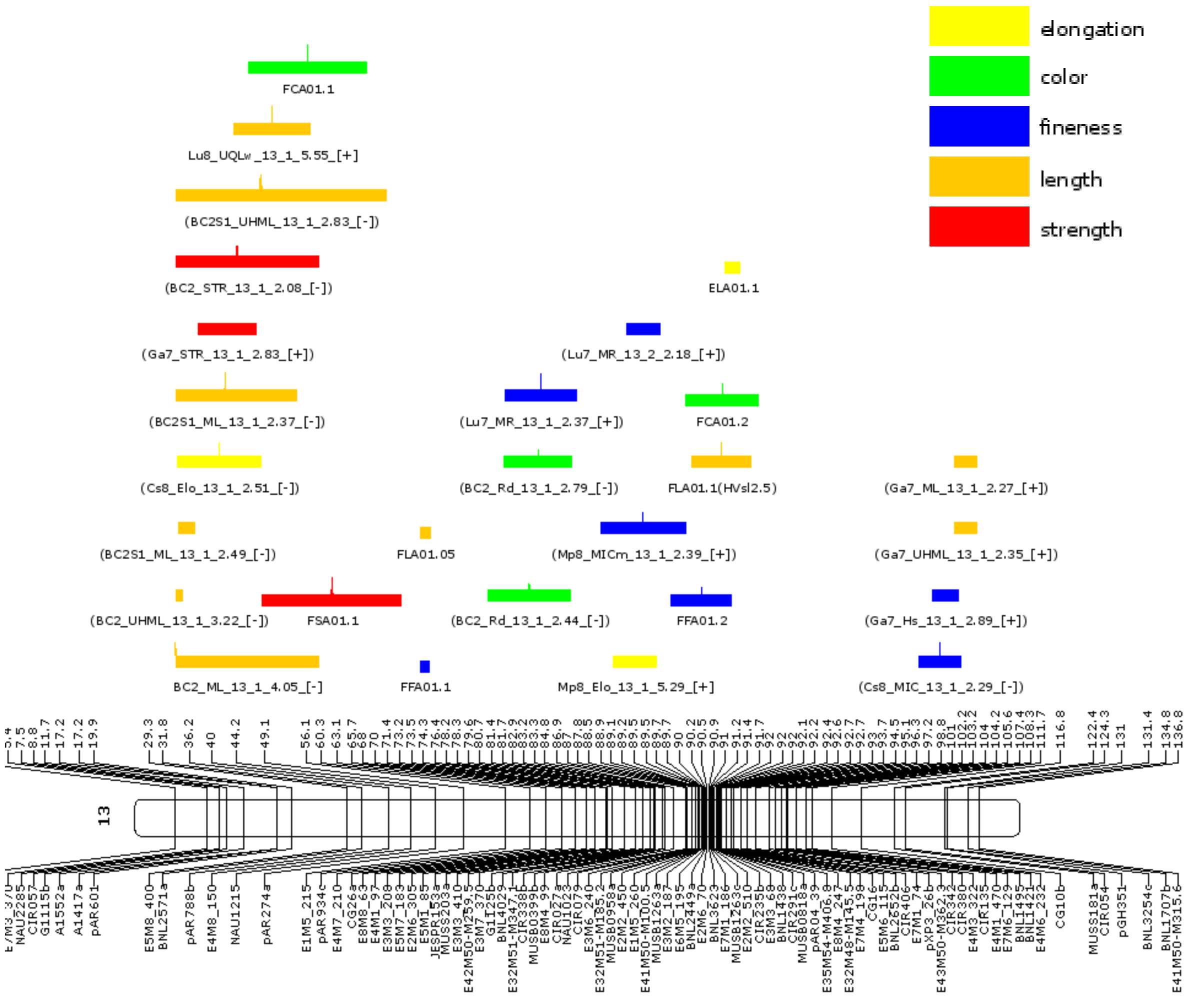

Chromosome 14

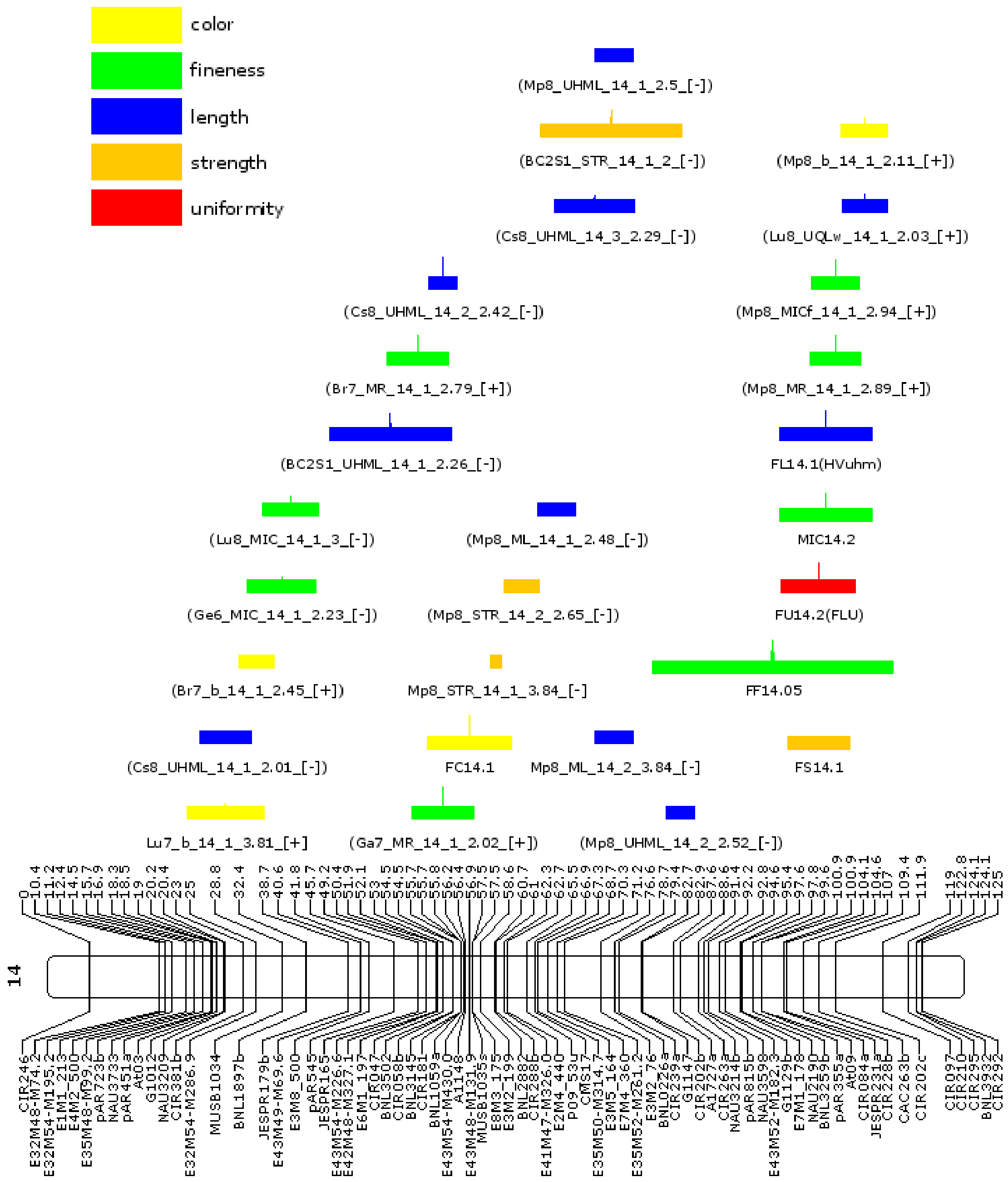

Chromosome 15

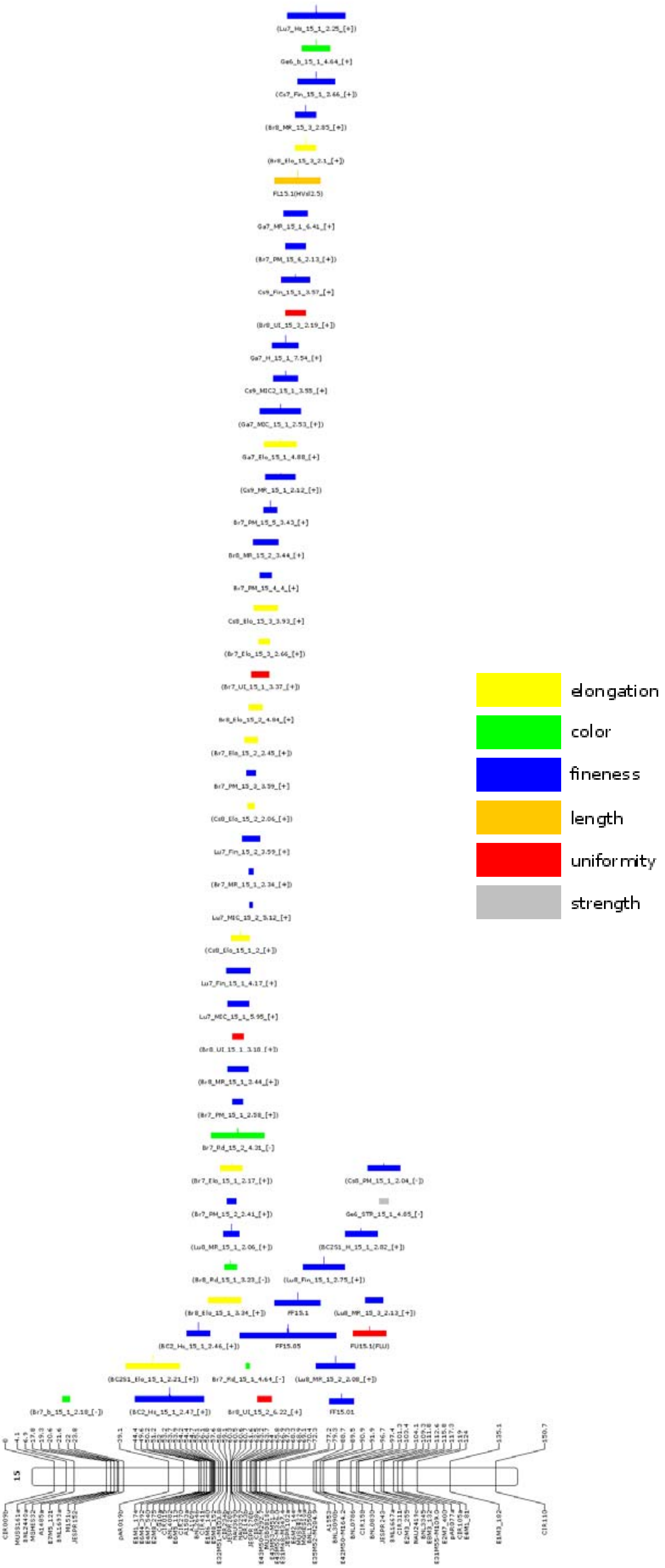

Chromosome 16

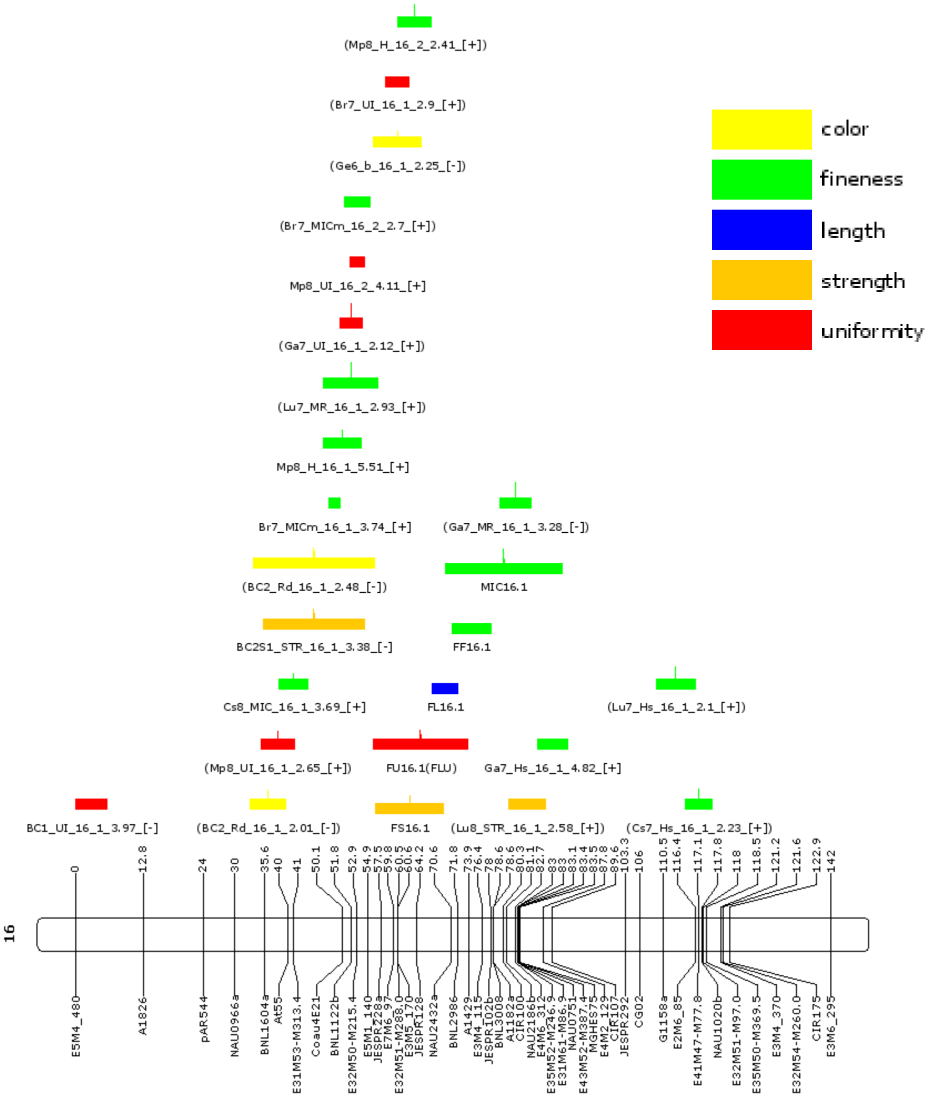

Chromosome 17

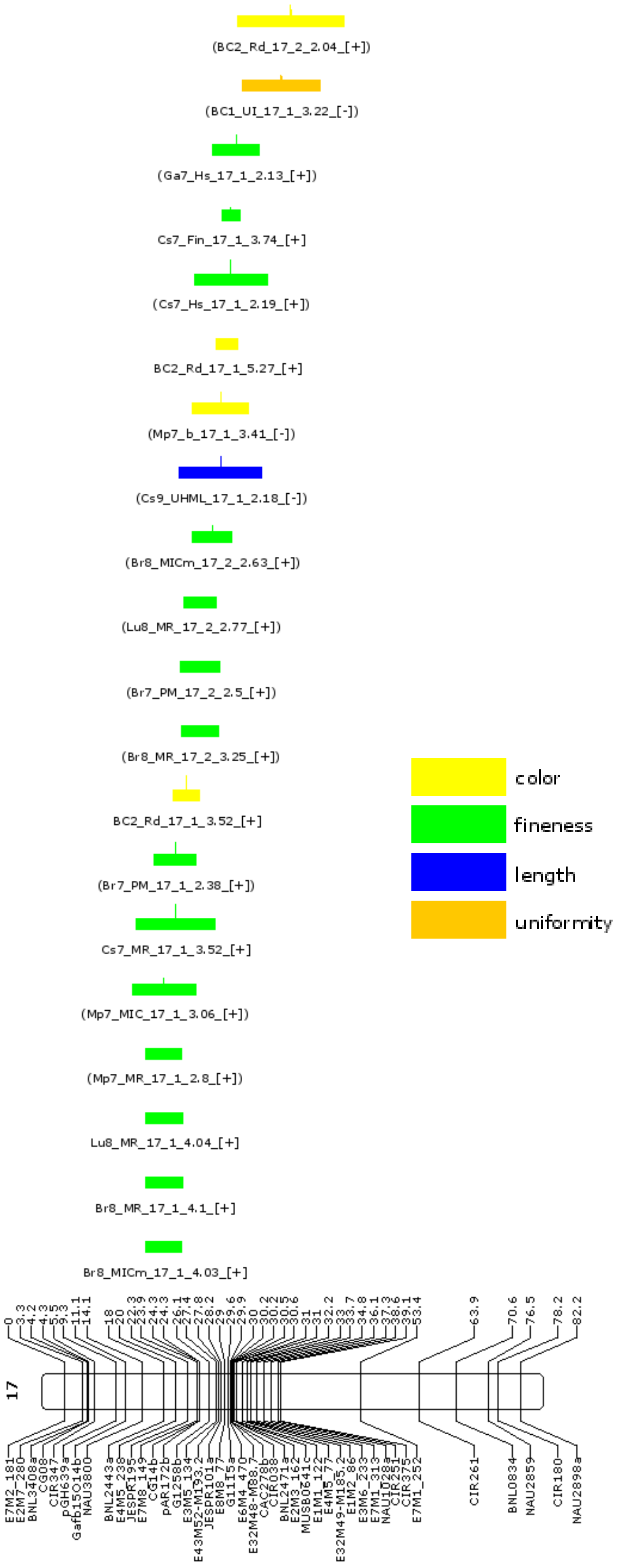

Chromosome 18

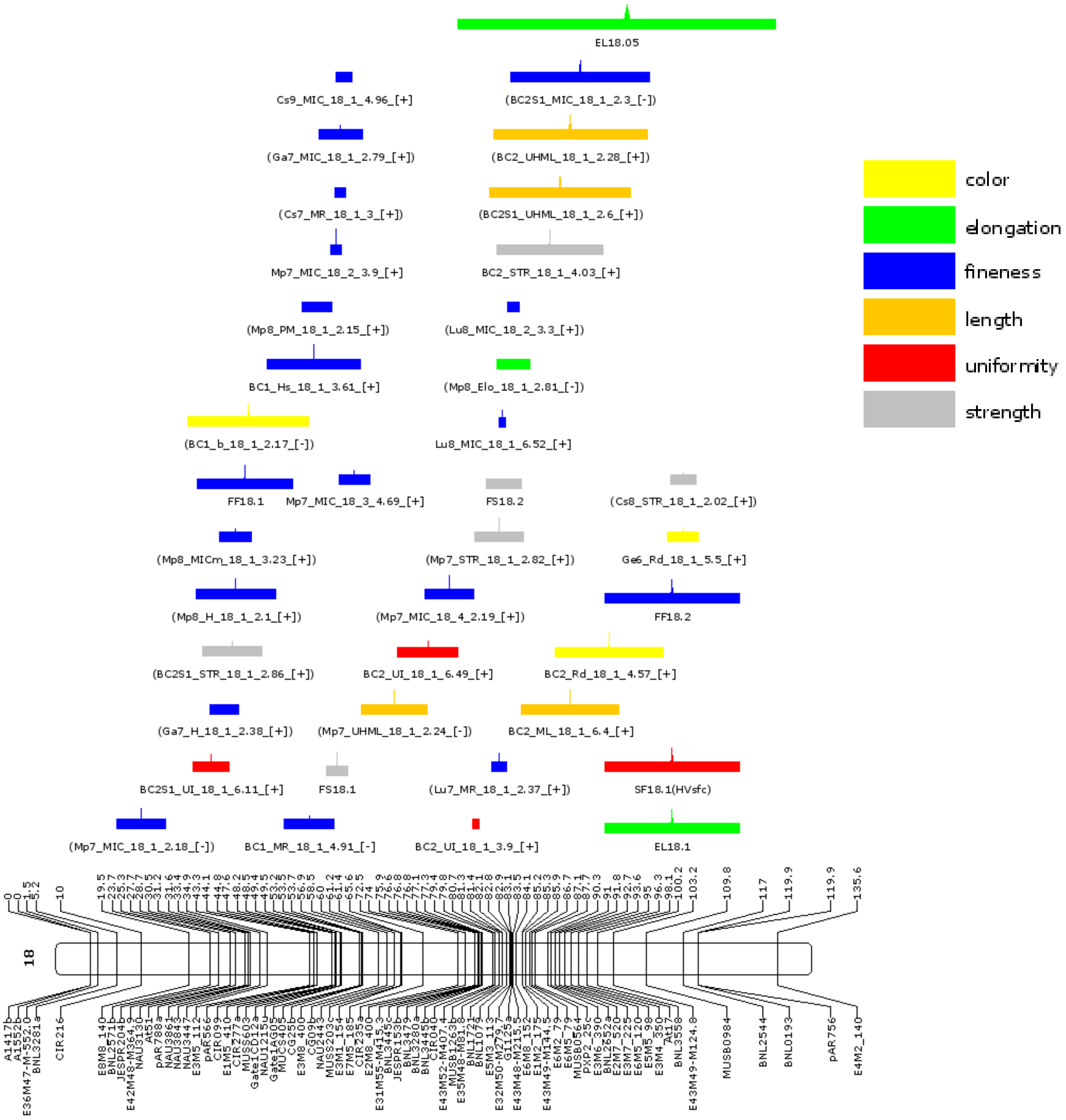

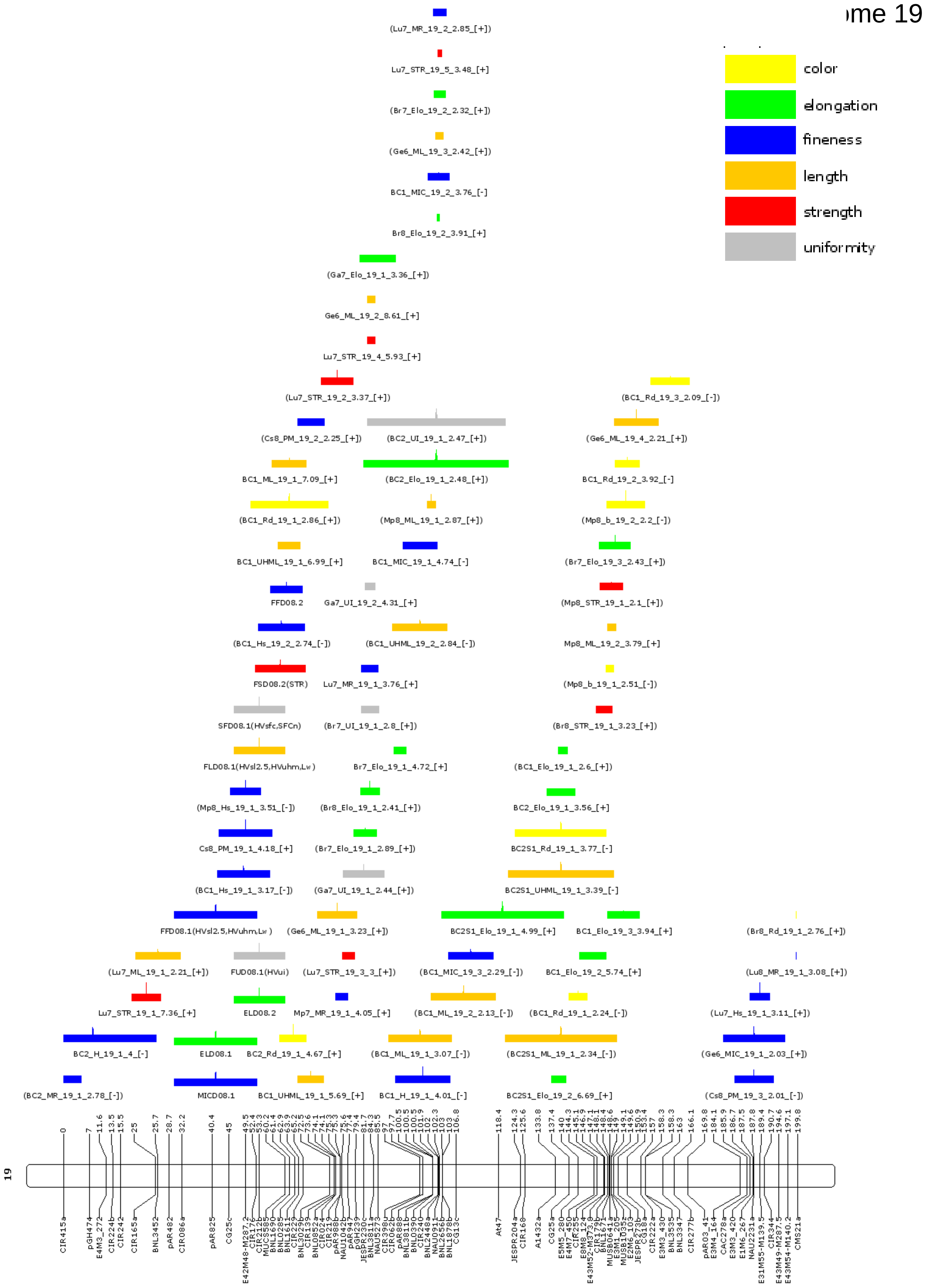
ome 19
color
elongation
fineness
length
strength
uniformity
(Lu7_MR_19_2_2.85_[+])
Lu7_STR_19_5_3.48_[+]
(Br7_Elo_19_2_2.32_[+])
(Ge6_ML_19_3_2.42_[+])
BC1_MIC_19_2_3.76_[-]
Br8_Elo_19_2_3.91_[+]
(Ga7_Elo_19_1_3.36_[+])
Ge6_ML_19_2_8.61_[+]
Lu7_STR_19_4_5.93_[+]
(Lu7_STR_19_2_3.37_[+])
(BC1_Rd_19_3_2.09_[-])
(Cs8_PM_19_2_2.25_[+])
(BC2_UI_19_1_2.47_[+])
(Ge6_ML_19_4_2.21_[+])
BC1_ML_19_1_7.09_[+]
(BC2_Elo_19_1_2.48_[+])
BC1_Rd_19_2_3.92_[-]
(BC1_Rd_19_1_2.86_[+])
(Mp8_ML_19_1_2.87_[+])
(Mp8_b_19_2_2.2_[-])
BC1_UHML_19_1_6.99_[+]
BC1_MIC_19_1_4.74_[-]
(Br7_Elo_19_3_2.43_[+])
FFD08.2
Ga7_UI_19_2_4.31_[+]
(Mp8_STR_19_1_2.1_[+])
(BC1_Hs_19_2_2.74_[-])
(BC1_UHML_19_2_2.84_[-])
Mp8_ML_19_2_3.79_[+]
FSD08.2(STR)
Lu7_MR_19_1_3.76_[+]
(Mp8_b_19_1_2.51_[-])
SFD08.1(HVsfc,SFCn)
(Br7_UI_19_1_2.8_[+])
(Br8_STR_19_1_3.23_[+])
FLD08.1(HVsl2.5,HVuhm,Lw)
Br7_Elo_19_1_4.72_[+]
(BC1_Elo_19_1_2.6_[+])
(Mp8_Hs_19_1_3.51_[-])
(Br8_Elo_19_1_2.41_[+])
BC2_Elo_19_1_3.56_[+]
Cs8_PM_19_1_4.18_[+]
(Br7_Elo_19_1_2.89_[+])
BC2S1_Rd_19_1_3.77_[-]
(BC1_Hs_19_1_3.17_[-])
(Ga7_UI_19_1_2.44_[+])
BC2S1_UHML_19_1_3.39_[-]
FFD08.1(HVsl2.5,HVuhm,Lw)
(Ge6_ML_19_1_3.23_[+])
BC2S1_Elo_19_1_4.99_[+]
BC1_Elo_19_3_3.94_[+]
(Br8_Rd_19_1_2.76_[+])
(Lu7_ML_19_1_2.21_[+])
FUD08.1(HVui)
(Lu7_STR_19_3_3_[+])
(BC1_MIC_19_3_2.29_[-])
BC1_Elo_19_2_5.74_[+]
(Lu8_MR_19_1_3.08_[+])
Lu7_STR_19_1_7.36_[+]
ELD08.2
Mp7_MR_19_1_4.05_[+]
(BC1_ML_19_2_2.13_[-])
(BC1_Rd_19_1_2.24_[-])
(Lu7_Hs_19_1_3.11_[+])
BC2_H_19_1_4_[-]
ELD08.1
BC2_Rd_19_1_4.67_[+]
(BC1_ML_19_1_3.07_[-])
(BC2S1_ML_19_1_2.34_[-])
(Ge6_MIC_19_1_2.03_[+])
(BC2_MR_19_1_2.78_[-])
MICD08.1
BC1_UHML_19_1_5.69_[+]
BC1_H_19_1_4.01_[-]
BC2S1_Elo_19_2_6.69_[+]
(Cs8_PM_19_3_2.01_[-])
19

Chromosome 20

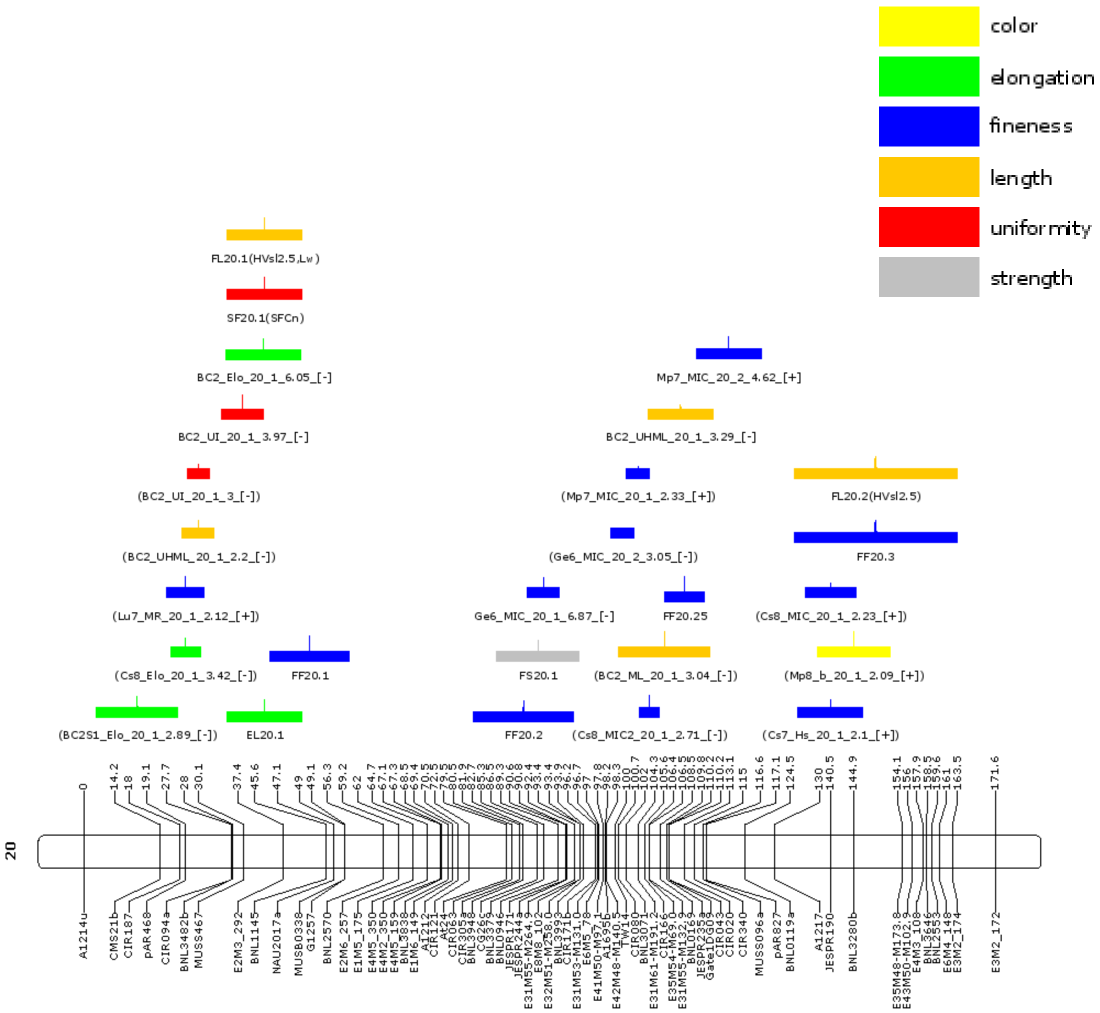

nosome 21

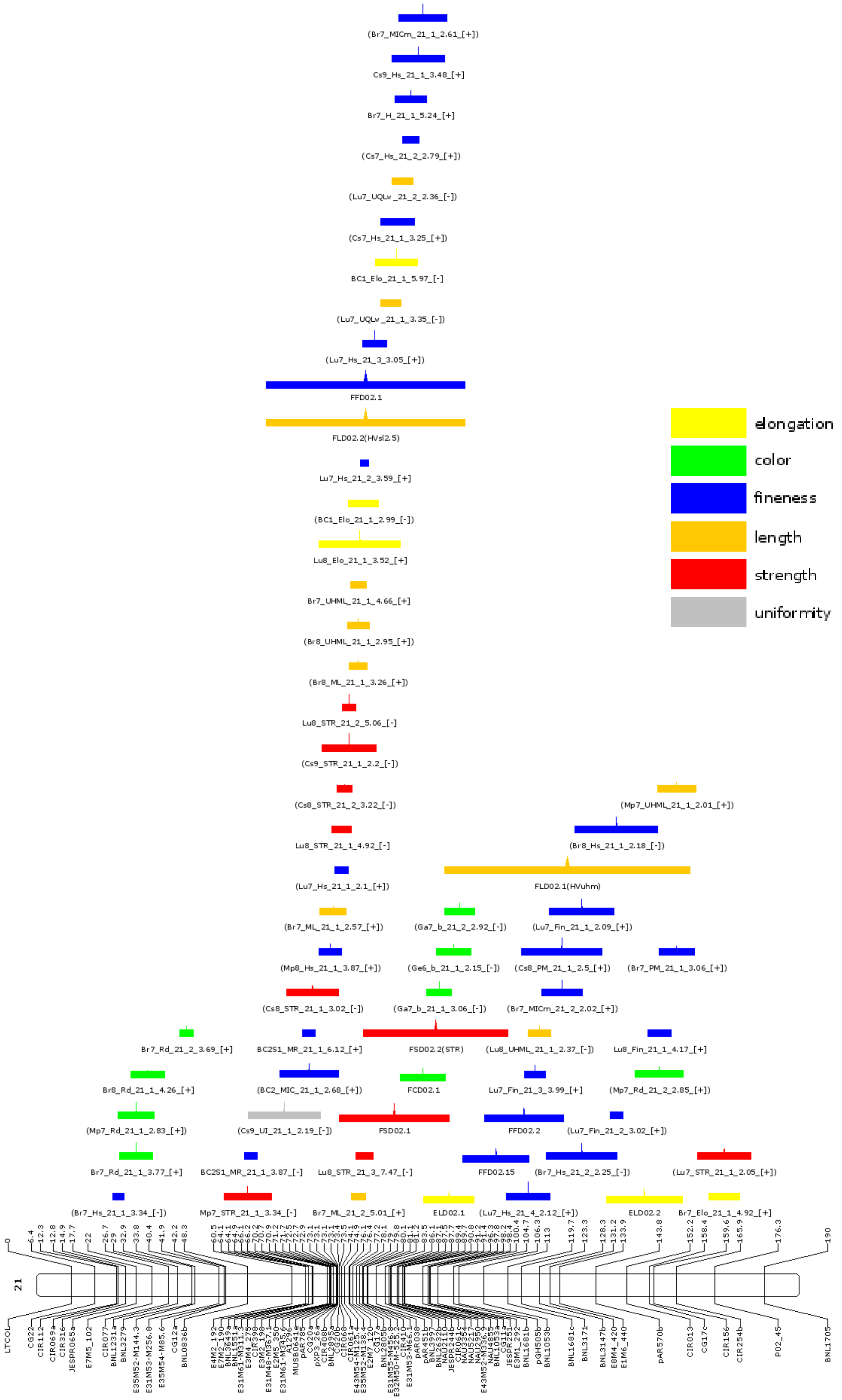
(Br7_MICm_21_1_2.61_[+])
Cs9_Hs_21_1_3.48_[+]
Br7_H_21_1_5.24_[+]
(Cs7_Hs_21_2_2.79_[+])
(Lu7_UQLw_21_2_2.36_[-])
(Cs7_Hs_21_1_3.25_[+])
BC1_Elo_21_1_5.97_[-]
(Lu7_UQLw_21_1_3.35_[-])
(Lu7_Hs_21_3_3.05_[+])
FFD02.1
FLD02.2(HVsl2.5)
Lu7_Hs_21_2_3.59_[+]
(BC1_Elo_21_1_2.99_[-])
Lu8_Elo_21_1_3.52_[+]
Br7_UHML_21_1_4.66_[+]
(Br8_UHML_21_1_2.95_[+])
(Br8_ML_21_1_3.26_[+])
Lu8_STR_21_2_5.06_[-]
(Cs9_STR_21_1_2.2_[-])
(Cs8_STR_21_2_3.22_[-])
(Mp7_UHML_21_1_2.01_[+])
Lu8_STR_21_1_4.92_[-]
(Br8_Hs_21_1_2.18_[-])
(Lu7_Hs_21_1_2.1_[+])
FLD02.1(HVuhm)
(Br7_ML_21_1_2.57_[+])
(Ga7_b_21_2_2.92_[-])
(Lu7_Fin_21_1_2.09_[+])
(Mp8_Hs_21_1_3.87_[+])
(Ge6_b_21_1_2.15_[-])
(Cs8_PM_21_1_2.5_[+])
(Br7_PM_21_1_3.06_[+])
(Cs8_STR_21_1_3.02_[-])
(Ga7_b_21_1_3.06_[-])
(Br7_MICm_21_2_2.02_[+])
Br7_Rd_21_2_3.69_[+]
BC2S1_MR_21_1_6.12_[+]
FSD02.2(STR)
(Lu8_UHML_21_1_2.37_[-])
Lu8_Fin_21_1_4.17_[+]
Br8_Rd_21_1_4.26_[+]
(BC2_MIC_21_1_2.68_[+])
FCD02.1
Lu7_Fin_21_3_3.99_[+]
(Mp7_Rd_21_2_2.85_[+])
(Mp7_Rd_21_1_2.83_[+])
(Cs9_UI_21_1_2.19_[-])
FSD02.1
FFD02.2
(Lu7_Fin_21_2_3.02_[+])
Br7_Rd_21_1_3.77_[+]
BC2S1_MR_21_1_3.87_[-]
Lu8_STR_21_3_7.47_[-]
FFD02.15
(Br7_Hs_21_2_2.25_[-])
(Lu7_STR_21_1_2.05_[+])
(Br7_Hs_21_1_3.34_[-])
Mp7_STR_21_1_3.34_[-]
Br7_ML_21_2_5.01_[+]
ELD02.1
(Lu7_Hs_21_4_2.12_[+])
ELD02.2
Br7_Elo_21_1_4.92_[+]
elongation
color
fineness
length
strength
uniformity
21

Chromosome 22

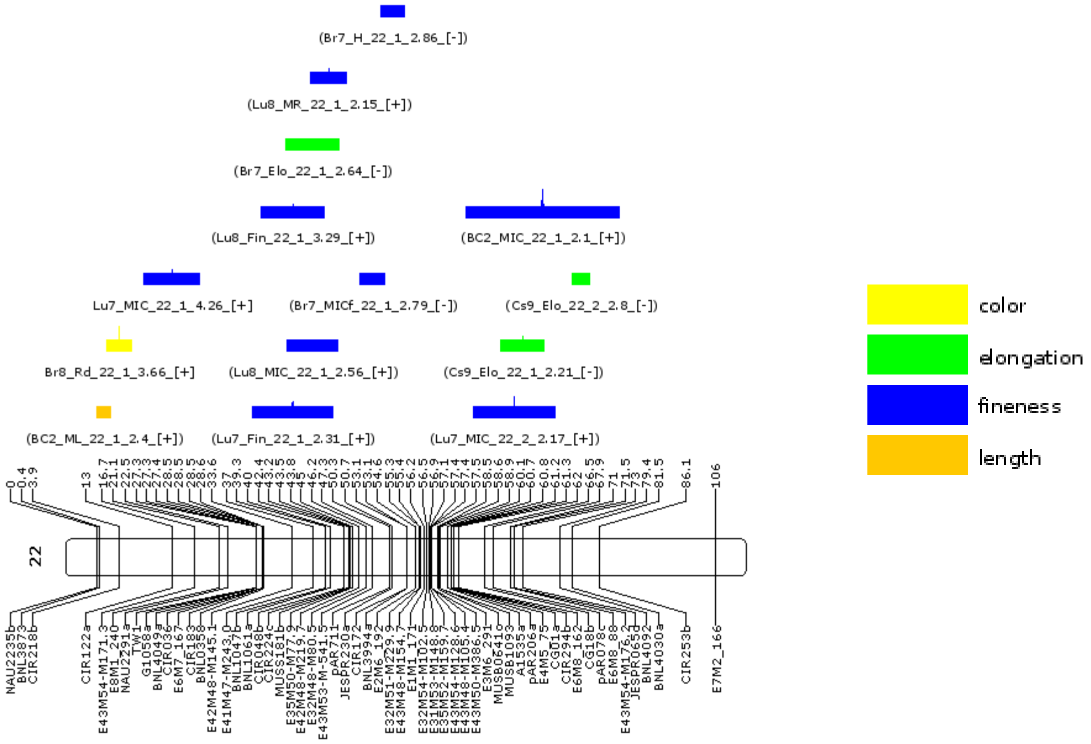

Chromosome 23

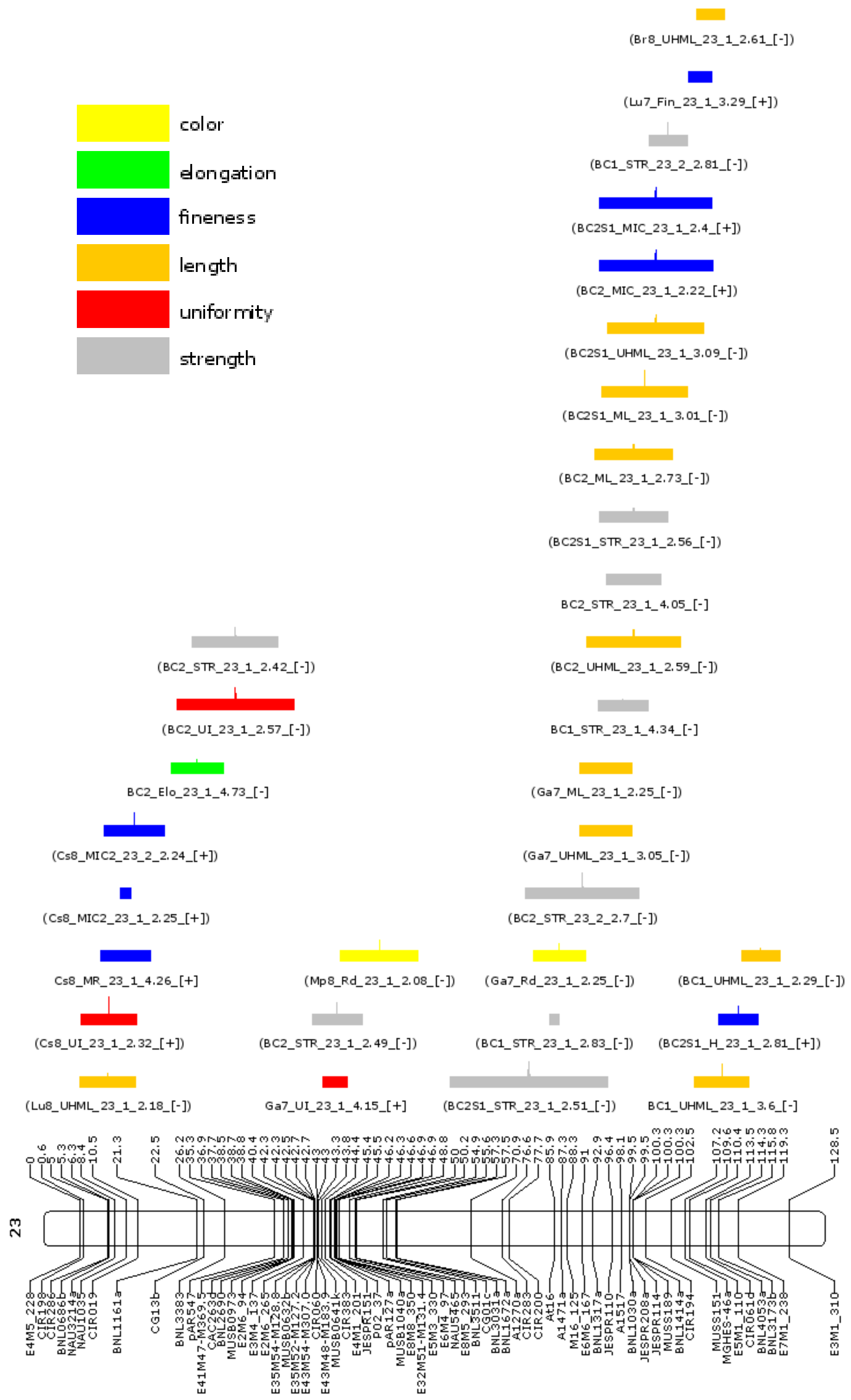

Chromosome 24

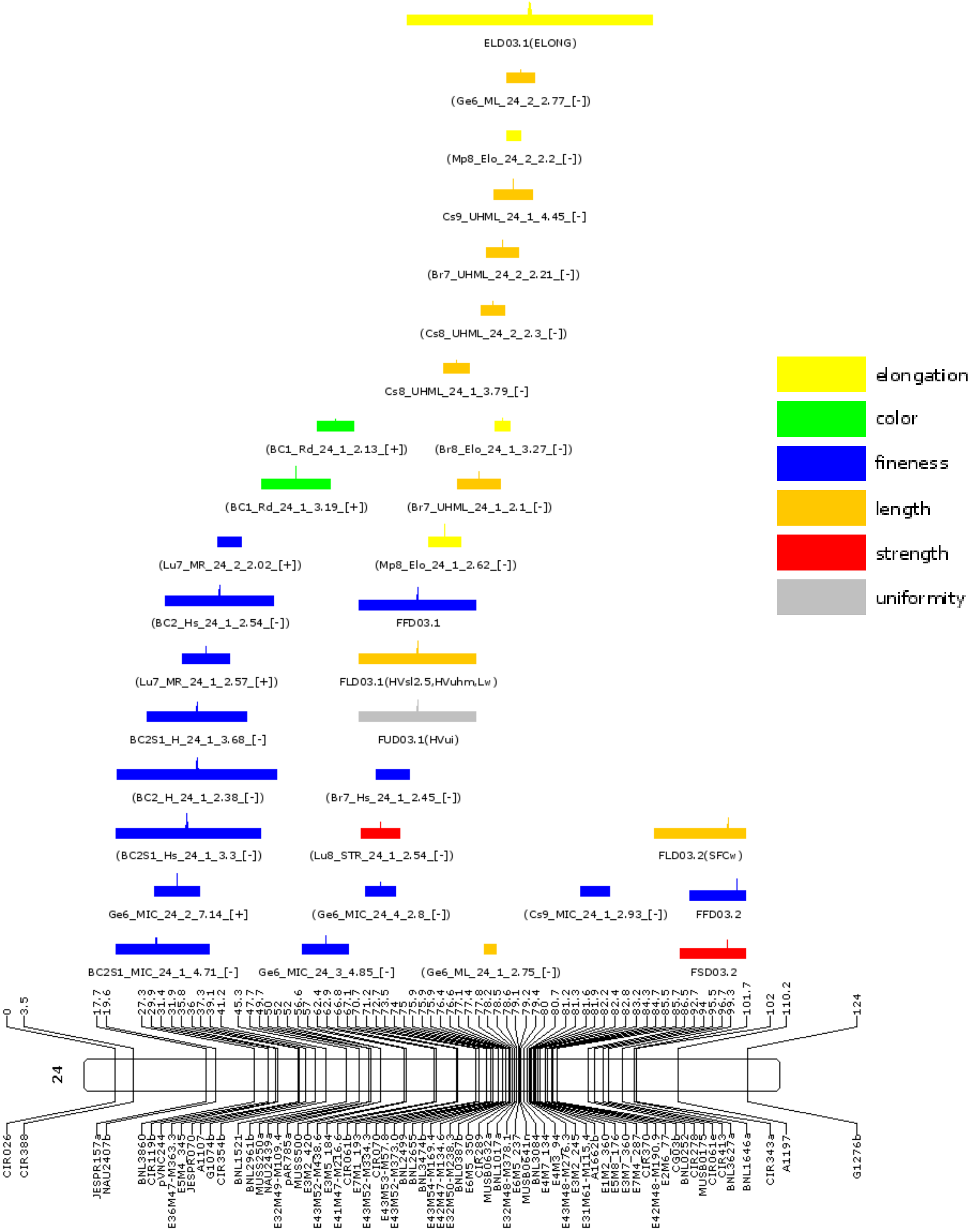

Chromosome 25

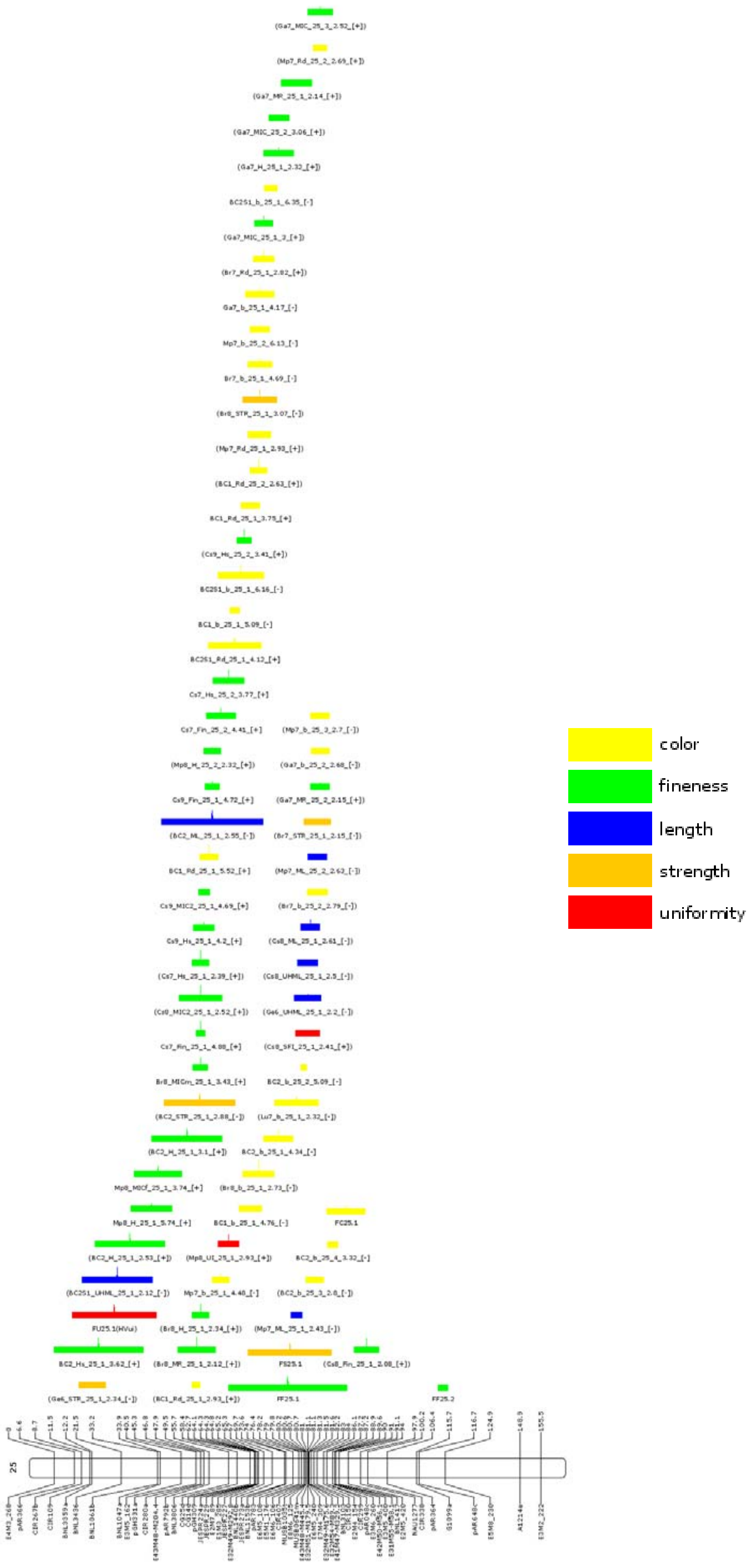

Chromosome 26

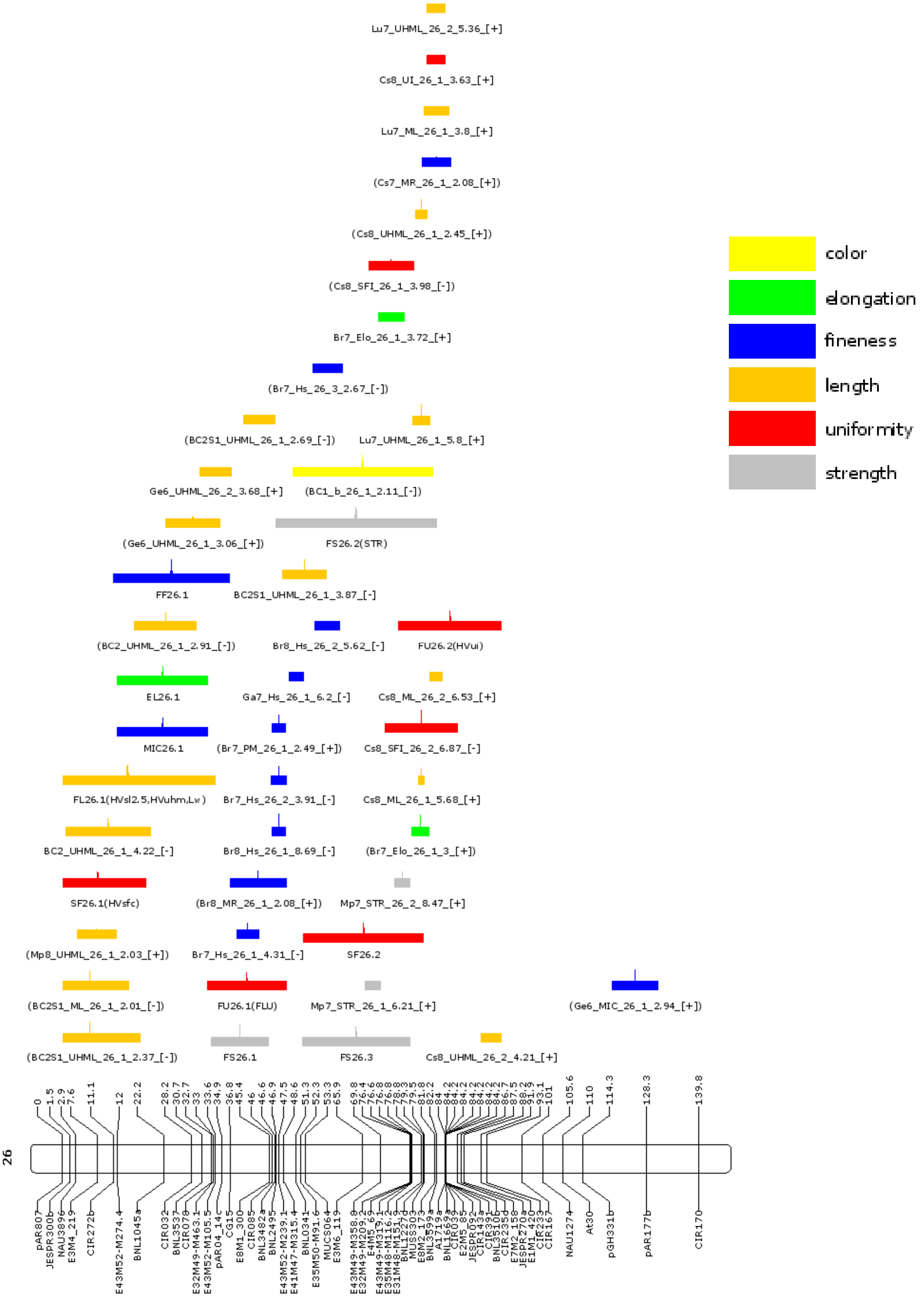

Supplement: Additional file 3 — Figure S1 (continuation of Figure 1): Same legend as Figure 1. [file 1471-2229-10-132-S3.PDF]
