# Supplementary material for: Meta-analysis of cotton fiber quality QTLs across diverse environments in a Gossypium hirsutum x G. barbadense RIL population
Source: BMC Plant Biol. 2010 Jun 28;10:132. doi: 10.1186/1471-2229-10-132 (PMC3017793; doi:10.1186/1471-2229-10-132)
Supplement: Additional file 4 — Table S3: Characteristics of the clusters detected by MetaQTL and list of meta-clusters. [file 1471-2229-10-132-S4.DOC]

**Additional file, Table S3: Characteristics of the clusters detected by MetaQTL and list of meta-clusters.** Position and 95%CI in cM on the consensus Guazuncho-2 x VH8-4602 map, and weight of respective cluster in the trait x chromosome combination (sum equal to 1), as determined by MetaQTL software. Flanking (left and right) markers are indicated as the closest SSR or RFLP locus (AFLP not considered) mapped upward and downward on the consensus BC-RIL map. Column “Solitary QTL” designates clusters of low reliability supported by a single data set. Meta-cluster designation in 1^st^ column (trait acronym and chromosome) is suffixed with a letter (A, B) when several meta-cluster are mapped on the same chromosome

| **Meta-cluster** | **Solitary**  **QTL** | **Cluster** | **Trait** | **Chrom.** | **cM** | **Weight** | **CI(95%)** | **Flanking left** | **Flanking right** |
| --- | --- | --- | --- | --- | --- | --- | --- | --- | --- |
| *ELO_2* |  | *QTLClust_ELO_2_1* | Elongation | 2 | 46.2 | 1.00 | 0.04 | BNL3971 | MUSB0749 |
| *FIN_2* |  | *QTLClust_FIN_2_1* | Fineness | 2 | 22.7 | 0.28 | 2.5 | Unig25A02 | BNL1897a |
| *FIN_2* |  | *QTLClust_FIN_2_2* | Fineness | 2 | 35.7 | 0.29 | 2.4 | JESPR179a | BNL3590 |
| *FIN_2* |  | *QTLClust_FIN_2_3* | Fineness | 2 | 43.2 | 0.38 | 1.8 | JESPR101b | BNL3971 |
|  | x | *QTLClust_FIN_2_4* | Fineness | 2 | 80.5 | 0.06 | 12.9 | NAU5384 | NAU3684 |
|  |  | *QTLClust_FIN_3_1* | Fineness | 3 | 0.1 | 0.13 | 3.0 | Terminal | BNL3408b |
|  |  | *QTLClust_FIN_3_2* | Fineness | 3 | 47.3 | 0.6 | 2.5 | NAU0884 | BNL0226b |
| *Indicative* |  | *QTLClust_FIN_3_3* | Fineness | 3 | 100.8 | 0.27 | 1.4 | JESPR231b | CIR228a |
|  |  | *QTLClust_LEN_3_1* | Length | 3 | 11.7 | 0.19 | 4.7 | BNL3408b | CIR030 |
| *LEN_3* |  | *QTLClust_LEN_3_2* | Length | 3 | 35.7 | 0.46 | 1.0 | BNL3441 | CIR058 |
| *LEN_3* |  | *QTLClust_LEN_3_3* | Length | 3 | 47.4 | 0.31 | 1.9 | NAU0884 | A1145 |
|  | x | *QTLClust_LEN_3_4* | Length | 3 | 80.7 | 0.04 | 1.7 | G1164a | CIR212a |
|  | x | *QTLClust_STR_3_1* | Strength | 3 | 33.9 | 0.11 | 26.7 | BNL2443b | BNL3441 |
|  | x | *QTLClust_STR_3_2* | Strength | 3 | 67.0 | 0.22 | 6.7 | BNL0226b | BNL3989 |
|  | x | *QTLClust_STR_3_3* | Strength | 3 | 82.7 | 0.11 | 3.5 | G1164a | CIR212a |
| *STR_3* |  | *QTLClust_STR_3_4* | Strength | 3 | 110.4 | 0.56 | 0.5 | CIR133 | NAU2161 |
| *LEN_4* |  | *QTLClust_LEN_4_1* | Length | 4 | 67.4 | 1.00 | 1.0 | CIR249 | NAU2231b |
| *Indicative* |  | *QTLClust_FIN_5_1* | Fineness | 5 | 25.4 | 0.36 | 6.2 | CIR062a | NAU0861 |
| *Indicative* |  | *QTLClust_FIN_5_2* | Fineness | 5 | 44.5 | 0.27 | 1.5 | CIR034 | BNL2448b |
| *Indicative* |  | *QTLClust_FIN_5_3* | Fineness | 5 | 49.8 | 0.18 | 3.2 | BNL3992 | BNL2656a |
|  |  | *QTLClust_FIN_5_4* | Fineness | 5 | 124.2 | 0.18 | 0.7 | CIR253 | NAU2121 |
| *Indicative* |  | *QTLClust_LEN_5_1* | Length | 5 | 22.1 | 0.09 | 5.0 | CIR224a | NAU1137 |
| *Indicative* |  | *QTLClust_LEN_5_2* | Length | 5 | 32.3 | 0.27 | 4.1 | A1135b | CIR280b |
| *Indicative* |  | *QTLClust_LEN_5_3* | Length | 5 | 48.7 | 0.18 | 3.6 | BNL3992 | BNL2656a |
|  |  | *QTLClust_LEN_5_4* | Length | 5 | 107.2 | 0.45 | 0.4 | A1691 | JESPR065b |
|  |  | *QTLClust_COL_6_1* | Color | 6 | 34.5 | 0.15 | 3.0 | A1215a | BNL1440a |
|  | x | *QTLClust_COL_6_2* | Color | 6 | 44.9 | 0.08 | 3.5 | A1215a | BNL1440a |
| *COL_6* |  | *QTLClust_COL_6_3* | Color | 6 | 50.7 | 0.77 | 0.1 | BNL1440a | BNL3594b |
|  |  | *QTLClust_COL_8_1* | Color | 8 | 28.2 | 0.08 | 4.8 | JESPR232a | CIR376a |
| *COL_8A* |  | *QTLClust_COL_8_2* | Color | 8 | 46.0 | 0.3 | 1.3 | JESPR066 | CIR237 |
| *COL_8A* |  | *QTLClust_COL_8_3* | Color | 8 | 51.0 | 0.04 | 2.5 | JESPR066 | CIR237 |
| *COL_8A* |  | *QTLClust_COL_8_4* | Color | 8 | 53.8 | 0.17 | 2.5 | CIR119a | CIR354a |
| *COL_8B* |  | *QTLClust_COL_8_5* | Color | 8 | 83.6 | 0.14 | 1.8 | BNL2961a | pAR785b |
| *COL_8B* |  | *QTLClust_COL_8_6* | Color | 8 | 88.0 | 0.04 | 3.4 | BNL2961a | pAR785b |
| *COL_8B* |  | *QTLClust_COL_8_7* | Color | 8 | 108.8 | 0.09 | 3.7 | NAU1164 | BNL3627c |
| *COL_8B* |  | *QTLClust_COL_8_8* | Color | 8 | 121.2 | 0.15 | 0.3 | BNL3792 | MUSB0818b |
| *FIN_9A* |  | *QTLClust_FIN_9_1* | Fineness | 9 | 19.9 | 0.16 | 5.3 | BNL0686a | pAR127b |
| *FIN_9A* |  | *QTLClust_FIN_9_2* | Fineness | 9 | 41.4 | 0.32 | 0.9 | MUSB1040b | NAU0966b |
| *FIN_9A* | x | *QTLClust_FIN_9_3* | Fineness | 9 | 50.5 | 0.04 | 7.0 | BNL3031b | JESPR230b |
|  | x | *QTLClust_FIN_9_4* | Fineness | 9 | 70.0 | 0.04 | 2.9 | BNL3582 | JESPR248b |
|  | x | *QTLClust_FIN_9_5* | Fineness | 9 | 81.2 | 0.04 | 2.0 | BNL1317b | NAU0858 |
| *FIN_9B* |  | *QTLClust_FIN_9_6* | Fineness | 9 | 94.7 | 0.16 | 1.2 | BNL2590 | MGHES46b |
| *FIN_9B* |  | *QTLClust_FIN_9_7* | Fineness | 9 | 101.5 | 0.24 | 0.3 | MGHES46b | MUSS022 |
| *LEN_9* | x | *QTLClust_LEN_9_1* | Length | 9 | 18.0 | 0.13 | 9.9 | pAR144g | BNL0686a |
| *LEN_9* |  | *QTLClust_LEN_9_2* | Length | 9 | 35.7 | 0.74 | 2.5 | NAU3159a | CIR227 |
| *LEN_9* |  | *QTLClust_LEN_9_3* | Length | 9 | 39.3 | 0.13 | 0.3 | MUSB1040b | NAU0966b |
|  |  | *QTLClust_FIN_10_1* | Fineness | 10 | 4.9 | 0.18 | 7.0 | A1158a | A1163a |
|  |  | *QTLClust_FIN_10_2* | Fineness | 10 | 16.3 | 0.2 | 4.6 | CIR372 | BNL0256 |
| *FIN_10* |  | *QTLClust_FIN_10_3* | Fineness | 10 | 57.5 | 0.13 | 4.1 | CIR291e | CIR171a |
| *FIN_10* |  | *QTLClust_FIN_10_4* | Fineness | 10 | 69.8 | 0.25 | 3.6 | CIR171a | BNL3895 |
| *FIN_10* |  | *QTLClust_FIN_10_5* | Fineness | 10 | 80.9 | 0.18 | 7.7 | pAR02_18 | BNL1161b |
|  |  | *QTLClust_FIN_10_6* | Fineness | 10 | 114.0 | 0.06 | 23.3 | BNL3563 | BNL2960 |
| *FIN_12* |  | *QTLClust_FIN_12_1* | Fineness | 12 | 3.5 | 0.22 | 1.4 | JESPR300a | CIR272a |
| *FIN_12* |  | *QTLClust_FIN_12_2* | Fineness | 12 | 14.0 | 0.33 | 4.3 | BNL4059 | CIR362 |
|  |  | *QTLClust_FIN_12_3* | Fineness | 12 | 40.3 | 0.1 | 3.5 | NAU0915 | CIR081 |
|  |  | *QTLClust_FIN_12_4* | Fineness | 12 | 56.2 | 0.15 | 2.9 | BNL1679 | BNL1673 |
|  |  | *QTLClust_FIN_12_5* | Fineness | 12 | 71.3 | 0.05 | 4.2 | BNL1679 | BNL1673 |
|  |  | *QTLClust_FIN_12_6* | Fineness | 12 | 82.1 | 0.15 | 0.3 | BNL1707a | BNL3261b |
|  | x | *QTLClust_ELO_15_1* | Elongation | 15 | 33.7 | 0.09 | 18.7 | JESPR152 | pAR019b |
| *ELO_15* |  | *QTLClust_ELO_15_2* | Elongation | 15 | 66.7 | 0.55 | 2.2 | MUSB0818c | JESPR102a |
| *ELO_15* |  | *QTLClust_ELO_15_3* | Elongation | 15 | 72.3 | 0.27 | 4.0 | BNL1350b | A1553 |
|  | x | *QTLClust_ELO_15_4* | Elongation | 15 | 85.8 | 0.09 | 3.3 | BNL3090b | BNL0786 |
|  |  | *QTLClust_FIN_15_1* | Fineness | 15 | 48.3 | 0.07 | 7.7 | pAR019b | BNL4082 |
| *FIN_15* |  | *QTLClust_FIN_15_2* | Fineness | 15 | 61.7 | 0.19 | 2.0 | CIR143b | MUSB0818c |
| *FIN_15* |  | *QTLClust_FIN_15_3* | Fineness | 15 | 67.3 | 0.14 | 0.9 | JESPR102a | MUSS422a |
| *FIN_15* |  | *QTLClust_FIN_15_4* | Fineness | 15 | 73.4 | 0.15 | 2.8 | BNL1350b | A1553 |
| *FIN_15* |  | *QTLClust_FIN_15_5* | Fineness | 15 | 83.7 | 0.34 | 3.0 | BNL3090b | BNL0786 |
|  |  | *QTLClust_FIN_15_6* | Fineness | 15 | 108.8 | 0.11 | 1.6 | NAU2419c | BNL3345 |
| *Indicative* |  | *QTLClust_FIN_16_1* | Fineness | 16 | 41.0 | 0.1 | 5.6 | BNL1604a | BNL1122b |
| *Indicative* |  | *QTLClust_FIN_16_2* | Fineness | 16 | 49.5 | 0.4 | 2.0 | BNL1604a | BNL1122b |
| *Indicative* |  | *QTLClust_FIN_16_3* | Fineness | 16 | 63.7 | 0.1 | 6.3 | JESPR228a | NAU2432a |
|  |  | *QTLClust_FIN_16_4* | Fineness | 16 | 82.7 | 0.1 | 6.1 | NAU2186b | NAU0751 |
|  |  | *QTLClust_FIN_16_5* | Fineness | 16 | 89.6 | 0.1 | 5.8 | MGHES75 | JESPR292 |
|  |  | *QTLClust_FIN_16_6* | Fineness | 16 | 115.7 | 0.2 | 1.2 | G1158a | NAU1020b |
| *FIN_17* |  | *QTLClust_FIN_17_1* | Fineness | 17 | 18.4 | 0.49 | 3.2 | BNL2443a | JESPR195 |
| *FIN_17* |  | *QTLClust_FIN_17_2* | Fineness | 17 | 27.8 | 0.51 | 0.4 | G1258b | JESPR101a |
|  |  | *QTLClust_FIN_18_1* | Fineness | 18 | 10.0 | 0.06 | 9.9 | BNL3281a | CIR216 |
|  |  | *QTLClust_FIN_18_2* | Fineness | 18 | 27.5 | 0.18 | 4.1 | JESPR204b | NAU3130 |
| *FIN_18* |  | *QTLClust_FIN_18_3* | Fineness | 18 | 49.0 | 0.47 | 1.4 | CIR277a | MUCS405 |
|  |  | *QTLClust_FIN_18_4* | Fineness | 18 | 70.9 | 0.06 | 10.0 | MUSS203c | CIR235a |
|  |  | *QTLClust_FIN_18_5* | Fineness | 18 | 81.7 | 0.24 | 0.1 | BNL1721 | BNL1079 |
| *Indicative* |  | *QTLClust_ELO_19_1* | Elongation | 19 | 83.2 | 0.22 | 4.6 | JESPR230c | BNL3811a |
| *Indicative* |  | *QTLClust_ELO_19_2* | Elongation | 19 | 91.5 | 0.09 | 4.3 | NAU5273 | CIR062b |
| *Indicative* |  | *QTLClust_ELO_19_3* | Elongation | 19 | 102.0 | 0.22 | 3.0 | CIR240 | BNL2448a |
| *Indicative* |  | *QTLClust_ELO_19_4* | Elongation | 19 | 135.4 | 0.26 | 3.6 | CIR168 | CG25a |
| *Indicative* |  | *QTLClust_ELO_19_5* | Elongation | 19 | 148.3 | 0.21 | 1.9 | CIR179b | BNL1671 |
|  |  | *QTLClust_FIN_19_1* | Fineness | 19 | 0.6 | 0.11 | 9.5 | CIR415a | pGH474 |
| *Indicative* |  | *QTLClust_FIN_19_2* | Fineness | 19 | 51.4 | 0.22 | 5.8 | pAR825 | CIR176 |
| *Indicative* |  | *QTLClust_FIN_19_3* | Fineness | 19 | 74.2 | 0.11 | 3.2 | CIR024 | pAR988b |
| *Indicative* |  | *QTLClust_FIN_19_4* | Fineness | 19 | 83.4 | 0.06 | 4.8 | JESPR230c | BNL3811a |
| *Indicative* |  | *QTLClust_FIN_19_5* | Fineness | 19 | 102.1 | 0.28 | 2.8 | BNL2448a | NAU0911 |
| *Indicative* |  | *QTLClust_FIN_19_6* | Fineness | 19 | 189.0 | 0.17 | 4.8 | NAU2231a | CIR344 |
| *Indicative* |  | *QTLClust_FIN_19_7* | Fineness | 19 | 199.8 | 0.06 | 0.1 | CIR344 | CMS21a |
|  |  | *QTLClust_LEN_19_1* | Length | 19 | 25.7 | 0.07 | 12.5 | BNL3452 | pAR482 |
|  |  | *QTLClust_LEN_19_2* | Length | 19 | 63.5 | 0.21 | 4.2 | BNL0285 | BNL1611 |
|  |  | *QTLClust_LEN_19_3* | Length | 19 | 83.4 | 0.13 | 2.2 | JESPR230c | BNL3811a |
|  |  | *QTLClust_LEN_19_4* | Length | 19 | 101.3 | 0.33 | 1.6 | BNL3811b | CIR240 |
|  |  | *QTLClust_LEN_19_5* | Length | 19 | 149.2 | 0.27 | 0.4 | MUSB0641a | JESPR273b |
|  | x | *QTLClust_FIN_21_1* | Fineness | 21 | 49.2 | 0.04 | 3.7 | BNL0836b | BNL3649a |
| *FIN_21A* |  | *QTLClust_FIN_21_2* | Fineness | 21 | 65.1 | 0.08 | 3.7 | BNL3649a | CIR398 |
| *FIN_21A* |  | *QTLClust_FIN_21_3* | Fineness | 21 | 73.2 | 0.09 | 3.3 | CIR408b | CIR068 |
| *FIN_21A* |  | *QTLClust_FIN_21_4* | Fineness | 21 | 80.7 | 0.09 | 2.4 | CIR410 | pAR038 |
| *FIN_21A* |  | *QTLClust_FIN_21_5* | Fineness | 21 | 92.8 | 0.22 | 3.6 | NAU2950 | NAU4855 |
| *FIN_21B* |  | *QTLClust_FIN_21_6* | Fineness | 21 | 129.0 | 0.21 | 4.7 | BNL3147b | pAR570b |
| *FIN_21B* |  | *QTLClust_FIN_21_7* | Fineness | 21 | 149.0 | 0.14 | 3.5 | pAR570b | CIR013 |
| *FIN_21B* |  | *QTLClust_FIN_21_8* | Fineness | 21 | 162.5 | 0.09 | 1.9 | CIR156 | CIR254b |
|  | x | *QTLClust_STR_21_1* | Strength | 21 | 48.3 | 0.13 | 13.4 | BNL0836b | BNL1551a |
| *STR_21* |  | *QTLClust_STR_21_2* | Strength | 21 | 75.0 | 0.61 | 3.1 | CIR061a | BNL2805b |
| *STR_21* |  | *QTLClust_STR_21_3* | Strength | 21 | 80.3 | 0.14 | 5.4 | CIR410 | pAR451b |
|  | x | *QTLClust_STR_21_4* | Strength | 21 | 178.9 | 0.12 | 14.7 | CIR254b | BNL1705 |
|  | x | *QTLClust_LEN_23_1* | Length | 23 | 5.0 | 0.1 | 10.6 | CIR198 | BNL0686b |
| *LEN_23* |  | *QTLClust_LEN_23_2* | Length | 23 | 98.0 | 0.6 | 5.2 | JESPR110 | BNL1030a |
| *LEN_23* |  | *QTLClust_LEN_23_3* | Length | 23 | 117.3 | 0.3 | 1.0 | BNL3173b | Terminal |
|  | x | *QTLClust_STR_23_1* | Strength | 23 | 28.2 | 0.11 | 15.8 | BNL3383 | pAR547 |
|  | x | *QTLClust_STR_23_2* | Strength | 23 | 46.6 | 0.11 | 9.2 | MUSB1040a | NAU5465 |
| *Indicative* |  | *QTLClust_STR_23_3* | Strength | 23 | 85.9 | 0.31 | 1.9 | CIR200 | A1471a |
| *Indicative* |  | *QTLClust_STR_23_4* | Strength | 23 | 102.2 | 0.47 | 1.3 | JESPR114 | CIR194 |
| *LEN_24* |  | *QTLClust_LEN_24_1* | Length | 24 | 67.0 | 0.15 | 5.6 | MUSS500 | CIR061b |
| *LEN_24* |  | *QTLClust_LEN_24_2* | Length | 24 | 74.6 | 0.85 | 0.4 | CIR070 | BNL2499 |
| *COL_25A* |  | *QTLClust_COL_25_1* | Color | 25 | 46.0 | 0.08 | 2.5 | pGH331a | CIR280a |
| *COL_25A* |  | *QTLClust_COL_25_2* | Color | 25 | 56.7 | 0.12 | 2.7 | BNL3806 | JESPR224a |
| *COL_25A* |  | *QTLClust_COL_25_3* | Color | 25 | 66.1 | 0.48 | 2.0 | JESPR227 | BNL1440b |
| *COL_25B* |  | *QTLClust_COL_25_4* | Color | 25 | 79.8 | 0.07 | 2.0 | BNL1153b | CIR407 |
| *COL_25B* |  | *QTLClust_COL_25_5* | Color | 25 | 84.4 | 0.21 | 2.5 | CIR150 | CIR299 |
|  |  | *QTLClust_COL_25_6* | Color | 25 | 88.9 | 0.04 | 0.8 | CIR299 | BNL1417 |
|  |  | *QTLClust_FIN_25_1* | Fineness | 25 | 29.3 | 0.16 | 8.7 | BNL3436 | BNL1061b |
| *FIN_25* |  | *QTLClust_FIN_25_2* | Fineness | 25 | 47.9 | 0.49 | 1.6 | CIR280a | pAR792b |
|  |  | *QTLClust_FIN_25_3* | Fineness | 25 | 59.9 | 0.07 | 4.5 | BNL3806 | JESPR224a |
|  |  | *QTLClust_FIN_25_4* | Fineness | 25 | 70.7 | 0.16 | 3.8 | BNL1440b | JESPR273a |
|  |  | *QTLClust_FIN_25_5* | Fineness | 25 | 88.9 | 0.12 | 1.2 | CIR299 | BNL1417 |
| *Indicative* |  | *QTLClust_LEN_26_1* | Length | 26 | 7.5 | 0.25 | 6.9 | NAU3896 | CIR272b |
| *Indicative* |  | *QTLClust_LEN_26_2* | Length | 26 | 30.7 | 0.19 | 5.6 | CIR032 | CIR078 |
| *Indicative* |  | *QTLClust_LEN_26_3* | Length | 26 | 46.6 | 0.13 | 5.7 | CIR085 | BNL2495 |
| *Indicative* |  | *QTLClust_LEN_26_4* | Length | 26 | 79.6 | 0.37 | 1.1 | BNL1227d | BNL3599a |
| *Indicative* | x | *QTLClust_LEN_26_5* | Length | 26 | 94.2 | 0.06 | 1.3 | CIR233 | CIR167 |
