# Supplementary figures and images for: Meta-analysis of cotton fiber quality QTLs across diverse environments in a Gossypium hirsutum x G. barbadense RIL population
Source: BMC Plant Biol. 2010 Jun 28;10:132. doi: 10.1186/1471-2229-10-132 (PMC3017793; doi:10.1186/1471-2229-10-132)

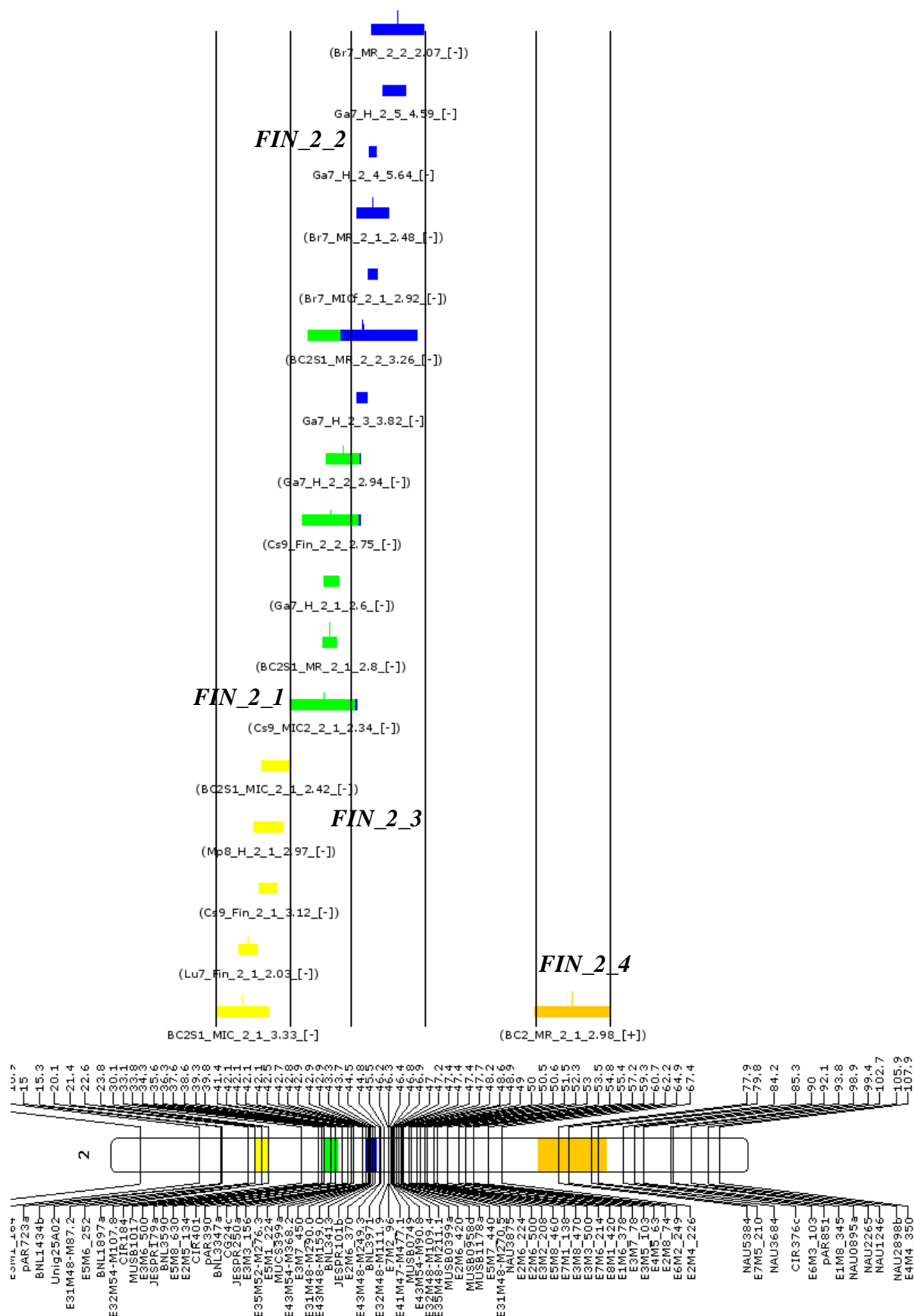



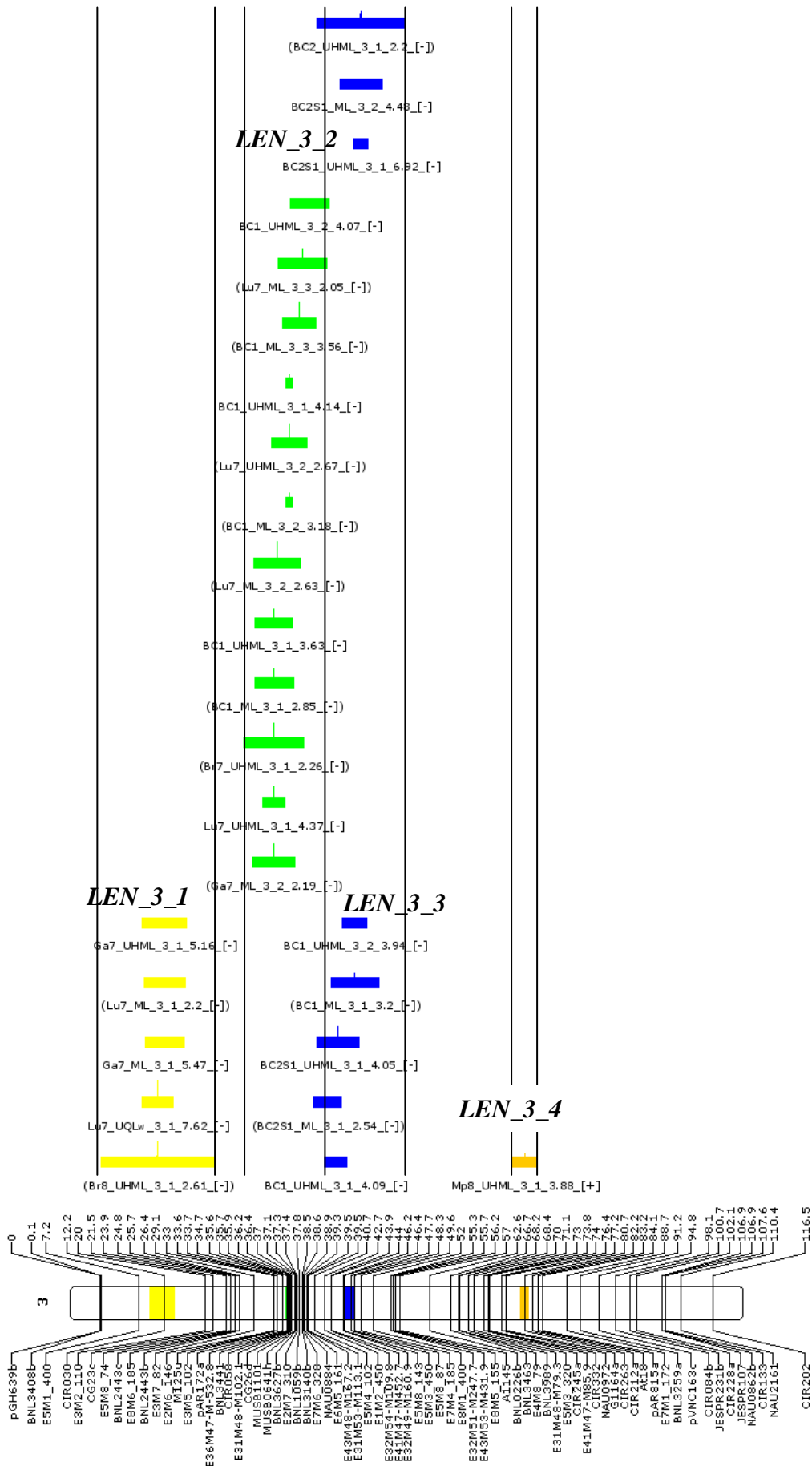

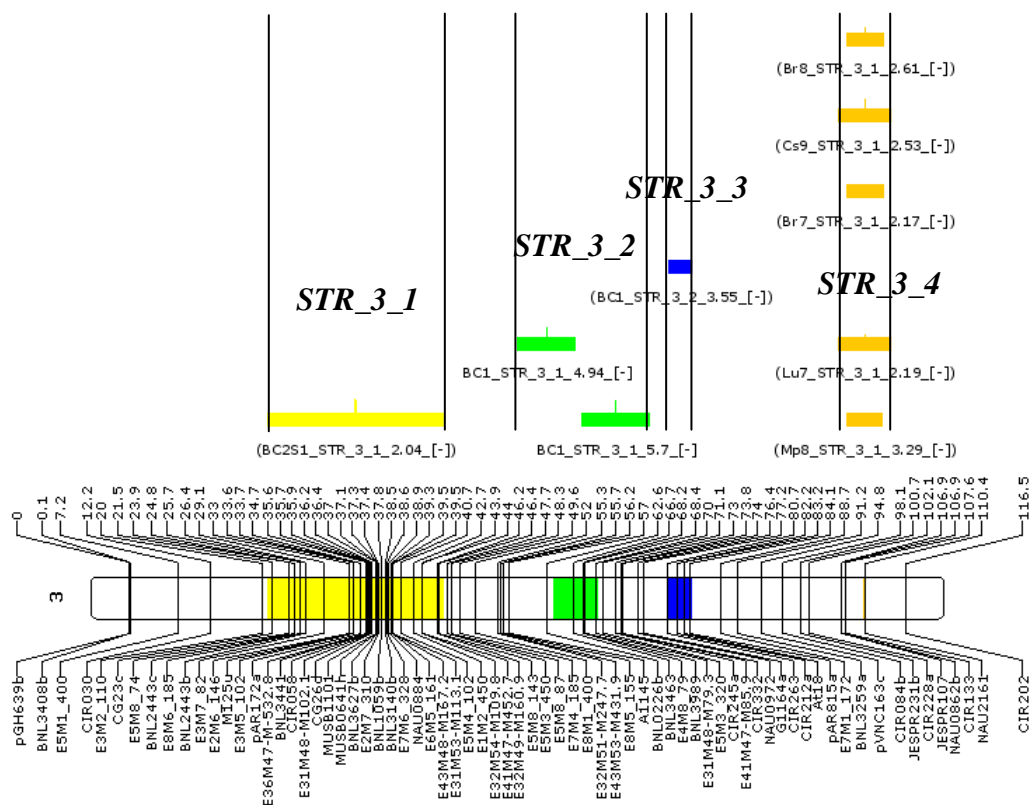

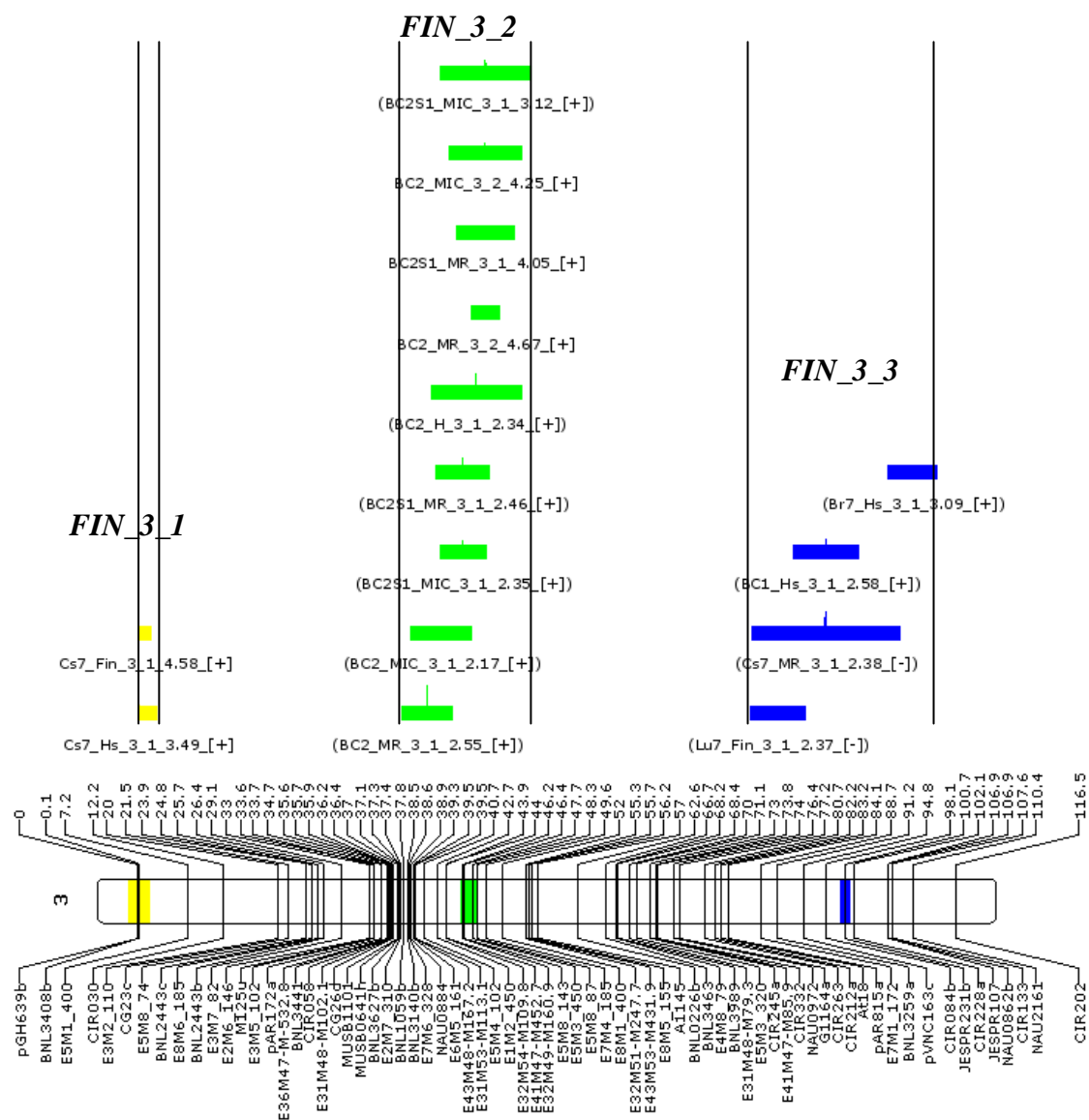

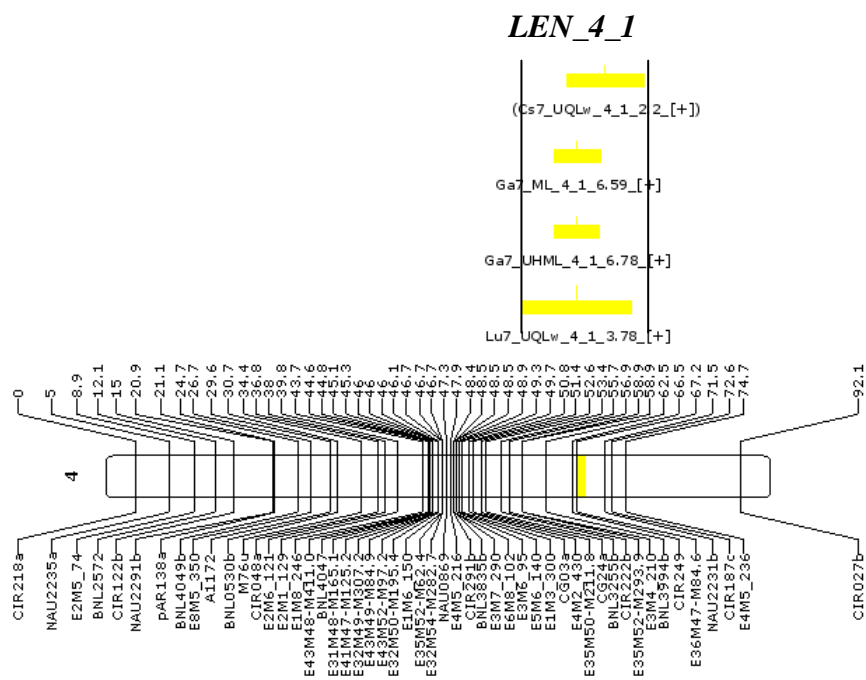

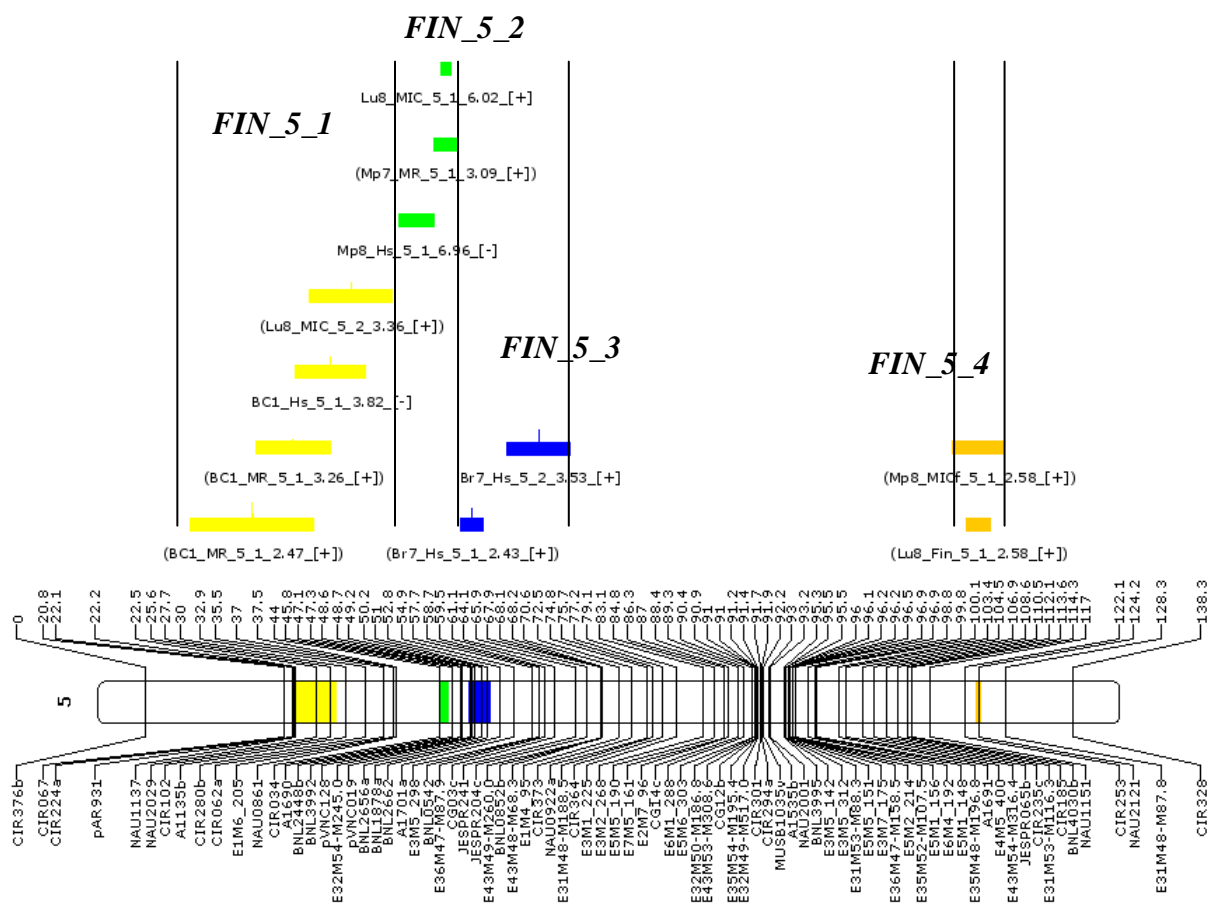

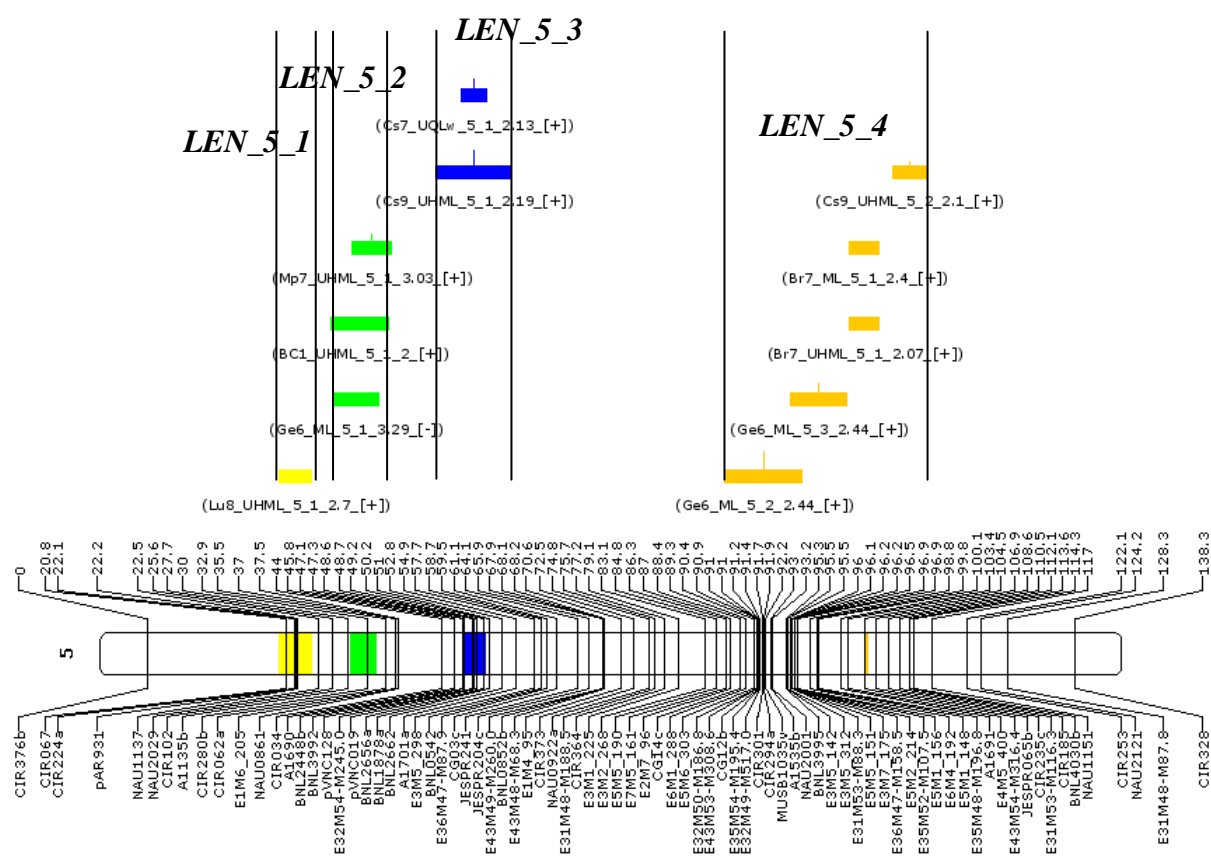

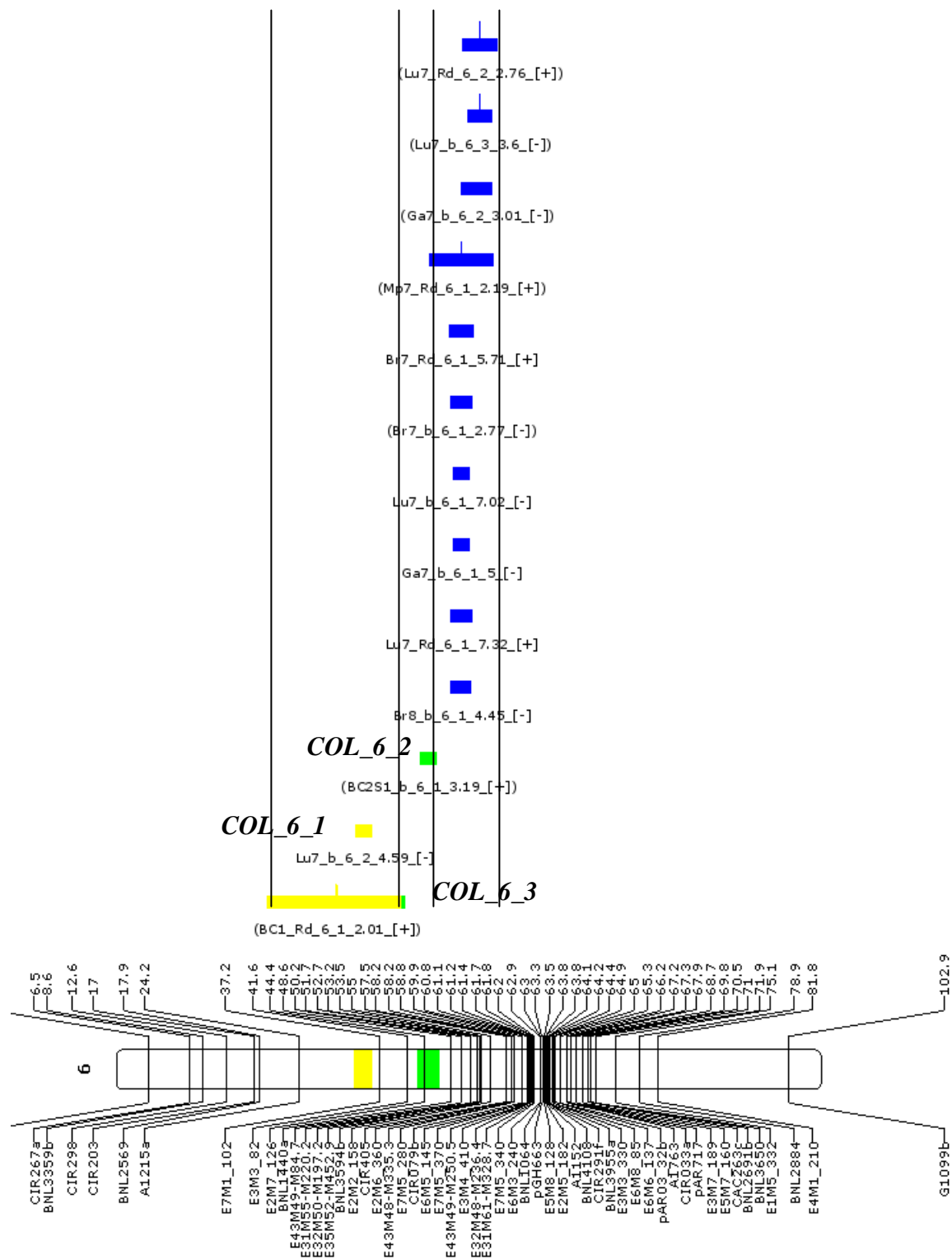

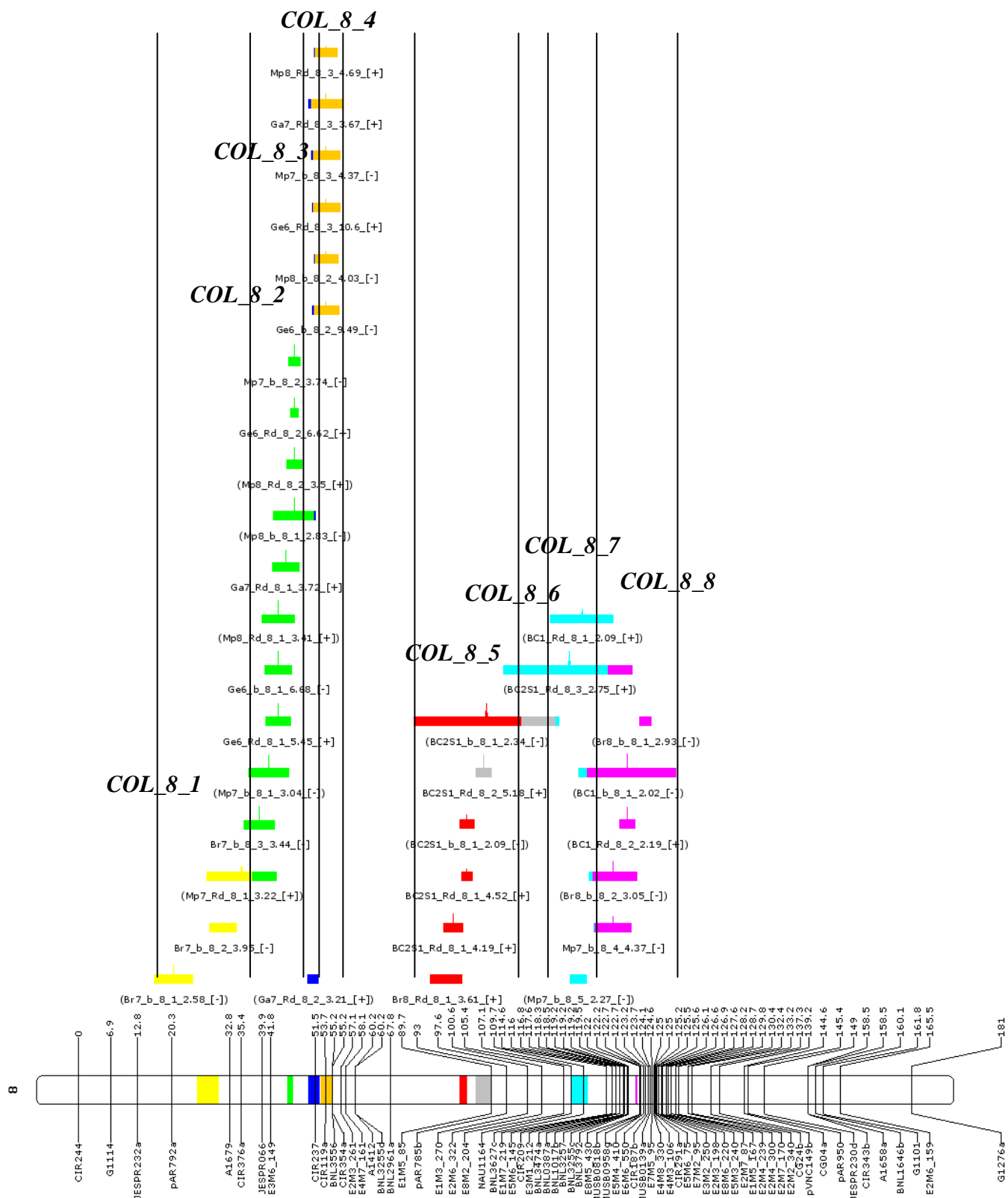

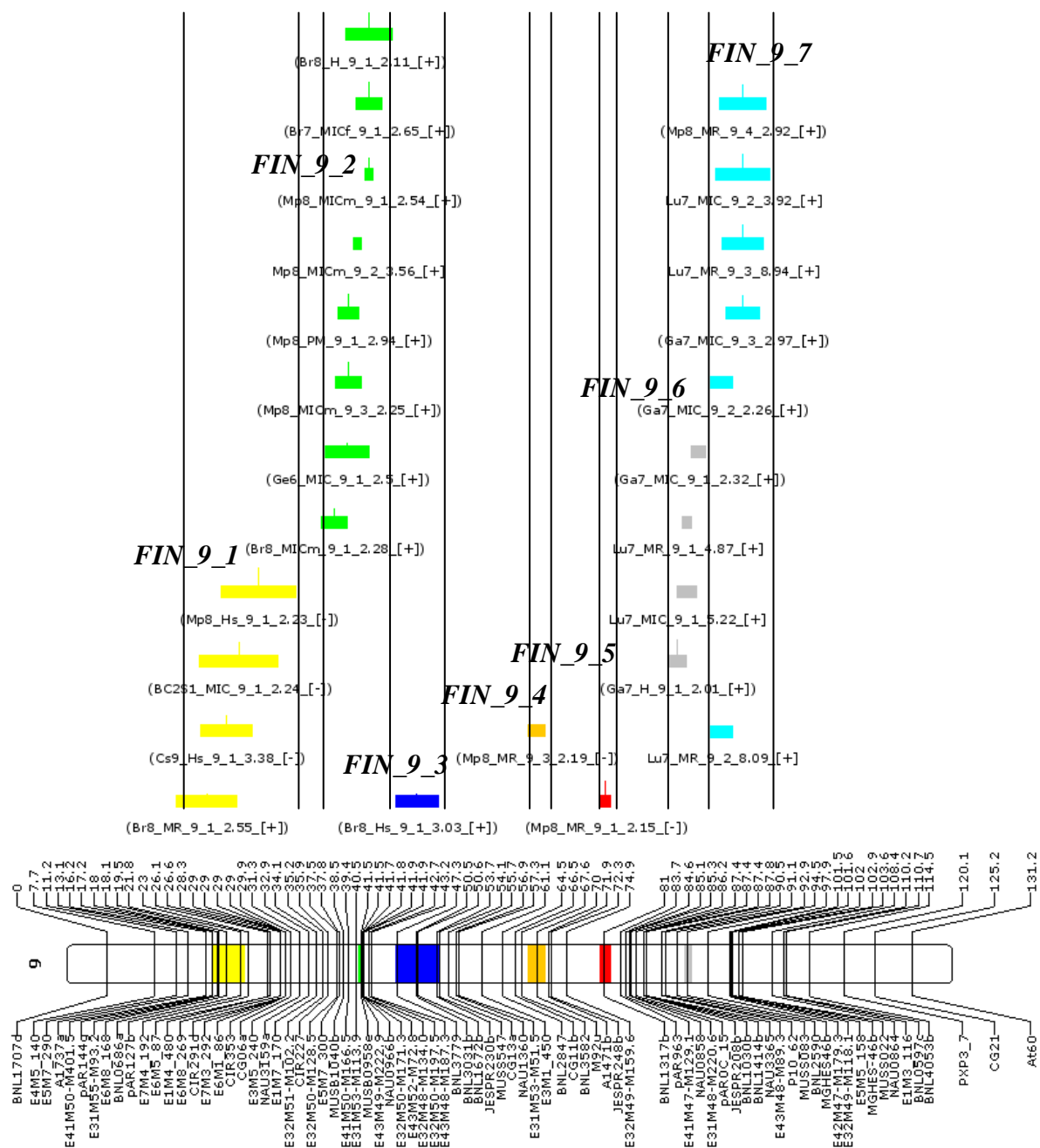

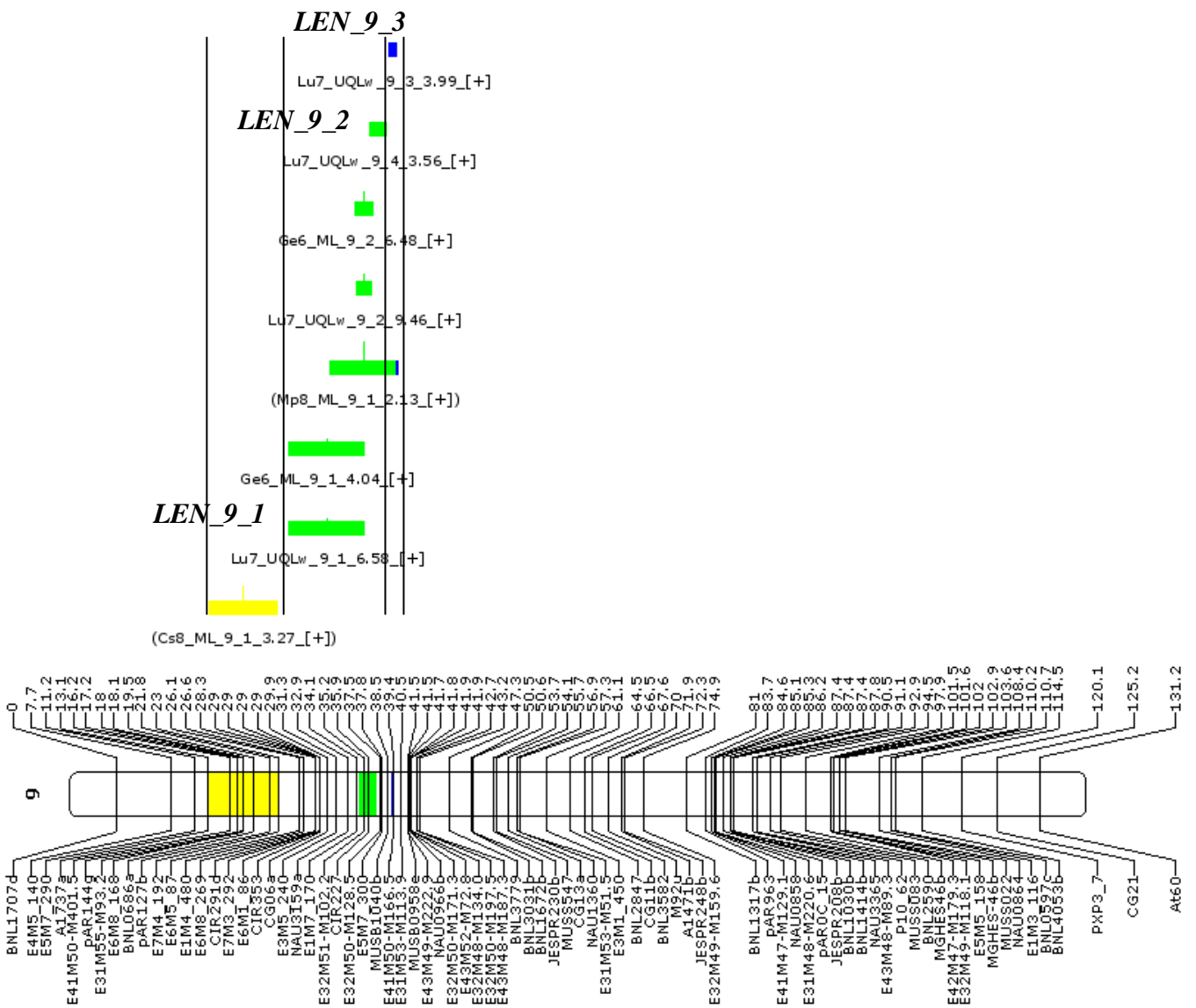

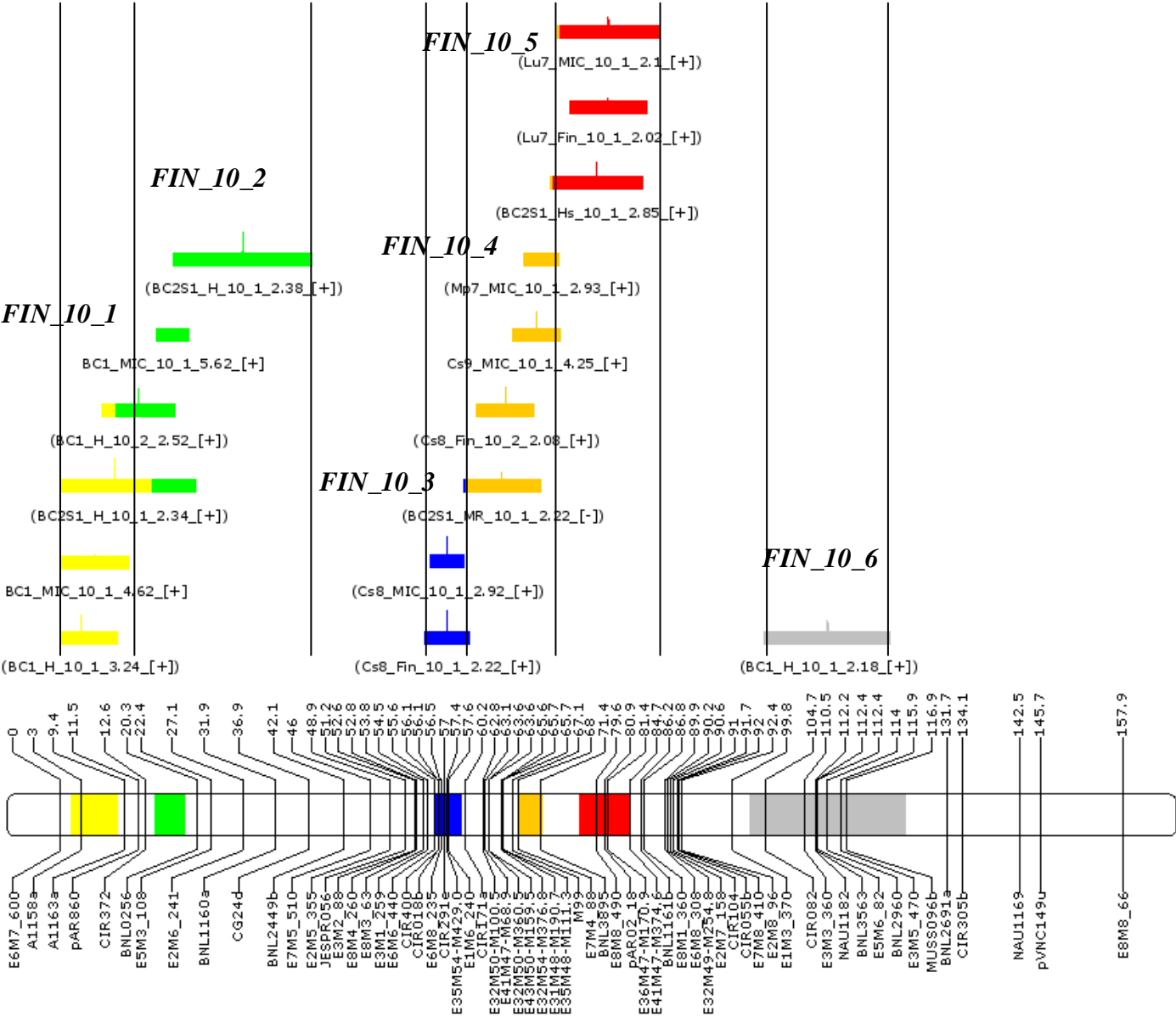

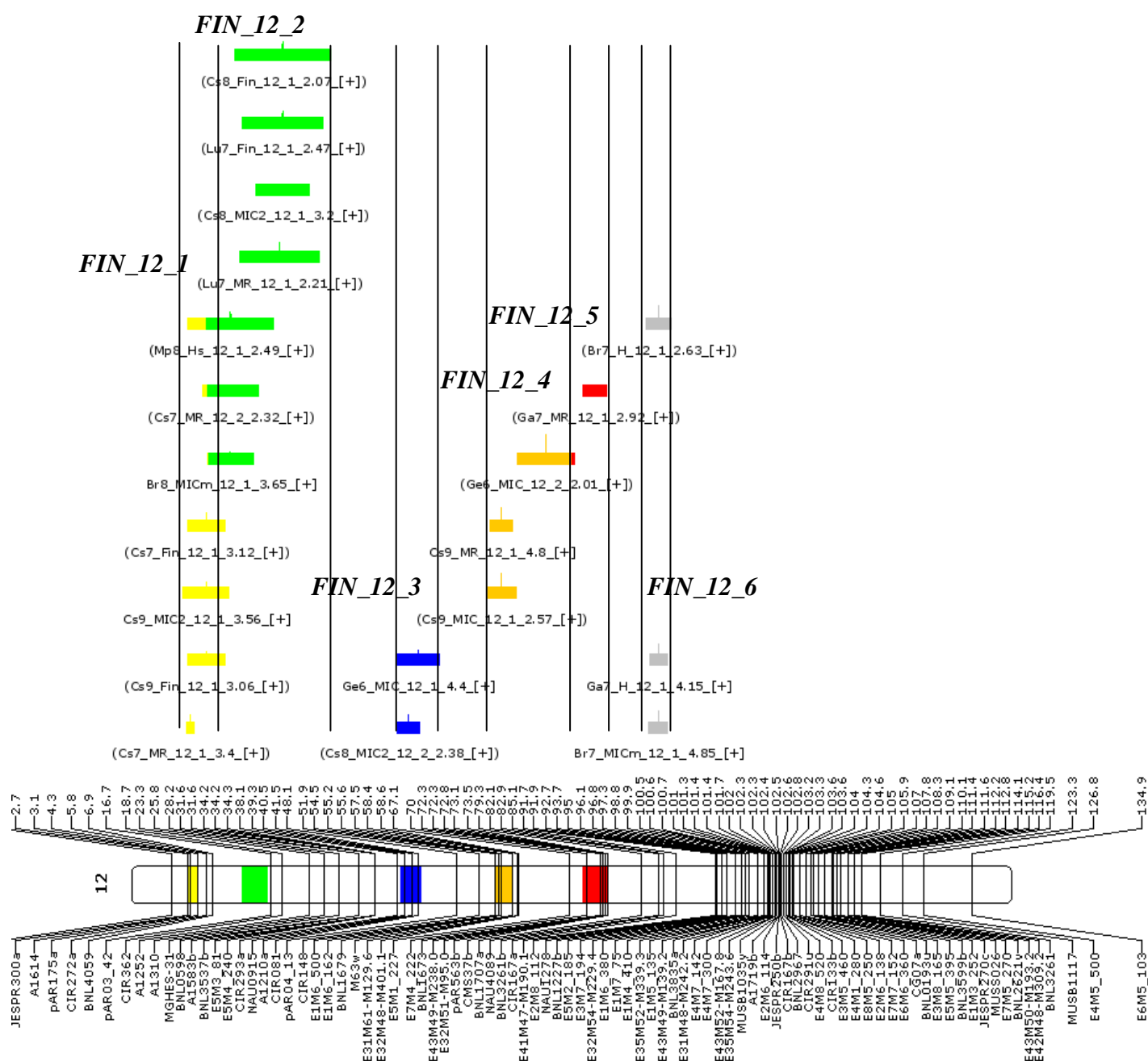

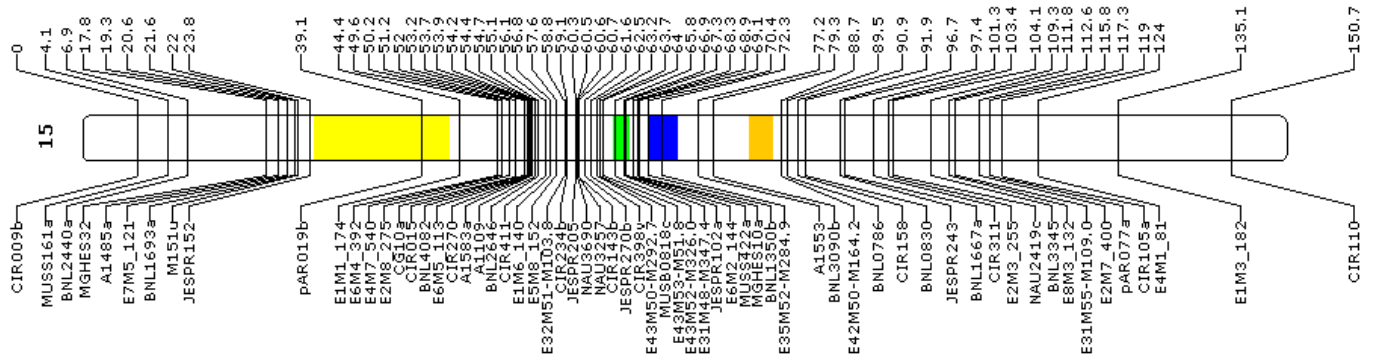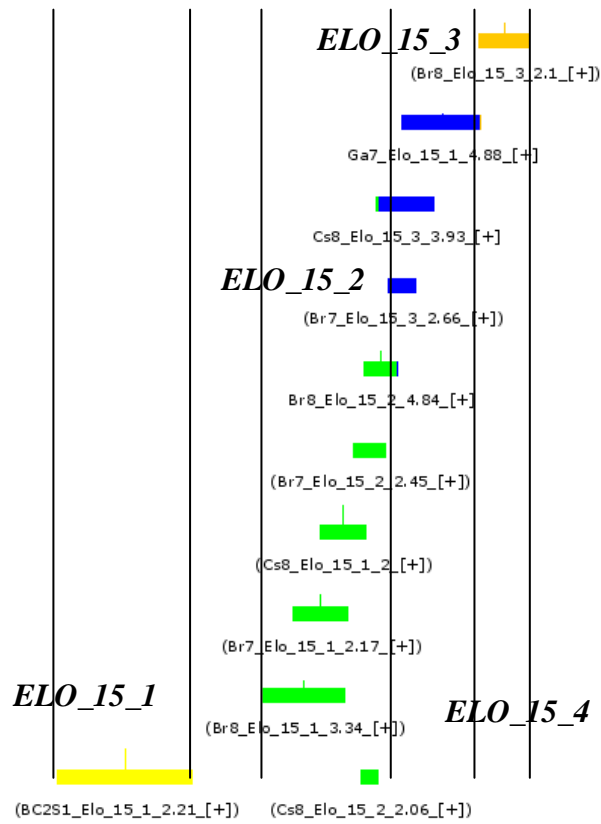

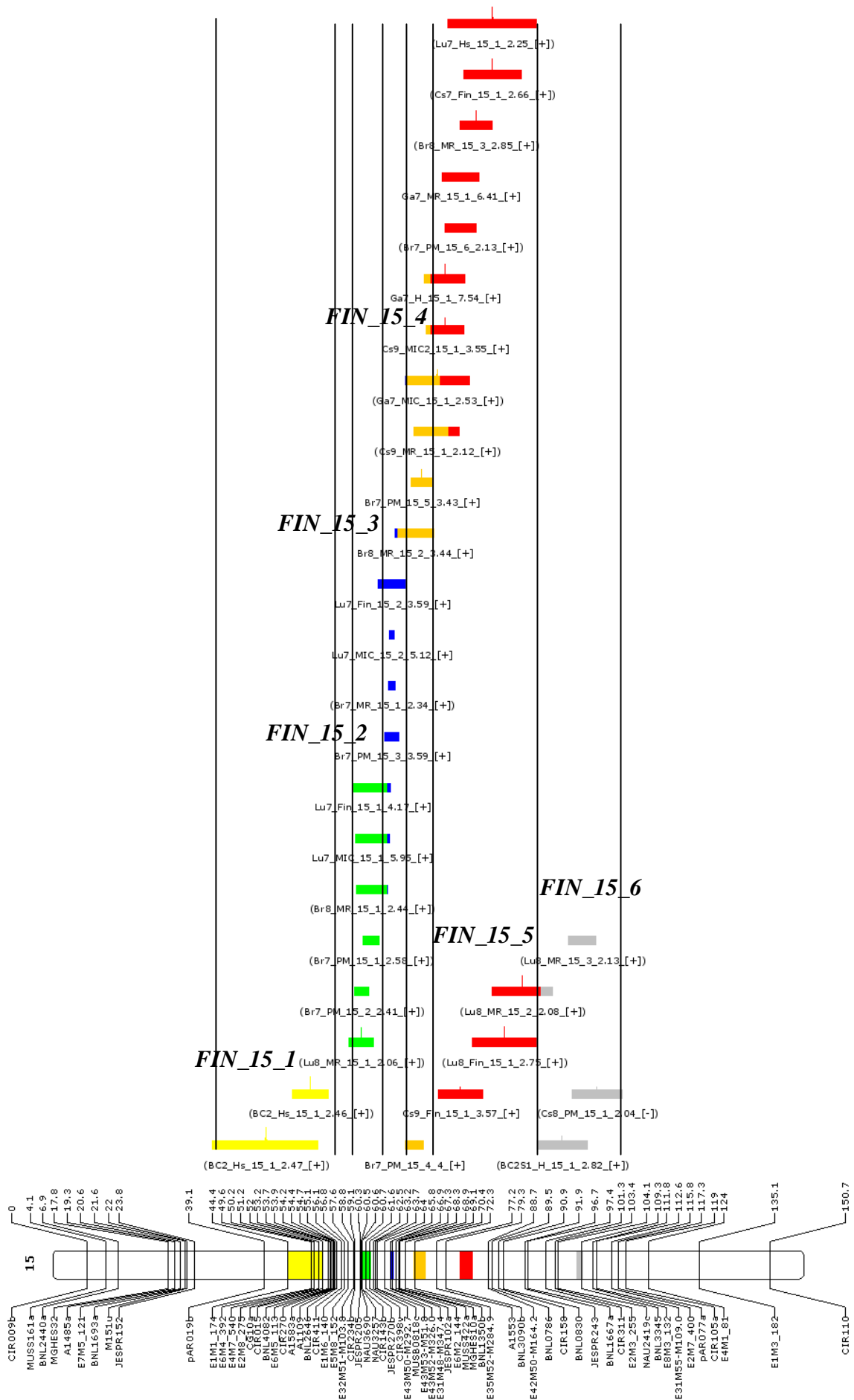

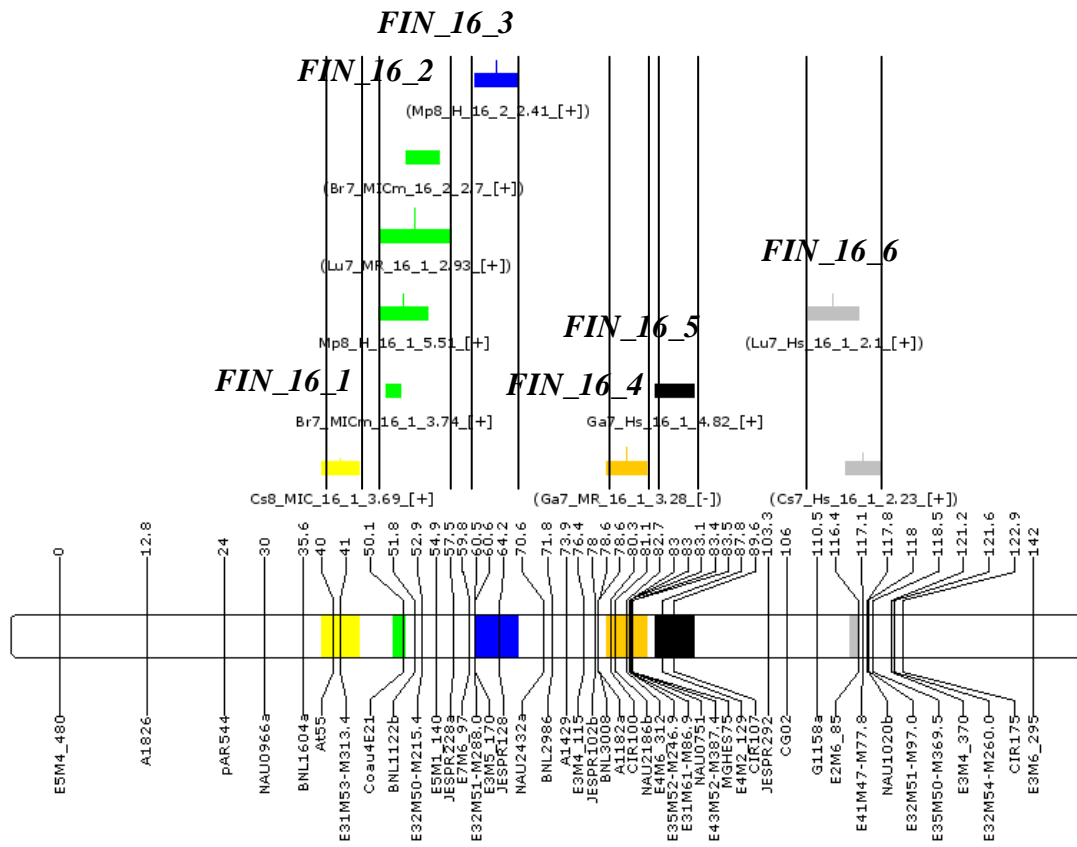

*FIN\_17\_2*

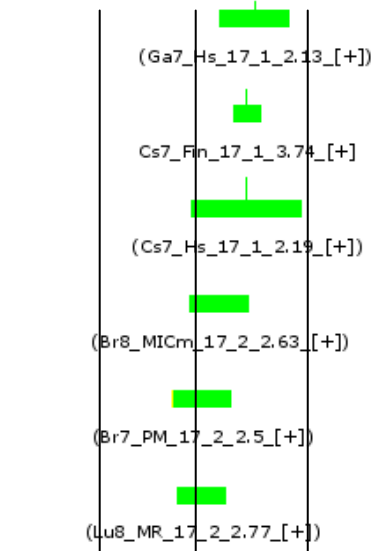

*FIN\_17\_1*

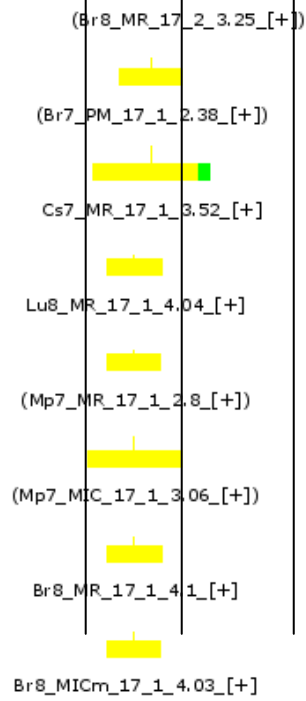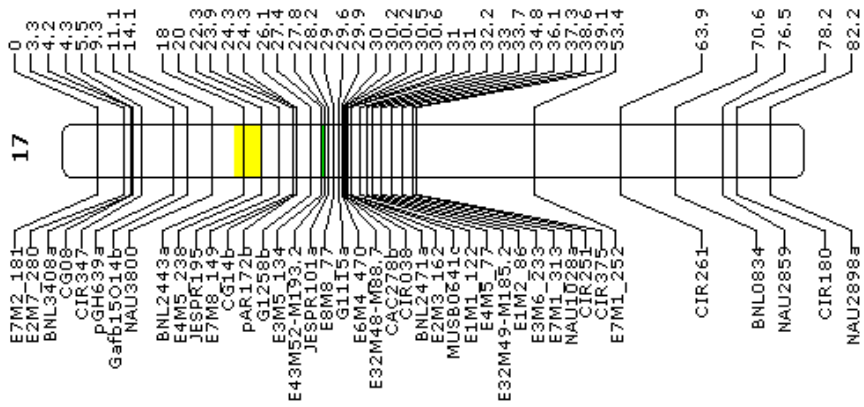

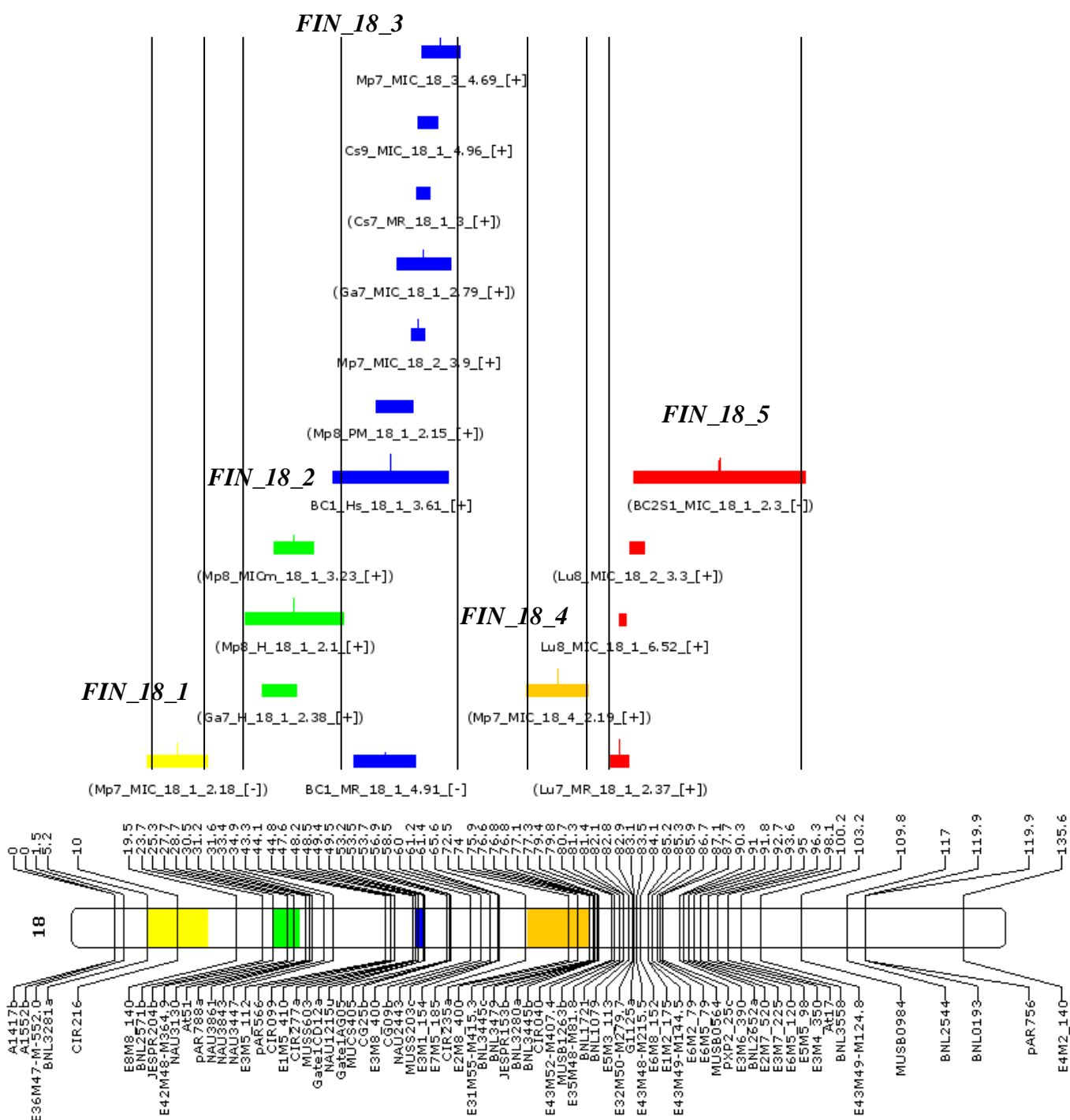

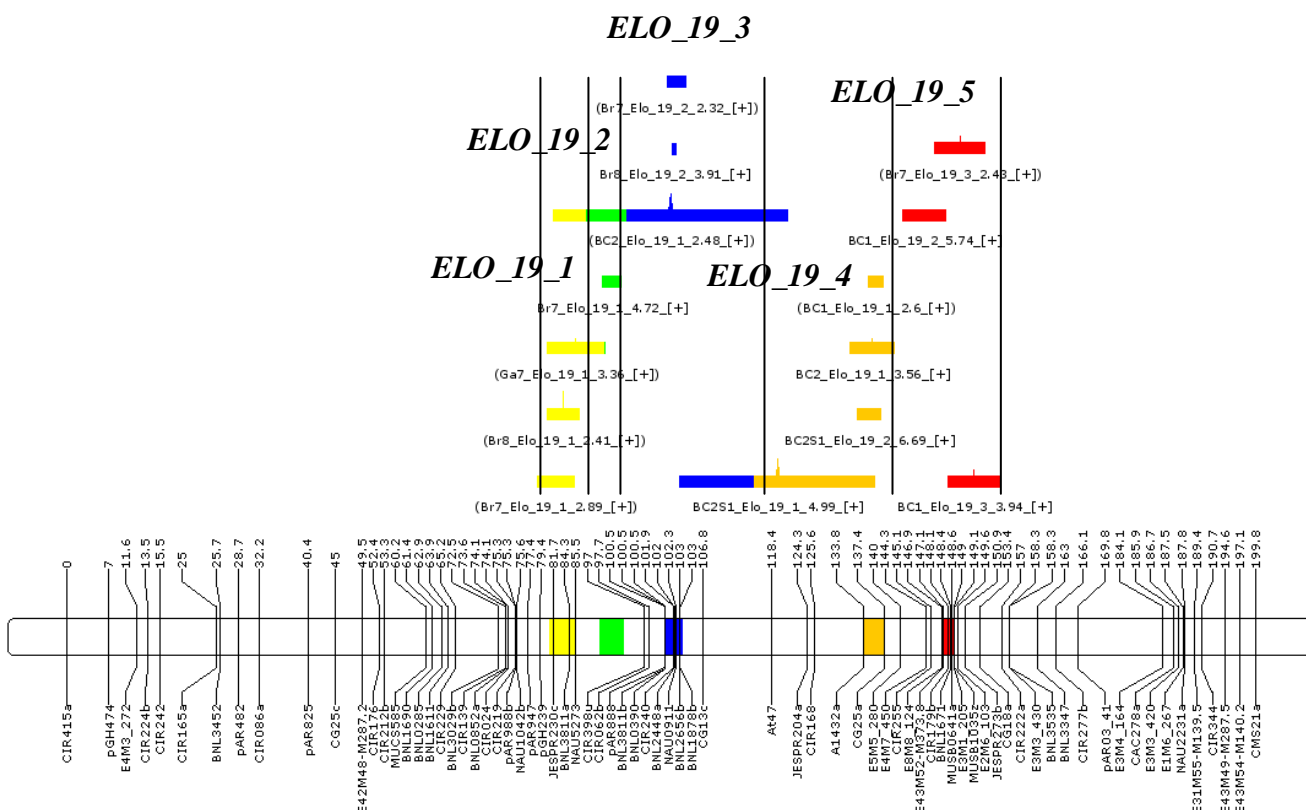

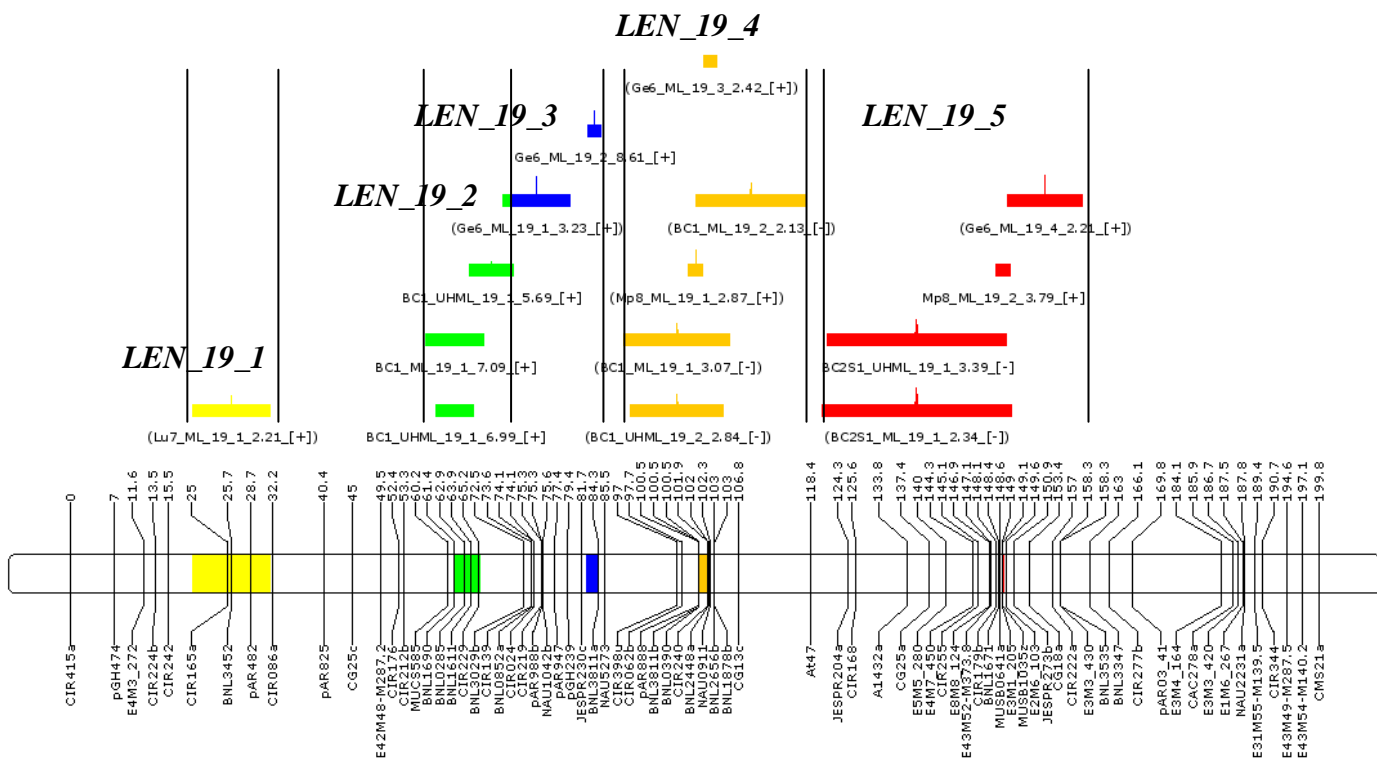



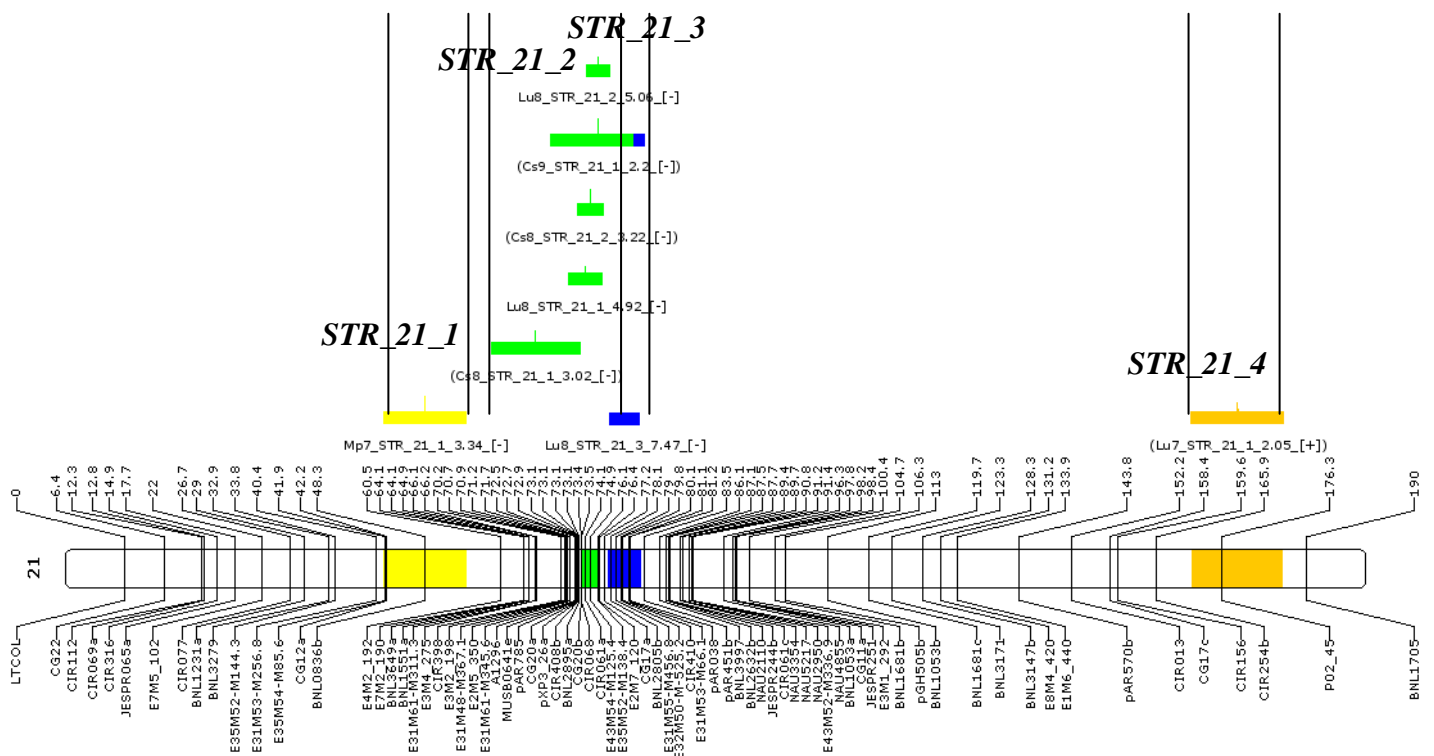

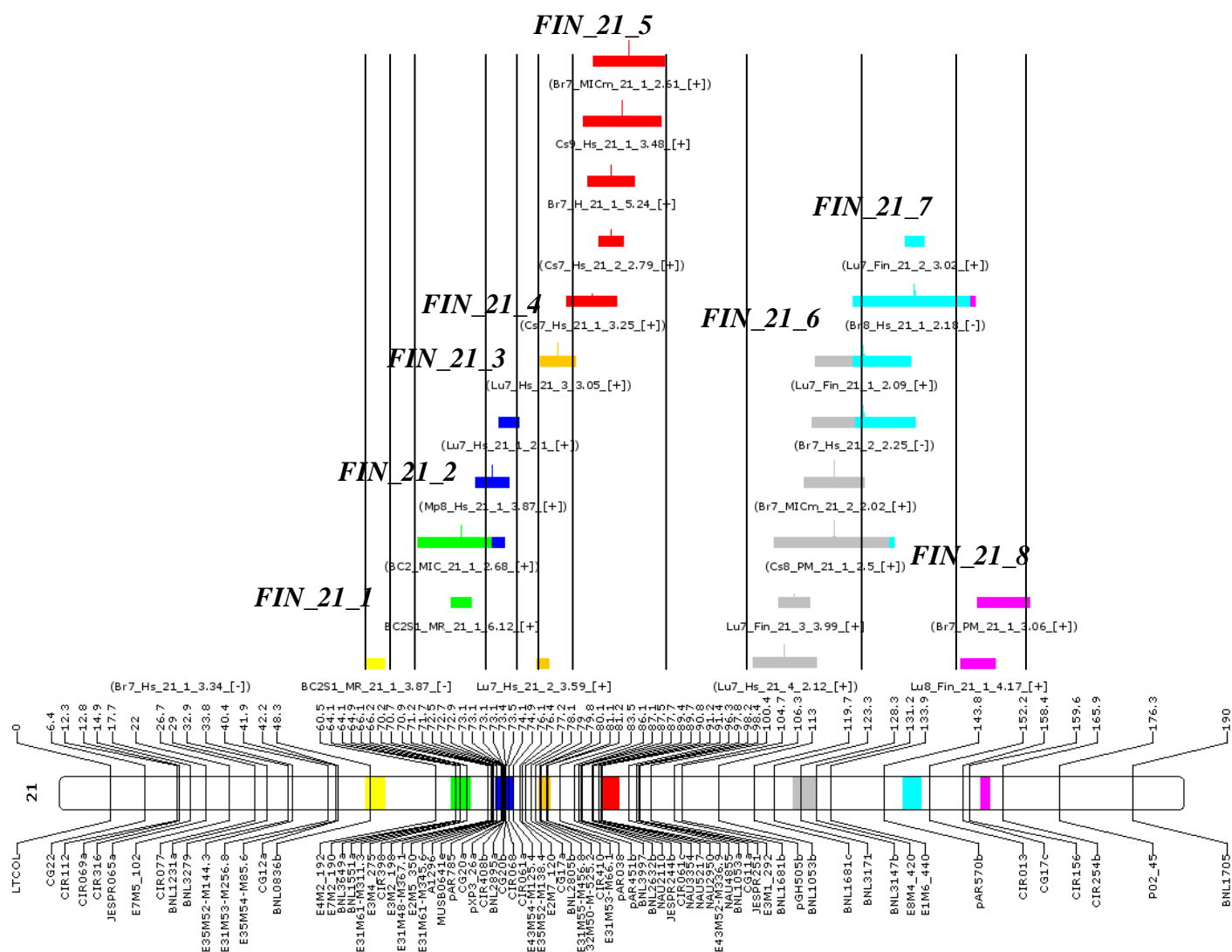



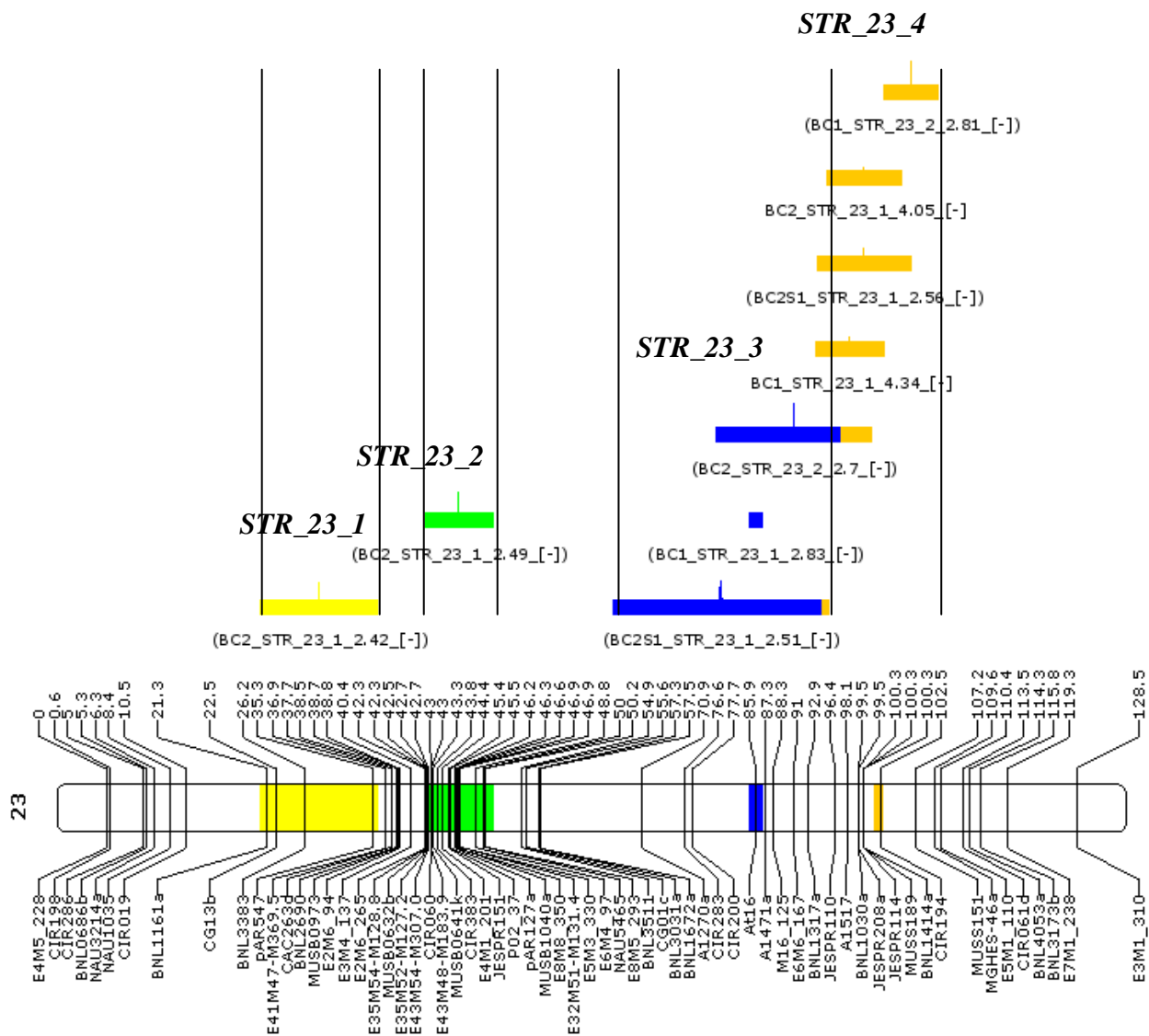

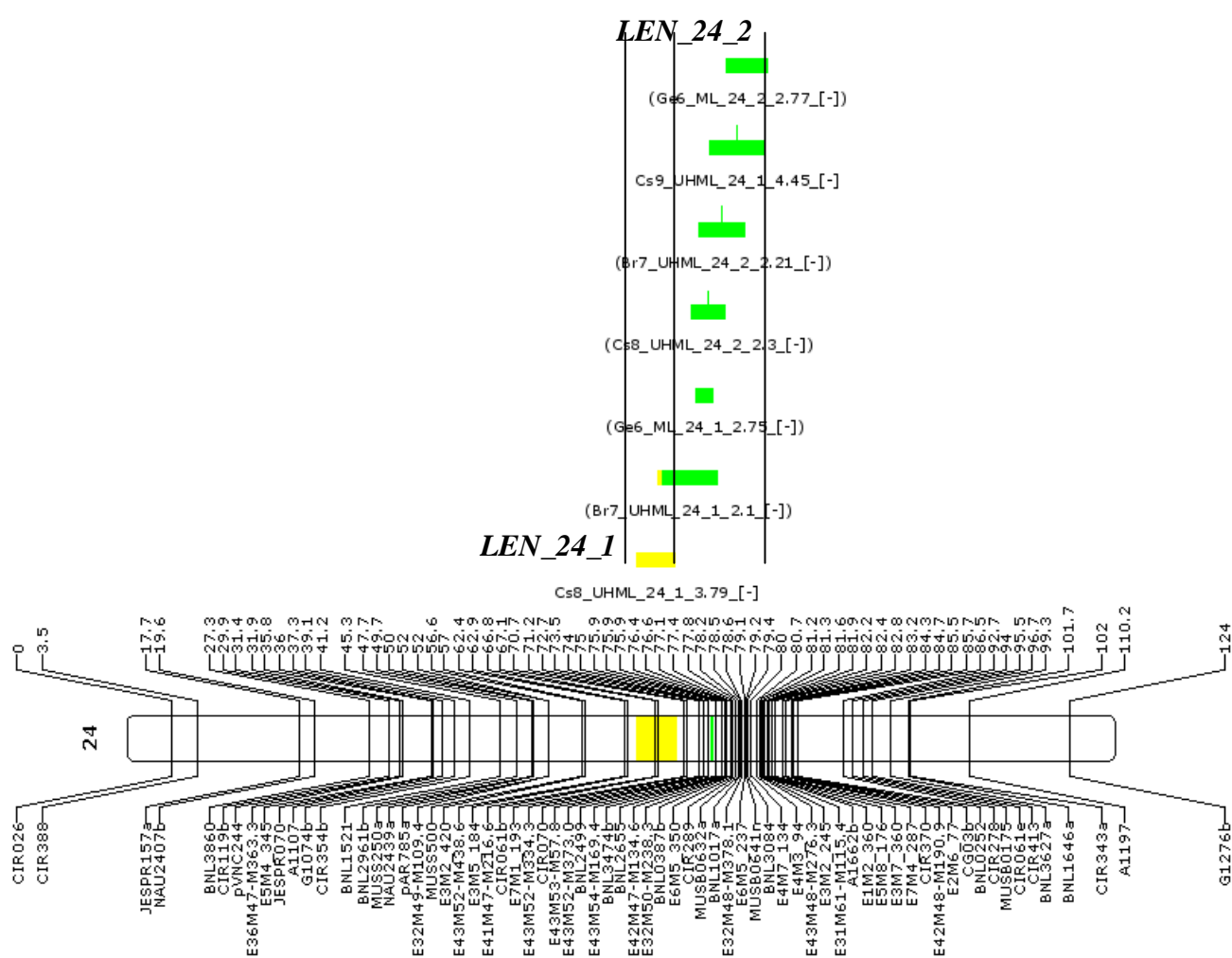

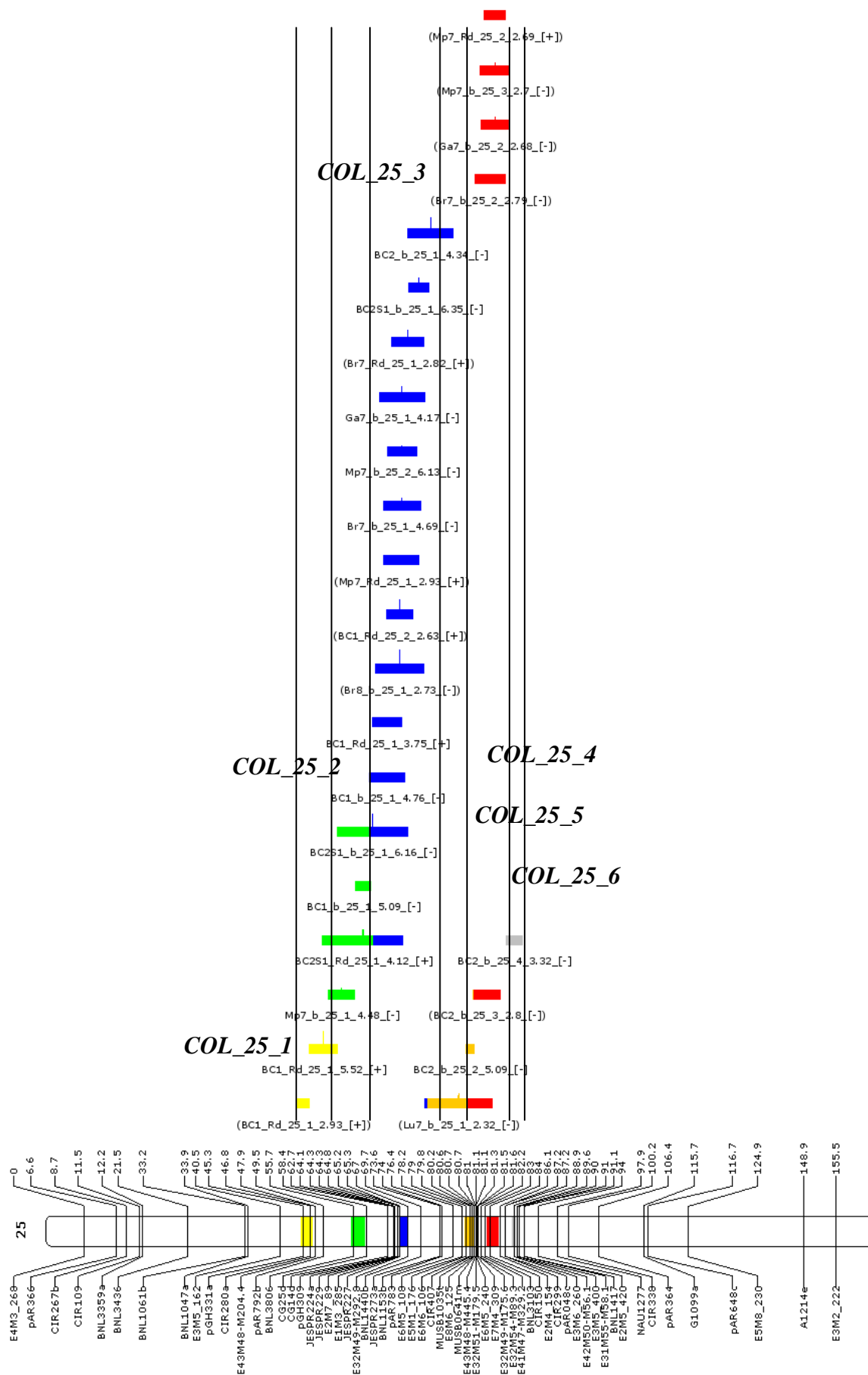

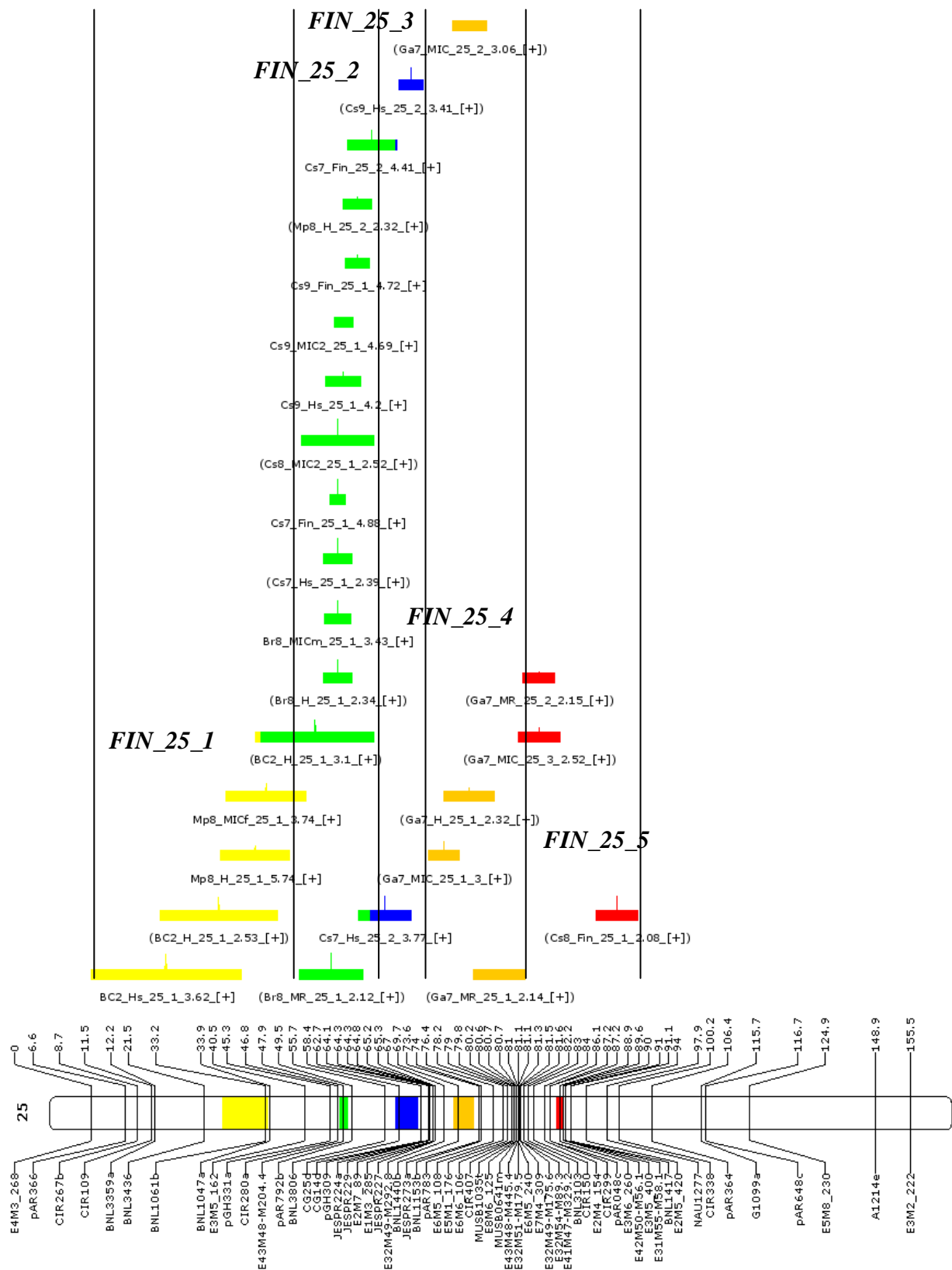

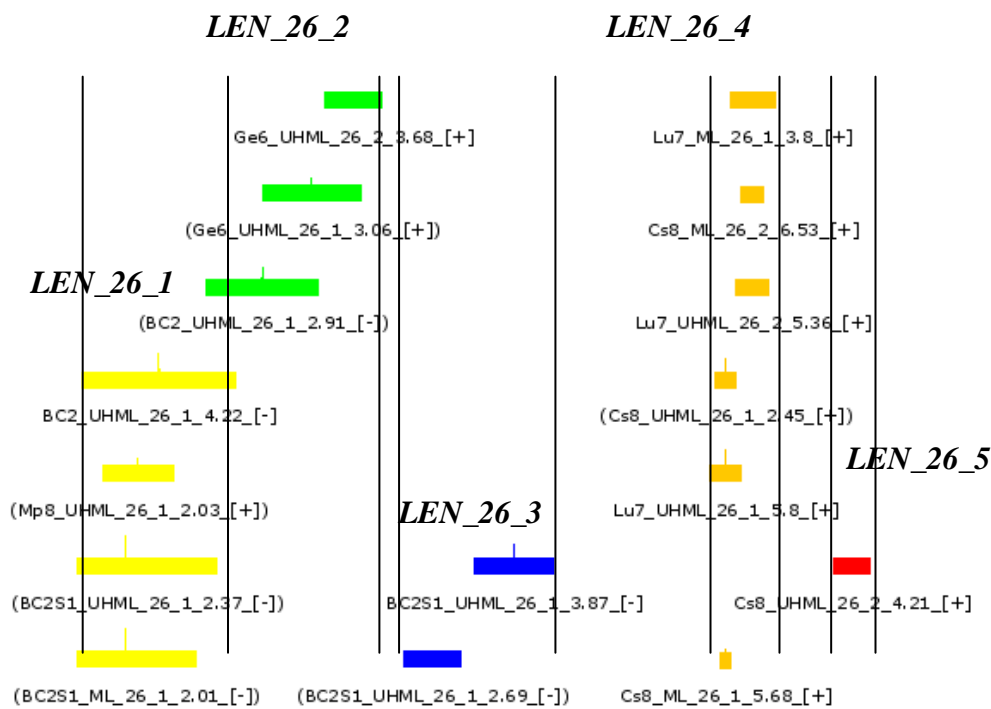

Supplement: Additional file 5 — Figure S2 (continuation of Figure 2): Same legend as Figure 2. [file 1471-2229-10-132-S5.PDF]
