# Supplementary material for: Meta-analysis of cotton fiber quality QTLs across diverse environments in a Gossypium hirsutum x G. barbadense RIL population
Source: BMC Plant Biol. 2010 Jun 28;10:132. doi: 10.1186/1471-2229-10-132 (PMC3017793; doi:10.1186/1471-2229-10-132)
Supplement: Additional file 6 — Table S4: Detailed chromosome by chromosome descriptions of the QTL clusters identified by MetaQTL. [file 1471-2229-10-132-S6.DOC]

**Additional file, Table S4: Detailed descriptions of the clusters as defined by MetaQTL software.** All chromosomal positions in cM refer to the BC-RIL consensus map from Lacape et al [39]. Tabulated synthetic results of clusters and meta-clusters are shown in Table 5 and chromosome displays as output figures by MetaQTL are shown in Additional files 3 and 5, Figures S1 and S2.

| Meta-analysis of fiber fineness and elongation on c2 |
| --- |
| An upper region (14-40 cM of the RIL map) hosted 18 LOD peak positions for fiber fineness traits, related to 5 different RIL data sets (Br7, Ga7, Cs9, Mp8; and Lu7) and 2 BC data set (BC_2_ and BC_2_S_1_). Except for Ga7, all peaks were of low LOD values and all contributions were of similar directionality (lower IM, MR and H by the *Gh* parent). The best clustering model by MetaQTL located 3 clusters for 17 peaks and an isolated peak from the BC_2_ set that mapped in a lower region of c2 (4^th^ cluster). The 3 clusters mapping between 23 cM and 43 cM of the consensus map may be coalesced in a single meta-cluster, *FIN_2*. Two fineness QTL of similar directionality, *FF02.1* and *MIC02.1,* were reported by [16] in the lower region of c2 where the solitary LOD peak from the BC2 population mapped.  Additionally, fiber elongation LOD peak values from 4 different RIL data sets (Cs8, Cs9, Br8 and Mp8), all significant and of same directionality (*Gh* increases trait), mapped in close proximity in a central region of c2. A single cluster positioned at 46 cM was delineated by MetaQTL, thus named *ELO_2*. Chee et al. [11] also reported an elongation QTL (*ELO2.1*) on c2, of similar sign of additivity, with large CI, and located near loci pGH399 and BNL3971 (46 cM). |
| Meta-analysis of fiber length and strength on c3 |
| A large region along the upper half of c3 (11-45 cM) contained 25 fiber length LOD peaks all with consistent directionality (higher length by *Gb* parent). An additional single peak (from Mp8) was isolated at the other end of the chromosome and was of reversed effect. LOD peaks were detected from 4 different RIL data sets (Ga7, Br7, Br8 and Lu7) as well as from the 3 BC data sets. High LOD scores were obtained for *Lu7-UQLw* (LOD7.6), *Ga7_ML* (5.5) and *BC_2_S1_UHML* (6.9). Average additive effect conferred by the *Gb* parent reached +1.3 mm as an average. The best clustering model by MetaQTL for this group identified 3 clusters, *QTLClust_LEN_3_1, _2* and *_3* centered at 12, 36 and 47 cM respectively. The 2 latter clusters are assumed to correspond to a single meta-cluster, *LEN_3*. A fiber QTL, *FL03.1(HVuhm,HVsl2.5)*, of similar directionality was also reported at a central location of c3 near locus pAR172 (35 cM) [13]. Park et al. [18] also mapped a length QTL on c3 (un-named and sign of additivity not reported) at a location near locus BNL0226 (63 cM) but this does not agree with any of our clusters.  Five RIL data sets (Br7, Br8, Lu7, Cs9 and Mp8) mapped co-aligned fiber strength LOD peaks on c3. All peaks were of similar directionality (better strength by *Gb*), but none were significantly above the permutation LOD threshold. Four additional strength LOD peaks from the BC data sets also mapped to c3, but at different locations. Among the 4 clusters proposed by MetaQTL, only the largest cluster (*QTLClust_STR_3_4*) located at 110 cM, between flanking SSRs, CIR133 and NAU2161, had sufficient support from more than one data set, and thus named *STR_3*. Kohel et al. [9] reported a strength QTL (*Sf-1* near locus BNL3259, at 91 cM) close to this last cluster.  Chromosome 3 also contained 15 fiber fineness LOD peaks. All showed favorable effect by the *Gb* parent (lower fineness), except two. LOD peaks were detected in 3 RIL data sets (Br7, Cs7 and Lu7) and 3 BC generations. However, their localization was mostly not congruent across populations or data sets, with 2 LOD peaks from the Cs7 data set (*QTLclust_FIN_3_1* at 0 cM), 9 LOD peaks from the BC data sets (*QTLclust_FIN_3_2* at 47 cM) and a group of 4 LOD peaks supported by data from 3 RIL and the BC_1_ (*QTLclust_FIN_3_3* at 101 cM). The clustering model (3 clusters) proposed by MetaQTL should therefore be considered with caution. Two reports for fineness QTL from the literature were found, *Ff-3*, near locus P09-53 (of homoeologous pair c3/c14, at around 66 cM) [9], and *FF03.1* near locus A1145 (57 cM) [16], but their localization did not agree with any of our tentative clusters. |
| Meta-analysis of fiber length on the bottom region of c4 |
| A lower region of c4 contained length LOD peaks originating from 6 different RIL data sets, Cs7, Cs8, Ga7, Lu7, Mp7, and Mp8; but none of BC origin. However MetaQTL was unable to project the LOD peaks from Mp7, Mp8, and Cs8. This is probably due to marker inversion between the RIL map and the consensus map in this region; however their location is within 4 cM of that of the other peaks. High LOD scores were associated with *Mp8_UHML* (LOD8.6) and *Ga7_ML* (6.6), and all additivity effects were of the same directionality, with positive effect from the *Gh* parent. Although the clustering was biased by the inability of MetaQTL to integrate all data, the meta-analysis by MetaQTL grouped the 4 peaks from Ga7, Cs7 and Lu7 data into a single cluster (at 67 cM, between CIR249 and NAU2231b), thus named *LEN_4*. Two length QTLs from the literature were reported in the same region, *FL04.1*, near locus A1310 (around 60 cM) [13] and for an un-named QTL in the interval G1033-A1172, or 30-50 cM of the consensus map [10]. |
| Meta-analysis of fiber fineness and length on c5 (indicative) |
| C5 contained 11 fineness and 11 length LOD peaks, split between 2 regions in the upper and lower sections, with 9 and 2 for fineness; and 6 and 5 for length respectively at either end of the chromosome. The peaks were derived from the BC_1_ data set and from 4 RIL data sets (Mp7, Mp8, Lu8 and Br7) for fineness; and from 5 RIL data sets (Cs7, Cs9, Mp7, Ge6 and Lu8) for length. The directionality of the peaks was not completely consistent, although the favorable effect at the majority of peaks derived from the *Gh* parent (lower fineness, higher maturity, micronaire and higher length). However, for both fineness and length, or for either of the 2 chromosomal segments, the co-localization of LOD peaks was not highly supported across different data sets so all of the clusters identified by MetaQTL must be considered indicative only. These clusters were reported because of the possible errors in the linkage map of c5, but, as indicated below, do have some additional support.  The clusters identified in the upper region of c5 are corroborated by several other QTL reported from the literature. For fiber fineness Draye et al. [12] and Rong et al. [16] reported 4 QTLs, *FF05.1*, *FF05.2*, *FF05.15* and *MIC05.1,* encompassing region between 22 cM (locus pAR931) and 70 cM (BNL852b). In the case of fiber length, Chee et al. [13] reported 2 QTLs, *FL05.1* and *FL05.2*, and Kohel et al. [9] reported one QTL, *Lf-2(t)*, close to maker BNL3029 (as a bridge locus mapped on homoeologous pair c5/c19 mapped at around 70 cM). Lastly, Jenkins et al. [21] in their report on chromosome-substitution lines (*Gb* into *Gh*), reported similar effects and directionality in terms of length and fineness in line CS-B05sh substituted with *G. barbadense* short arm of c5 into *G. hirsutum* as the remainder genome. |
| Meta-analysis of fiber color in the middle region of c6 |
| Five RIL data sets (Lu7, Ga7, Mp7, Br7 and Br8) and two BC data sets (BC_1_ and BC_2_S_1_) mapped fiber color LOD peaks along a central region of c6 (30-50 cM). Additive effects at all positions, except one (trait +b in BC_2_S_1_), were consistent with the *Gh* parent contributing favorably (higher reflectance and lower yellowness index). The 13 peak positions from this report were grouped as 3 clusters by MetaQTL, the third (at 51 cM, between BNL1440a and BNL3594b) alone containing 10 of the 13 QTLs, thus named *COL_6*. A fiber color QTL, *FC06.1*, with similar additive effect, was reported from the literature in the same region of c6, near loci A1208b or pAR03-32 at around 60 cM [6]. |
| Meta-analysis of fiber color in the middle and the top regions of c8 |
| Altogether 34 LOD peaks for either of the 2 color indices (Rd, +b) were mapped to this chromosome, 25 from the RILs and 9 from the BC data sets. Six of the 7 RIL data sets (all except Lu7) where fiber color was measured (fiber color was not measured in Lu8, Cs7, Cs8 and Cs9) contributed significant color LOD peaks. A LOD as high as 10.6, and a R² as high as 0.48 were recorded for *Ge6_Rd*. Additivity was consistent over all LOD peaks with favorable (higher reflectance and lower yellowness index) effects by the *Gh* parent. The best model generated by MetaQTL was eight separate clusters for fiber color on c8.  A 34 cM long region in the upper part of c8 (20-54 cM of the consensus map) contained 20 of the 34 peaks with a good representation of various data sets. Of the 4 fiber color clusters proposed by MetaQTL in this region, three (*QTLClust_COL_8_2* to *_4*) occur in a fairly short distance (located at 46, 51 and 54 cM) and accounted for 17 of the 20 peaks, and could probably be coalesced into a single meta-cluster (*COL_8A*). Another group of 14 color LOD peaks were mapped in the lower region of c8 (80-120 cM). They originate from 2 RIL (Mp7 and Br8) and 2 BC data sets (BC_1_ and BC_2_S_1_). Although they displayed consistent directionality similar to that in the upper region of the chromosome (higher Rd and lower by *Gh* parent) the co-localization was not obvious and their clustering with MetaQTL inferred 4 possible cluster, mapped between 84 and 121 cM, that can only be considered as putative, unless the group of 4 could be coalesced into a single meta-cluster (*COL_8B*). The fiber color QTL (*FCA02.1*) reported on c8 (named A02) in [6] has similar directionality (higher yellowness by *Gh*) and its LOD values was particularly high (LOD11.7). This QTL was located near locus pGH232a (their map), or pAR785b as a bridge locus at 93 cM, i.e. between clusters *QTLClust_COL_8_6 and _7* within the QTL-rich lower region of c8. |
| Meta-analysis of fiber fineness and length in 2 regions of c9 |
| Two regions of c9 showed a high density of LOD peaks for fiber fineness (25 peaks) and length (8): an upper region contained LOD peaks for both length and fineness traits, and a lower region contained only fineness LOD peaks. An overall consistency in directionality was observed with favorable effects by *Gh* for length and by *Gb* for fineness (lower micronaire and fineness).  The upper region of c9 mapped 12 fineness and 8 length LOD peaks. Fineness LOD peaks were detected from 5 RIL data sets (Br7, Br8, Cs9, Mp8, Ge6) and 1 BC data set (BC_2_S_1_). Of the 3 clusters, the 2^nd^, *QTLClust_FIN_9_2* mapped at 41 cM, is the best supported. However we propose that a meta-cluster (named *FIN_*9A) groups the 3 clusters (mapped at 20, 41 and 51 cM). Length LOD peaks in the same region were detected from 4 RIL data sets (Lu7, Mp8, Ge6, and Cs8, some of which were of high significance (highest LOD for *Lu7_UQLw* was 9.5). As for fineness, of the 3 clusters (mapped at 18, 36 and 39 cM) proposed in this region by MetaQTL, and although the 2^nd^ is the best informed, we propose that they coalesce as one meta-cluster (*FIN_9A*). Lin et al. [14] also reported a length QTL, *qFL3*, with a high LOD of 5.5, but its effect was opposite to that reported here (lower length by *Gb* alleles) and chromosome assignment of their LG21 as c9 deserve further verification. Finally, Frelichowski et al. [15] also reported an un-named length QTL on c9, but location near locus MUSS022, at 104 cM, differed from ours.  In the lower half of c9, 3 RIL data sets (Lu7, Mp8, Ga7) mapped 12 fineness LOD peaks. One RIL data set (Lu7) reached high LOD scores (as high as 8.9 for *Lu7_MR*). Directionality of the LOD peaks was similar to the upper region of c9 (lower micronaire, maturity and fineness by the *Gb* parent). Ten of 12 peaks were grouped in 2 possibly coalesced clusters in close proximity (meta-cluster *FIN_*9*B*), mapped at 95 and 102 cM (with 3 additional solitary LOD peaks in the middle of the chromosome). A fiber fineness (lower micronaire by *Gb* parent) QTL, *FF09.1*, was also reported near locus P10-62 (91 cM) as a bridge locus [6]. |
| Meta-analysis of fiber fineness in the middle region of c10 |
| A total of 16 fineness LOD peaks were detected on c10, 7 from the RIL experiments (in data sets Lu7, Mp7, Cs8 and Cs9) and 9 from 2 BC generations, BC_1_ and BC_2_S_1_. Directionality was congruent with positive (lower micronaire and fineness) contributions by the *Gb* parent. However most of the peaks were non-significant (relative to the permutation threshold) and except for the 9 peaks in the central region had only minimal representation across different data sets (4 RILs and 1 BC data set). These 9 LOD peaks corresponded to the 3 central clusters, out of the 6, identified by MetaQTL: *QTLClust_FIN_10_3* to *_5* mapped at 58, 70 and 81 cM, respectively, and possibly coalesced in one meta-cluster, *FIN_10*. A fineness QTL (*FF10.1*), of large CI and located near locus pVNC163b (extrapolated at around 60 cM), was associated with lower fineness by *Gb* [12]. The 7 other fineness LOD peaks mapping along the top and bottom regions were only detected in the BC data sets; therefore, the 3 corresponding fineness clusters on c10 should be considered with caution. |
| Meta-analysis of fiber fineness in the upper region of c12 |
| Twenty fiber fineness LOD peaks mapped to c12. They originated only from the RIL data sets, but had broad representation across sites and environments as 9 (Br7, Br8, Cs7, Cs8, Cs9, Ga7, Ge6, Mp8 and Lu7) out of the 11 different data sets were present (only Lu8 and Mp7 were missing). All were consistent with positive *Gb* contributions (lower micronaire, fineness and maturity). Best congruence was observed for the 11 peaks from 6 different RIL data sets (Br8, Cs7, Cs8, Cs9, Lu7, and Mp8), which mapped within a 15 cM long region on top of c12. MetaQTL assigned these 11 peaks to two nearby clusters, *QTLClust_FIN_12_1* and *_2* mapped at 4 and 14 cM, that may be coalesced into a single meta-cluster (*FIN_12*). Two fineness QTLs have also been reported in the literature, *FF12.1* (near locus A1252 at 23 cM) [12] and *FF12.15* (near pAR03-42 at 17 cM) [6].  The distribution of the 9 other fineness LOD peaks within a central region of c12 was not congruent: the 4 additional fineness clusters *QTLClust_FIN_12_3* to *_6* should therefore be considered with caution. Similarly, the distribution of LOD peaks for other traits (5 for elongation, 5 for color, 3 for length and 3 for strength) mapping to this chromosome (but not included in the meta-analysis) was fairly scattered.  c12 also contained 14 fiber length uniformity peaks (higher uniformity by *Gh* parent), but their co-localization was only moderate and MetaQTL predicted 8 clusters as the best model (not shown). This enrichment in length uniformity QTLs on c12 was partially corroborated by the presence of 4 length uniformity QTLs, *SF12.1, FU12.1, FU12.2* and *FU12.3*, reported in [13]. |
| Meta-analysis of fiber fineness and elongation in the middle region of c15 |
| A fairly large central region of c15 (40-110 cM of the consensus map) contained 30 fineness LOD peaks of large representation across data sets, including 8 different RIL data sets (Cs7, Cs8, Cs9, Lu7, Lu8, Ga7, Br7 and Br8), and 2 BC data sets (BC_2_ and BC_2_S_1_). Some peaks were of high significance (LOD6.0 for *Lu7_MIC* and LOD7.5 for *Ga7_H*). The best clustering model in MetaQTL predicted 6 clusters, of which 4, namely *QTLClust_FIN_15_2* to *_5*, encompassed 25 peaks (detected from 7 RIL data sets) with overlapping CI and mapped at 62, 67, 73 and 84 cM respectively, and may be coalesced in one meta-cluster (*FIN_15*). For all these peaks, additivity contributions are congruent, indicating positive effects by the *Gb* parent (reduced values of fineness, maturity or micronaire). Three fineness QTLs from the literature (*FF15.1*, *FF15.01,* and *FF15.05*) were identified in the same central region of c15 [12, 16], between locus A1553 (77 cM) and locus pAR077a (117 cM), with a similar directionality as in this report for 2 of them.  Notably, 10 LOD peaks (detected in 4 different RIL data sets, Br7, Br8, Ga7, and Cs8) for fiber elongation also mapped in the same central region of c15. All the elongation QTLs displayed similar directionality (higher values of elongation by +0.2 units on average by *Gh* parent). Best clustering model by MetaQTL predicted 4 clusters for fiber elongation, of which only the 2 central ones, located at 67 and 72 cM, were sufficiently supported by several data sets and coalesced as one meta-cluster, *ELO_15*. Park et al. [18] reported an elongation QTL at a location within cluster *QTLClust_ELO_15_2* (near locus MUSS572 of their map, ie close to MUSS422 at 70 cM. |
| Meta-analysis of fiber fineness in the middle region of c16 (indicative) |
| Fiber fineness LOD peaks (10) on c16 originated from 6 different RIL data sets (Mp8, Br7, Lu7, Cs7, Cs8 and Ga7). All, except one, were associated with *Gb* allelic positive effects (lower fineness and micronaire). However positions were fairly scattered along the chromosome, and the clustering results produced by MetaQTL (6 clusters) must be viewed cautiously, unless the 3 upper clusters (*QTLClust_FIN_16_1* to _3) representing only 3 RIL data sets and mapped at 41, 50 and 64 cM, be coalesced. Although indicative, this region is corroborated by 2 fineness QTLs, *MIC16.1* and *FF16.1*, reported in Rong et al. [16] and mapped between 40 to 60 cM in the middle of c16. |
| Meta-analysis of fiber fineness in the middle region of c17 |
| A central region of c17 contained 14 fineness LOD peaks from 6 different RIL data sets (Ga7, Cs7, Br7, Br8, Lu8 and Mp7), but none from the BCs. All peaks were associated with *Gb* positive allelic effects (lower fineness, micronaire and maturity) and mapped in fairly close proximity (region 18-31 cM). The 14 LOD peaks had fairly overlapping CI. Their grouping in 2 clusters by MetaQTL mapped at 18 and 28 cM, is thus assumed to represent a single meta-cluster, *FIN_17*. Three QTLs, *FF17.1*, *MIC17.2* and *FF17.2*, of similar directionality (lower micronaire by *Gb* allele), high LOD value (reaching LOD9.8) were reported near locus pAR250 of their map and pAR172 at 24 cM as a bridge locus [6, 16]. |
| Meta-analysis of fiber fineness on c18 |
| Seven RIL data sets (Ga7, Cs7, Cs9, Lu7, Lu8, Mp7 and Mp8), 2 BC data sets (BC_1_ and BC_2_S_1_) mapped 17 fineness LOD peaks on c18, the majority of which associated with *Gb* positive allelic effects (lower fineness, maturity and micronaire). Their scattered distribution along the chromosome resulted in 5 clusters as the best clustering model by MetaQTL: only the cluster *QTLClust_FIN_18_3* located at 49 cM was supported by a good representation of data sets (5 RILs and BC_1_). Of the two fineness QTLs reported in the literature on c18, *FF18.1* and *FF18.2* [12] only the 1^st^ (near locus pAR788 at 31 cM) fairly agreed in position with *QTLClust_FIN_18_3,* but was of the opposite effect (higher fineness by *Gb*). |
| Meta-analysis of fiber elongation, length and fineness in a middle region of c19 (indicative) |
| Although c19 was highly populated in LOD peaks for fiber elongation (14), fiber fineness (18) and fiber length (15) their distribution was scattered and the allelic effects were sometimes contradictory. The central region of c19 (45 to100 cM out of the total 200 cM) showed some level of consistency. In this region, the 14 fiber elongation peaks, all of positive contribution by the *Gh* parent, were derived from 3 RIL data sets (Ga7, Br7 and Br8) and 3 BC data sets. The grouping by MetaQTL into 5 clusters appeared of limited utility, except if some mapping error occurred on this chromosome. Two 2 elongation QTLs were also reported in the literature, but both had large CI and were localized at different locations: *ELD08.1*, near locus pAR482 at 29 cM, and *ELD08.2*, near locus pAR570b at 144 cM [11].  Similarly, 15 length LOD peaks, from 3 RIL (Lu7, Ge6 and Mp8) and 2 BC (BC_1_ and BC_2_S_1_) data sets were also mapped to c19 but their clustering (5 clusters by MetaQTL) was probably not reliable. Additivity was consistently of *Gh* origin for upper clusters of the central region, while contradictory effects were observed in the lower clusters. Despite the poor level of co-localization, high LOD values were observed (LOD8.6 for *Ge6_ML* and LOD7.1 for *BC1_ML*) among peaks within the upper fiber length clusters. Two length QTLs reported in [13] fairly encompass the same region of c19, *FLD08.1* near locus P2-9 (their map) at around 40 cM and *FLD08.2* near locus pAR137 at (their map) at around 100 cM. The 1^st^ one had an R² of 0.30 highest of all length QTLs (LOD scores not reported) in [13], but the additive effect (*Gb* increase) was reversed compared to the RIL and BC peaks in this cluster.  Among the 18 fineness-related LOD peaks mapped to c19 (10 from the RILs and 8 from the BCs), 12 mapped within the same central region as the elongation and length QTLs. Positive contributions clearly derived from the presence of *Gh* parent alleles (lower micronaire and fineness and higher maturity). Altogether the LOD peaks related to 6 different RIL data sets (Ge6, Cs8, Mp7, Mp8, Lu7 and Lu8) and 2 BC data sets (BC_1_ and BC_2_), but consistency in mapping was not satisfactory. The 12 peaks in a central region were grouped as 4 clusters by MetaQTL and mapped between 51 and 102 cM, Three other fineness clusters were predicted at each end of the chromosome but these were not highly supported (BC_2_-only at the top and 4 RIL data sets at the bottom). Three fineness QTLs, *FFD08.1, FFD08.2* and *MICD08.1*, were previously reported by [12] in the same central region (near locus G1112 or pAR825 at 40 cM) of c19, but their effects were reversed (lower fineness by *Gb* parent). Finally, we also mention of a QTL named *FFD08.4* reported by [12], near locus pAR03-41 at 170 cM, corroborating location and directionality of the clusters at the bottom of c19.  Overall, c19 contained numerous LOD peaks and QTLs along its entire length (highest number detected in both our BC data sets and QTL data compiled by [16]), for which it may be assumed that the presence of *Gh* alleles in the central region (40-100 cM) simultaneously and favorably impacted the 3 groups of fiber parameters, elongation, length and fineness. However within this fairly large region, the clustering (probably 3 for each of the 3 categories of QTLs) predicted by MetaQTL was not entirely satisfactory, possibly due to remaining mapping errors in this central region of the consensus map of c19. |
| Meta-analysis of fiber strength (indicative) and fineness on c21 |
| Seven of the 8 strength LOD peaks mapped along a central region of c21. They originate from 4 RIL data sets (Mp7, Cs8, Cs9 and Lu8). An additional co-aligned LOD peak at LOD1.9, just below the LOD2 cuttoff, from a 5^th^ data set, Ge6, also mapped to this region but was not shown. Another strength LOD peak from the Lu7 data set mapped at the bottom of the c21. For all these peaks, the *Gb* parent increased the trait value. Among the 3 clusters identified by MetaQTL in this central region, only LOD peaks within *QTLClust_STR_21_2* and *_3*, with overlapping CI, mapped 5 cM apart (75 and 80 cM), together represented a putative (supported by only 3 RIL data sets) localization for a meta-cluster, *STR_21*. Two fiber strength QTLs have also been reported on c21 in the literature from 2 independent populations, *FSD02.1* originally un-named [6] and *FSD02.2(STR)* initially reported as *STR-D02* by [8], both with large CI and associated with *Gb* positive effects on fiber strength, and mapping also in the center of c21 (near locus A1296 at 73 cM for the 1^st^ one and near locus pAR038 at 80 cM for the 2^nd^ one).  The same central region of c21 also contained 12 fineness LOD peaks (out of a total of 23 on c21) from 5 RIL (Lu7, Cs7, Cs9, Mp8 and Br7) and 2 BC (BC_2_ and BC_2_S_1_) data sets. Parental effects were congruent for a positive contribution by *Gb* alleles (lower fineness, maturity and micronaire). Four clusters, *QTLClust_FIN_21_2* to *_5*, mapped at 65, 73, 81 and 93 cM, comprised 11 LOD peaks with mostly overlapping CI, thus proposed as one meta-cluster *FIN_21A*. One fineness QTL reported by Draye et al. [12], *FFD02.1,* near locus A1296 at 73 cM, also occurred in the same region.  The bottom of c21 also appeared dense in LOD peaks for fiber fineness (10). Although CI of individual LOD peaks overlapped, 3 separate clusters, *QTLClust_FIN_21_6* to *_8*, mapped at 129, 149 and 163 cM*,* were predicted by MetaQTL. Directionality was the same as above (lower fineness and micronaire by *Gb* parent) and LOD peaks originated from 5 RIL data sets (Br7, Br8, Cs8, Lu7 and Lu8). A second meta-cluster, *FIN_*21B, is proposed for this group of LOD peaks. Two fineness QTLs have been reported in the literature, *FFD02.15* and *FFD02.2* by [12, 16], both within the same region between loci BNL3171 at 123 cM, and pAR570 at 144 cM  Contributed by only 3 RIL data sets (Lu7, Br7, Br8), 6 fiber length LOD peaks were located in the same central region (72 to 89 cM) of c21 as the strength and fineness clusters. One QTL from the literature corroborated our data, *FLD02.2(Hvsl2.5)* near A1296 at 73 cM [13], however directionality in our data indicated a positive contribution by the *Gh* parent in Br7 and Br8 data sets and by the *Gb* parent in Lu7 data set, so the meta-analysis was not conducted. |
| Meta-analysis of fiber length and strength (indicative) in the lower region of c23 |
| c23 represented a peculiar situation where QTLs for fiber strength and length were consistently detected at fairly high LOD scores in the 3 BC generations, but were only poorly confirmed in the RIL experiments. For length, the nine LOD peaks from the 3 BC data sets detected along a bottom part of c23 were corroborated by 2 RIL data sets (Br8 and Ga7). A solitary peak from a 3^rd^ RIL data set (Lu8) mapped to the top of c23. Positive contributions derived from *Gb* parent. The 9 LOD peaks in the bottom region grouped in 2 clusters by MetaQTL, but their CI essentially overlapped, thus representing a possible meta-cluster, *LEN_23*. The 2 clusters mapped at 98 and 117 cM. Chee et al. [13] reported a fiber length QTL, *FL23.1*, of similar directionality, but at a different location, near locus pAR547 at 35 cM  c23 also contained 9 fiber strength LOD peaks, all uniquely of BC origin, and hence not confirmed by any of the RIL experiments. Seven mapped in a lower region, were grouped as 2 nearby clusters (86 and 102 cM) possibly coalesced as one meta-cluster, *STR_23*. Two fiber strength QTLs were reported in the same region by [6], as *FS23.2* near pAR209 (their map) or A1517 at 98 cM*,* and by [14], as *qFS2*, but in this last case the chromosome assignation needs confirmation and the *Gb* parent decreased trait value. |
| Meta-analysis of fiber length in the middle region of c24 |
| Four RIL data sets (Ge6, Br7, Cs8 and Cs9) mapped seven fiber length LOD peaks along a central region of c24. The *Gb* parent increased the trait value in all cases. The best model for clustering by MetaQTL resulted in 2 probably coalesced clusters (proposed as one meta-cluster, *LEN_24*), mapped at 67 and 75 cM respectively. A fiber length QTL, *FLD03(Hvi2.5HVuhm,Lw),* with increased length by the *Gb* allele, has also been reported [13, 16] but at a different location in the upper part of c24, near pAR571a (their map) and G1074b as a bridge locus at 39 cM.  Although 12 LOD peaks for fiber fineness also mapped to c24, originating from 2 RIL data sets (Ge6 and Lu7) and 2 BC data sets (BC_2_ and BC_2_S_1_), the clustering was not considered due to contradictory phenotypic effects. |
| Meta-analysis of fiber color and fineness in a middle region of c25 |
| The upper central region of c25 was particularly dense in LOD peaks for 3 fiber trait categories, color (25 peaks), fineness (25) and length (7). Worth noting was the fact that all these peaks were completely congruent in terms of directionality of allelic effects, with *Gh* parent contributing positively (increase of Rd, or decrease of +b) at all fiber color peaks, and *Gb* parent contributing positively at all fineness (including lowered fineness, maturity and micronaire) and length peaks, in agreement with the associated phenotypic values of the 2 parents (*Gh* donor for fiber color, and *Gb* donor for fineness and length).  The 2 fiber color indices, reflectance (Rd) and yellowness (+b), mapped 25 LOD peaks within the center of c25 (45-90 cM). Peaks were detected in 5 RIL data sets (Mp7, Br7, Br8, Ga7 and Lu7), and all 3 BC data sets. Although a model with 6 clusters was the best model proposed by MetaQTL, the distribution of peaks and clusters indicated that peaks may be shared in 2 groups separating the 3 upper (17 LOD peaks) from the 3 lower clusters (8). We assume these 2 groups represent 2 separate meta-clusters both with large representation among data sets. Upper meta-cluster, *COL_25A*, represented LOD peaks of clusters *QTLClust_COL_25_1* to *_3* (46, 57 and 66 cM) reaching high significance (4 exceeded LOD5), whereas LOD peaks in the lower part (2^nd^ meta-cluster named *COL_25B* for clusters *QTLClust_COL_25_4* to *_6* mapped at 80, 84 and 89 cM) were of lower significance (all except 1 to be considered as putative). A fiber yellowness QTL, with similar effect (decrease in +b by *Gh* allele) and a LOD value as high as LOD9.2, has been reported as unnamed on c25 by [6] and renamed *FC25.1* in [16], and was located, near locus pGH309 at 64 cM.  Half (14 out of 25) of the fiber fineness LOD peaks on c25 were located within a central region. Five clusters were assembled by MetaQTL, of which *QTLClust_FIN_25_2* and *_3* mapped at 48 and 60 cM, grouping these 14 peaks are proposed as a meta-cluster, *FIN_25*. The LOD peaks originated from 5 RIL (Cs7, Cs8, Cs9, Br8 and Mp8) and BC_2_ data set. A fineness QTL (*FF25.1*) reported in [12], of large CI, mapped near locus pGH309 at 64 cM.  Lastly, 5 length LOD peaks of moderate significance (all putative) originating from 3 RIL data sets (Mp7, Ge6 and Cs8), delineated a fairly narrow region (75 to 85 cM) in the center of c25, but were not considered for clustering.  The observation that *Gb* alleles on c25 favorably affected overall fiber quality parameters was also reported in [21]. The substitution line CS-B25 having, within a *G hirsutum* genetic background, this chromosome pair replaced by the *G. barbadense* c25, presented additive and positive effects for micronaire (lower), length and strength (higher). Variation in fiber color observed in this material was not reported in [21]. |
| Meta-analysis of fiber length on c26 (indicative) |
| The 16 fiber length LOD peaks were derived from 4 RIL (Ge6, Cs8, Lu7 and Mp8) and 2 (BC_2_ and BC_2_S_1_) BC data sets. Although additive effects were all of similar directionality (positive by *Gh*) the level of co-localization was only poor and clustering model (5 clusters) predicted by MetaQTL is to be considered indicative. The previously reported QTL *FL26.1(Hvsl2.5uhmlLw)* [13], near locus pAR101 (their map) or pAR807 uppermost (0 cM) bridge locus, had a large CI but was of reversed additive effect (higher length by *Gb*). |
